# Mutational patterns and ancestry-linked profiles in a large hepatocellular carcinoma and combined hepatocellular–cholangiocarcinoma cohort☆

**DOI:** 10.1016/j.esmoop.2025.106048

**Published:** 2026-01-20

**Authors:** C. Gerdes, S. Rengarajan, K. Murugesan, J.S. Ross, S. Bartels, A. Vogel, A. Saborowski

**Affiliations:** 1 Department of Gastroenterology, Hepatology, Infectious Diseases and Endocrinology, Hannover Medical School, Hannover, Germany; 2 Princess Margaret Cancer Centre, University Health Network, Toronto, Canada; 3 Foundation Medicine, Inc., Boston, USA; 4 Upstate Medical University, Syracuse, USA; 5 Institute of Pathology, Hannover Medical School, Hannover, Germany; 6 Division of Gastroenterology and Hepatology, Toronto General Hospital Medical Oncology, University of Toronto, Toronto, Canada; 7 Research Training Group 2978, Hannover Medical School, Hannover, Germany

**Keywords:** targeted therapy, immunotherapy, genomic alterations, liver cancer, precision medicine

## Abstract

**Background:**

Despite significant therapeutic advancements, hepatocellular carcinoma (HCC) remains a highly fatal malignancy. To accelerate the development of targeted therapies, a comprehensive understanding of the spectrum of genomic alterations (GAs) is essential. Here, we present what is, to our knowledge, the largest genomic analysis of real-world HCC and combined HCC–cholangiocarcinoma (cHCC–CCA) patients.

**Patients and methods:**

Tumor samples from 2372 HCC patients and 150 patients with cHCC–CCA underwent genomic profiling using the FoundationOne® platform covering >290 genes, as well as tumor mutational burden (TMB) and microsatellite status.

**Results:**

Our comprehensive and representative analysis included 1793 male and 577 female patients across five genetic ancestries. Female patients exhibited lower frequencies of GAs in *TERT*, *MYC*, and *CTNNB1,* with higher rates of *BAP1* GA. Patients of East Asian ancestry presented an increased frequency of *TP53*, *MUTYH*, and *TET2* GAs, as well as a higher proportion of TMB-high tumors. Compared with HCC, cHCC–CCA exhibited higher frequencies of *IDH1* (8.0% versus 0.3%), *IDH2* (2.7% versus 0.04%), and *FGFR2* (7.3% versus 0.3%) alterations. Potentially actionable alterations were detected in 19.5% of HCC patients and 34.7% of mixed histology. Histological re-assessment due to detection of GA uncommon for HCC was carried out in 117 patients, resulting in a change in diagnosis in 37 cases. Limitations include the absence of detailed clinical data and dependence on the CE-certified Foundation Medicine (FMI) platform for functional annotation of detected variants.

**Conclusions:**

Our study represents one of the largest cohorts of HCC and cHCC–CCA patients with genomic data and highlights the critical role of integrated molecular diagnostics. Beyond uncovering therapeutic targets, next-generation sequencing profiling offers significant benefits in improving diagnostic accuracy for liver cancer.

## Highlights

• We present a granular analysis of GAs from clinical-grade sequencing of 2372 HCC and 150 HCC–CCA patients.
• Mutational profiles vary based on sex and genetic ancestry.
• Genomic profiling should be standard of care in HCC–CCA due to the high prevalence of actionable alterations.
• Critical review of NGS results may lead to re-consideration of the original diagnosis.
• Our study serves as a representative reference dataset for stratifying patients according to their GAs.

## Graphical abstract

**Figure:**
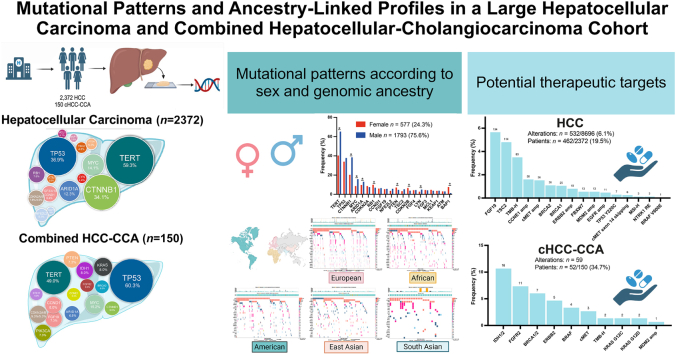

## Introduction

Hepatocellular carcinoma (HCC) is the most common primary malignancy of the liver and the third most common cause of cancer-related death worldwide,^1^ with an overall 5-year survival <20%.^2^ Due to the late presentation of symptoms, only a fraction of HCC patients qualify for potentially curative surgical or local treatments. For patients with unresectable or advanced disease, immunotherapy-based combinations consistently demonstrated superiority over tyrosine kinase inhibitors (TKIs) in pivotal phase III trials and have consequently evolved as the new standard of care, replacing multi-TKIs in the first line of systemic therapy.^3^^,^^4^ In recent years, the integration of next-generation sequencing (NGS) into routine clinical practice has revolutionized personalized treatment in several types of cancers, providing significant benefits in both diagnostic and therapeutic decision making. Although HCC was one of the first malignancies in which molecular targeted agents, such as sorafenib or lenvatinib, became a mainstay of systemic therapy, these agents were not biomarker guided and there are currently no genotype-matched therapies that are broadly established in this malignancy.

Nevertheless, the field is evolving, and novel approaches, e.g. targeting components of the WNT/CTNNB1 pathway or fibroblast growth factor receptor (FGFR) in FGF19-overexpressing tumors, suggest that precision oncology might also gain importance in HCC.

To advance and prioritize the development of targeted treatments in genetically defined subgroups of HCC, and to delineate how clinical demographics correlate with genomic patterns, a comprehensive understanding of the prevalence and spectrum of genomic alterations (GAs) in a global HCC cohort is essential. Here, we present what is, to our knowledge, the largest genomic analysis from a real-world cohort including 2372 HCC and 150 combined HCC–cholangiocarcinoma (cHCC–CCA) patients. Our work provides a genomic characterization of HCC of unprecedented granularity, extending to distinct ancestry-related profiles, using broad and CE-certified genomic profiling assays.

## Patients and methods

### FoundationCORE database

Samples from patients in the United States were submitted for comprehensive genomic profiling. This analysis includes sequencing data from tissue specimens from 2372 HCC cases and 150 mixed HCC–CCA cases, as diagnosed by the treating physician and confirmed on hematoxylin–eosin-stained slides. NGS was carried out using the hybrid capture, DNA-based FoundationOne® (*n* = 867 patients) and FoundationOne®CDx (F1CDx, *n* = 1505) assays (Foundation Medicine, Inc., Boston, MA) as described previously, in a Clinical Laboratory Improvement Amendments (CLIA)-certified and College of American Pathologists (CAP)-accredited laboratory, between May 2007 and March 2023.^5^ The FoundationOne® panel was used on samples collected between May 2007 and 26 August 2020, whereas FoundationOne®CDx testing was used from 27 August 2020 onward.

The analysis was focused exclusively on GAs considered functional or pathogenic in literature and seen in the Catalogue of Somatic Mutations in Cancer (COSMIC) repository, or those with a likely functional status (e.g. frameshift or truncation events in tumor suppressor genes). Variants of unknown significance (VUS) were excluded unless specified.

Research was conducted in accordance with the Declaration of Helsinki and Istanbul. Approval for this analysis, including a waiver of informed consent and a Health Insurance Portability and Accountability Act (HIPAA) waiver of authorization, was obtained from the Western Institutional Review Board (IRB protocol No. 20152817).

Additional information is provided in the Supplementary Materials and Methods, available at https://doi.org/10.1016/j.esmoop.2025.106048.

## Results

We obtained genomic data from 2372 HCC patients, who received diagnostic tumor profiling on the FoundationOne® platform, a hybrid capture approach that analyses >290 cancer relevant genes, tumor mutational burden (TMB), and microsatellite instability (MSI) status.

According to the information provided by the submitting physician, most samples were from the primary tumors (*n* = 1700, 71.7%) and 502 (21.2%) specimens were from metastatic sites. No information on the sampling site was provided for 170 (7.1%) cases. In metastatic lesions, GAs in *CTNNB1* and *MCL1* were significantly more prevalent than in profiles from primary sites (44.3% versus 32.7%, 7.1% versus 3.7%, respectively; *P* < 0.05; Supplementary Figure S1, available at https://doi.org/10.1016/j.esmoop.2025.106048).

**Figure 1:**
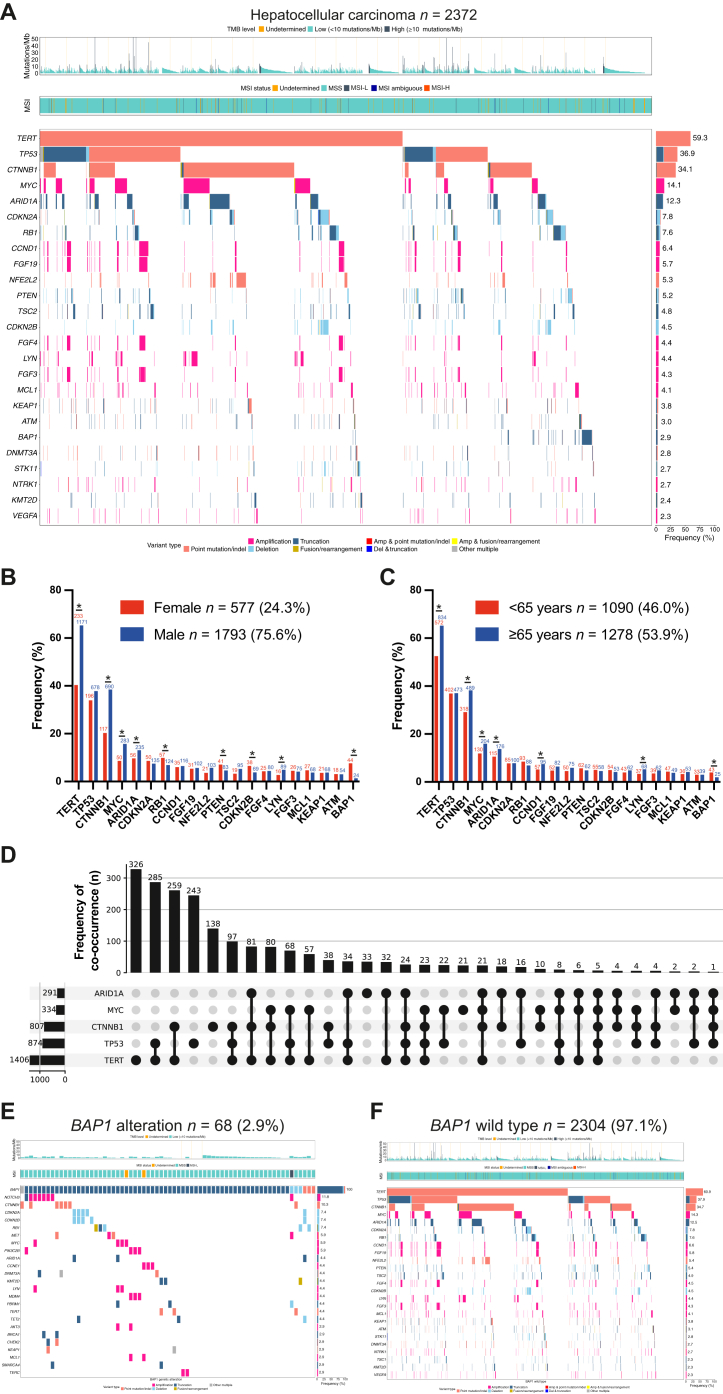
**Genomic landscape of a real-world HCC cohort.** (A) Tileplot displaying the top 25 GAs. Gene names (left) with corresponding alteration frequency (right). Color of tiles indicates the type of GA. Short variants, such as nonsense, frameshift, and splice alterations, as well as specific rearrangements that result in truncation of the gene product are summarized under ‘truncation’. TMB and MSI status are overlaid on top. (B, C) Frequency of top 20 GAs according to sex and age; absolute numbers are displayed above bars. (D) Upset plot of the five most common altered genes and their specific co-occurrences. Absolute numbers of GA for each single gene are indicated above; absolute numbers of specific co-occurrences are shown on the left side. (E, F) Tileplots depicting top 25 co-alterations in the HCC samples with *BAP1*-mutant and *BAP1*-wild-type samples. GA, genetic alteration; HCC, hepatocellular carcinoma; MSI, microsatellite instability; TMB, tumor mutational burden. ∗*P* < 0.05.

GAs were reported in 236 different genes in the whole cohort comprising 3024 different mutation types and loci including short variants (SVs), copy number alterations (CNAs), and rearrangements (REs). In total, 8519 mutations were reported in 2372 patients. Fifty-six genes were identified as recurrently altered in at least 1% of the total cohort (Supplementary Table S1, available at https://doi.org/10.1016/j.esmoop.2025.106048), the 10 most frequent being *TERT* (59.3%), *TP53* (36.9%), *CTNNB1* (34.1%), *MYC* (14.1%), *ARID1A* (12.3%), *CDKN2A* (7.8%), *RB1* (7.6%), *CCND1* (6.4%), *FGF19* (5.7%), and *NFE2L2* (5.3%) (Figure 1A).

Patients with *TERT* mutations harbored the C228T hotspot mutation in 95.4% of cases, and the C250T variant in 4.5%. Further highly recurrent hotspot mutations include *MUTYH G382D* (50.0% of *MUTYH*-mutant cases), *CHEK2 I157T* (37.8% of *CHEK2*-mutant cases), and *PIK3CA H1047R* (21.1% of *PIK3CA*-mutant cases). Of the 51 known/likely pathogenic *MUTYH* SVs identified, 49 were considered germline, according to a computational approach identified by modeling the alteration’s allele frequency, taking into account the tumor content, tumor ploidy, and the local copy number.^6^ The second gene with a significant number of germline variants was *CHEK2*: somatic versus germline status could be determined in 32/45 *CHEK2*-altered cases, of which 4 were considered somatic and 28 germline.

Mutations in *TP53*, the second most frequently altered gene, were more dispersed and located mostly in the DNA binding domain, with *R249S* being the most common variant (5.6%). The predominantly affected residues were R249, R273, R248, S125, and R337, but the majority (72.6%) of specific GAs occurred only four times or fewer in the full cohort (Supplementary Figure S2 and Table S2, available at https://doi.org/10.1016/j.esmoop.2025.106048). Mutations in *CTNNB1* were frequently located at the D32 residue [missense mutations to A/G/H/N/V/Y (17.2%)], followed by T41A (10.0%), S45P (9.1%), and S33C (5.4%).

Amplifications were recurrently observed for *MYC*, *CCND1*, and *FGF3/4/19*, while *CDKN2A/B*, *PTEN*, and *RB1* were often deleted (Figure 1, Supplementary Figure S3 and Table S3, available at https://doi.org/10.1016/j.esmoop.2025.106048). REs were rare in the cohort (Supplementary Table S4, available at https://doi.org/10.1016/j.esmoop.2025.106048).

Of note, previous studies reported *AXIN1* alterations in 10%-15% of cases,^7^, ^8^, ^9^ contrary to the strikingly low rate of *AXIN1* alterations in the current cohort.^10^ Therefore, the functional status of *AXIN1* VUS was manually re-annotated by an expert in molecular pathology. As a consequence, 143 of the 199 VUS cases were reclassified, resulting in a total of 153 pathogenic *AXIN1* alterations (6.5%) across all cases, making it the eighth most common alteration (Supplementary Table S5, available at https://doi.org/10.1016/j.esmoop.2025.106048). However, this prevalence remains lower than previously reported. Aside from *AXIN1*, five CNAs that occurred in >10 patients—*PRKDC* amplification (62/2372, 2.6%), *DDR2* amplification (149/2372, 6.3%), *MEF2B* amplification (24/2372, 1.0%), *RAC1* amplification (13/2372, 0.6%), and *PDK1* amplification (11/2372, 0.5%)—were classified as likely oncogenic based on the OncoKB assessment, whereas based on the Foundation Medicine (FMI) algorithm, the evidence was deemed insufficient to classify these CNAs as pathogenic.

To explore potential calendar-period or technological effects on variant detection, we compared the prevalence of alterations identified using baitsets corresponding to FoundationOne® (used before 27 August 2020) and the more recent F1CDx (Supplementary Table S6, available at https://doi.org/10.1016/j.esmoop.2025.106048). Overall, prevalence estimates were largely concordant; notably, improved detection of *TERT* and *MYC* alterations in the newer baitsets (54.0% versus 62.3% and 10.7% versus 16.0%; *P* < 0.05) may be attributable to increased baiting of the *TERT* promoter region and advances in copy-number calling.

In line with the reported demographic distribution, the cohort included significantly more male than female patients (75.6% versus 24.3%), with some distinct differences in the mutational profiles: GAs in *TERT*, *CTNNB1*, *MYC*, *ARID1A*, and *LYN* were more common in male than in female patients (male versus female: 65.3% versus 40.4%, 38.5% versus 20.3%, 15.8% versus 8.7%, 13.1% versus 9.7%, 5.0% versus 2.8%, respectively; *P* < 0.05), whereas in female patients GAs in tumor suppressor genes such as *RB1*, *PTEN*, and *CDKN2B* were more frequent (male versus female: 6.9% versus 9.9%, 4.6% versus 7.1%, 3.8% versus 6.6%, respectively; *P* < 0.05) (Figure 1B). Notably, *BAP1* alterations occurred nearly exclusively in female patients (1.3% versus 7.6%; *P* < 0.05), reflective of a previously reported higher *BAP1* alteration rate in female intrahepatic CCA (iCCA) patients.^11^

Our cohort was well balanced between patients <65 years (46.0%) and ≥65 years (53.9%) (Figure 1C). Median age was slightly lower in female patients (64 ± 17 years versus 66 ± 13 years). Younger patients were significantly more likely to harbor alterations in *BAP1* (3.9% versus 2.0%; *P* < 0.05), whereas alterations in *TERT*, *CTNNB1*, *MYC*, *ARID1A*, *CCND1*, and *LYN* were more prevalent in older patients (52.5% versus 65.3%, 29.2% versus 38.3%, 11.9% versus 16.0%, 10.6% versus 13.8%, 5.2% versus 7.4%, 3.4% versus 5.3%, respectively; *P* < 0.05) (Figure 1C).

### Co-occurrences and defining subtypes

Recurrent CNAs affecting genes in close chromosomal proximity are often related to focal or even arm-level chromosomal gains and losses, frequently occurring on chromosome (Chr) 1q or 8q in HCC.^12^ MYC is considered one of the most prominent oncogenic drivers in HCC encoded on Chr8q. In our cohort, in cases with *MYC* amplification, 7 samples were negative and 288 were positive for 8q gains. Among patients without *MYC* amplification, 959 were positive for 8q gains, while 667 were negative. This indicates that *MYC* amplification typically occurs alongside larger 8q gains, although significant 8q gains were also observed in many patients independent of *MYC* amplification. *LYN* amplifications, similarly located on Chr8q, co-occur in nearly one-third of *MYC*-amplified HCCs (Supplementary Table S7, available at https://doi.org/10.1016/j.esmoop.2025.106048).

Preclinical work has elegantly demonstrated the functional importance of co-occurring CNAs involving dosage gains and losses of adjacent genes in HCC that may be selected for during hepatocarcinogenesis.^13^ Consistent with this, our cohort frequently shows co-amplification of *FGF19*, *FGF3*, *FGF4*, and *CCND1*, reflective of their spatial proximity on Chr11, (Supplementary Figure S4 and Table S7, available at https://doi.org/10.1016/j.esmoop.2025.106048) and FGF19 and CCND1 have been shown to promote hepatocarcinogenesis in preclinical models.^14^

Low rates of co-occurrence may indicate functional disadvantage, redundancy, or distinct routes of malignant transformation (Supplementary Table S8, available at https://doi.org/10.1016/j.esmoop.2025.106048). This is, for instance, reflected by the rare co-occurrence of *APC* GAs in *CTNNB1*-altered HCC, potentially related to a convergence on WNT signaling, and in line with the mutual exclusivity of *APC* GAs in the presence of *RNF43* mutations, as an alternate source of WNT activation, in colorectal cancer.^15^ Further, HCCs with *FGF19* and *CCND1* amplifications had low rates of *CTNNB1* alterations. Although the previously reported observation that *TP53* and *CTNNB1* GAs rarely co-occur was formally confirmed in our cohort (Supplementary Table S8, available at https://doi.org/10.1016/j.esmoop.2025.106048),^8^ 24.3% of *CTNNB1*-altered cases harbored GAs in *TP53*, contrasting with 43.4% in the *CTNNB1*-wild-type (WT) cohort (Figure 1D).

Interestingly, although only 465 HCC patients (19.6%) within our cohort were triple WT for the recurrent HCC genes *TERT*, *TP53*, and *CTNNB1*, they accounted for 86.8% (*n* = 59) of the *BAP1*-mutated cases. Specifically, none of the 875 *TP53*-altered cases and only 3 of 1406 *TERT*-altered as well as 7 out of 808 *CTNNB1*-altered HCCs harbored a *BAP1* co-alteration. The presence of *BAP1* alterations has been associated with fibrolamellar features in HCC^16^ and strongly correlates with the presence of *FGFR2* fusions in iCCA.^11^ Together with the distinct co-mutational landscape (Figure 1E and F), these findings indicate that *BAP1*-mutant liver cancer exhibits characteristic molecular and clinical features. Overall, the observed co-mutation patterns may reflect divergent pathways of hepatocarcinogenesis and suggest the existence of biologically and clinically distinct HCC subgroups whose clinical characteristics and therapeutic relevance warrant further investigation.

### Combined hepatocellular carcinoma–cholangiocarcinoma

**Figure 2:**
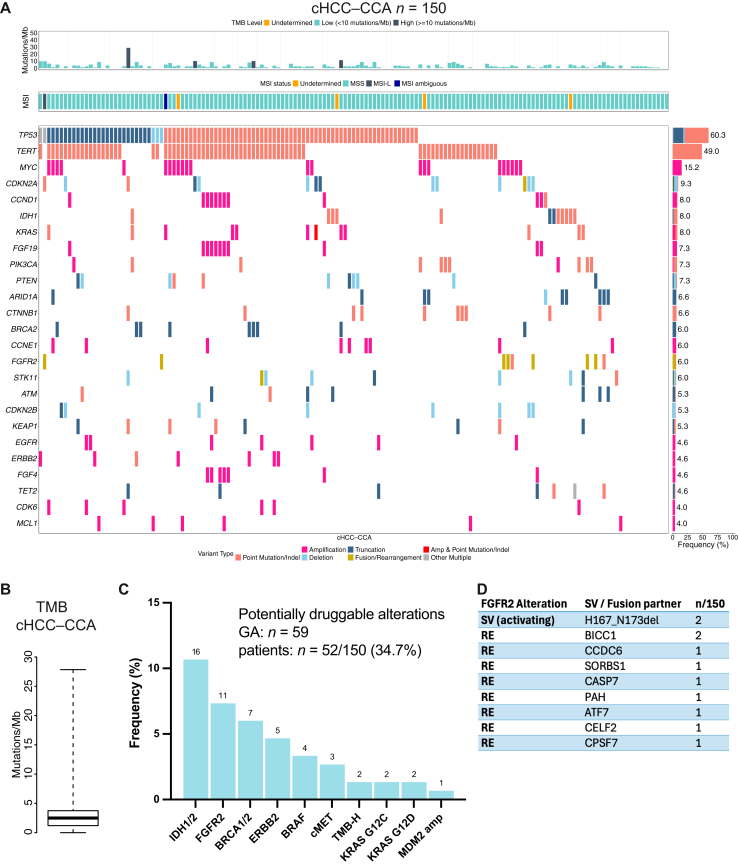
**GAs in cHCC–CCA.** (A) Tileplot depicting the top 25 GAs in cHCC–CCA. (B) Boxplot of the TMB in the cHCC–CCA cohort (median ± IQR). (C) Frequency of selected actionable alterations in cHCC–CCA with absolute numbers indicated above. (D) *FGFR* GAs in the cHCC–CCA cohort. For REs, fusion partners are indicated. cHCC–CCA, combined hepatocellular carcinoma–cholangiocarcinoma; GA, genomic alteration; IQR, interquartile range; MSI, microsatellite instability; MSI-L, MSI-low; RE, rearrangement; SV, short variant; TMB, tumor mutational burden.

cHCC–CCA is a rare cancer entity accounting for 1%–5% of primary liver malignancies.^17^ Similar to HCC, *TERT* promoter alterations and *TP53* GAs were the most common GAs across 150 cHCC–CCA cases (Figure 2A), but *TERT* promoter alterations were slightly less frequent (50.0% versus 59.3%) and *TP53* GAs were more common (60.0% versus 36.9%), compared with HCC (Supplementary Tables S9 and S10, available at https://doi.org/10.1016/j.esmoop.2025.106048). Notably, *CTNNB1* GAs were present in <7% of mixed cHCC–CCA tumors, compared with >34% in the HCC cohort. Median TMB was 2.5 ± 2.5 mutations/Mb (mut/Mb), and four cases were considered TMB-high (TMB-H; 2.7%) (Figure 2B). The spectrum of actionable alterations in the cHCC–CCA cohort was reminiscent of CCA, and actionable alterations, including *IDH1* mutations and *FGFR2* fusions, were detected in nearly 35% of cases, strongly advocating for patients with mixed cHCC–CCA to undergo broad molecular profiling, as recommended for patients with CCA (Figure 2C and D).

### Genetic ancestry-associated mutational spectrum in HCC

**Figure 3:**
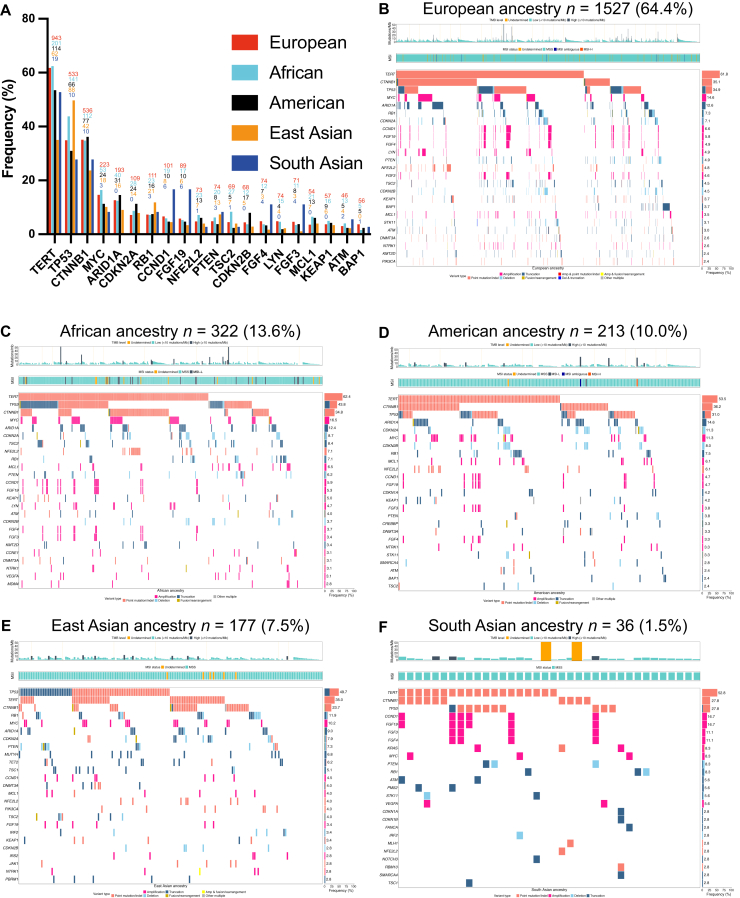
**GAs according to genetic ancestry.** (A) Frequency of the top 20 GAs according to genetic ancestry; absolute numbers are displayed above bars. (B-F) Corresponding tileplots depicting the top 25 genes (left) and their respective alteration frequencies (right). GA, genomic alteration; MSI, microsatellite instability; MSI-H, MSI-high; MSI-L, MSI-low; MSS, microsatellite stable; TMB, tumor mutational burden.

Next, we analyzed the mutational spectrum according to the five genomic ancestral superpopulations: in the FoundationCORE database, most patients were of European ancestry (*n* = 1527, 64.4%), followed by African (*n* = 322, 13.6%), American (*n* = 210, 10.0%), East Asian (*n* = 177, 7.5%), and South Asian (*n* = 36, 1.5%) ancestries (Figure 3). The ratio between male and female patients was similar across all ancestries (Supplementary Table S11, available at https://doi.org/10.1016/j.esmoop.2025.106048). Regarding age, patients of African (male: 64 ± 9 years; female: 62 ± 13 years) and East Asian (male: 62 ± 20.5 years; female: 66 ± 27 years) ancestries were often <65 years, while European (male: 67 ± 12 years; female: 65 ± 16.25 years) and South Asian patients (male: 66 ± 10.5 years; female: 58.5 ± 27.25 years) were more frequently in the ≥65-year age group. Patients of American ancestry (male: 63 ± 16 years; female: 67 ± 15.25 years) were more evenly distributed between the age groups.

Patients of East Asian genomic ancestry harbored fewer *TERT* and *CTNNB1* mutations, but had a higher rate of *TP53* GAs and a higher proportion of TMB-H (6.2%) tumors. This unique profile is likely associated with the prevalence of hepatitis B virus (HBV)-associated HCC in East Asian cohorts^18^ (Supplementary Figure S5, available at https://doi.org/10.1016/j.esmoop.2025.106048) and in line with the observation that, in HBV-associated HCC, TERT transcription activation may occur through viral intergration and not mutation.^19^ For *TP53*, an association between aflatoxin B1 exposure and the hotspot mutation at *R249S* has been described.^19^, ^20^, ^21^, ^22^ In the current dataset, the *R249S* mutation was detected in 51 patients (2.2% in the full cohort), but it accounted for 18.2% and 20.0% of *TP53* mutations in the East Asian and South Asian cohorts, respectively (Supplementary Tables S2 and S12, available at https://doi.org/10.1016/j.esmoop.2025.106048).

African patients exhibited a significantly higher prevalence of *TP53* alterations compared with American and European patients (43.8% versus 31.0% and 34.9%, respectively; *P* < 0.05). The frequency of *TSC2* alterations was also elevated in African patients, with a significant difference relative to American patients (8.4% versus 2.4%; *P* < 0.05).

No major differences were observed in the other top 20 recurrent HCC genes between the genomic ancestral populations, with the exception that *BAP1* alterations were not detected in patients of East Asian ancestry. Comparative assessments including the South Asian cohort should be made only with caution due to the small sample size.

### Tumor mutational burden

TMB is frequently regarded as a surrogate marker for neoantigen load and response to checkpoint inhibitors, but TMB-H cannot be considered a universal positive predictive biomarker across all solid tumor types.^23^

**Figure 4:**
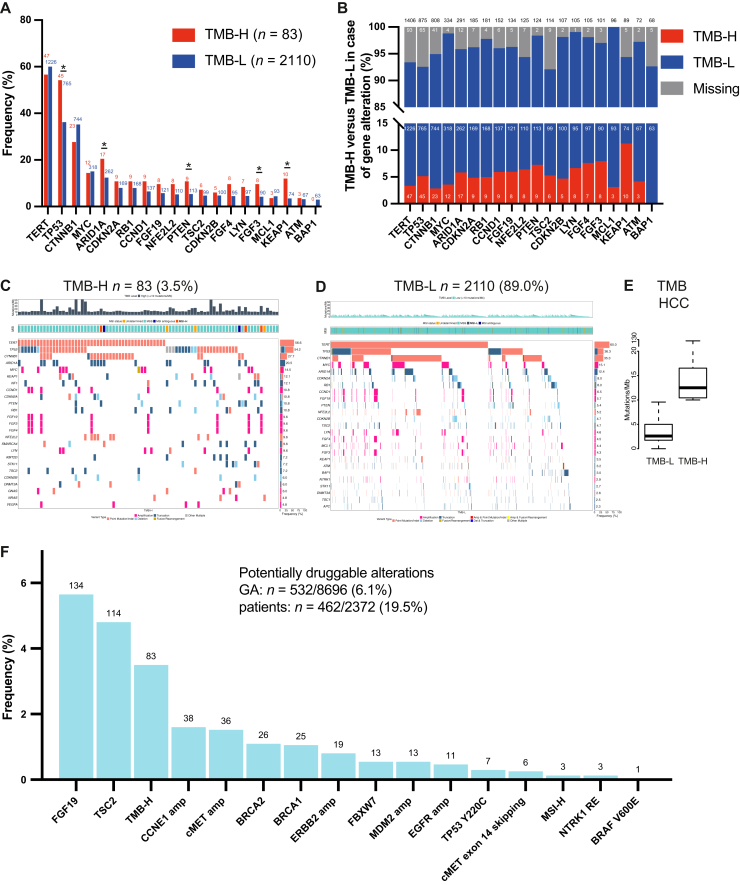
**Genomic profiles in TMB-H and TMB-L HCC patients and potentially actionable alterations.** (A) Frequency of the top 20 GAs according to TMB status; absolute numbers are displayed above bars. (B) TMB status according to genomic subgroups. Among tumors harboring the indicated GA, the fraction of tumors categorized as TMB-H is displayed in red, TMB-L in blue, and absolute numbers are shown within bars (total sum above bars). (C, D) Tileplots of the top 25 GAs according to TMB status. (E) Average TMB in the TMB-L versus TMB-H cohort (median ± IQR). (F) Frequency of selected potentially actionable alterations in HCC. Numbers above bars indicate absolute counts for each GA; *y*-axis depicts frequency. GA, genomic alteration; HCC, hepatocellular carcinoma; IQR, interquartile range; TMB, tumor mutational burden; TMB-H, TMB-high; TMB-L, TMB-low. ∗*P* < 0.05.

The median TMB in the HCC FoundationCORE database was 3.5 mut/Mb, indicating that HCC is a tumor type with an overall low mutational frequency. Only 83 (3.5%) samples were classified as TMB-H in this analysis, based on a cut-off of 10 mut/Mb. Median TMB was 3.5 mut/Mb in the TMB-low (TMB-L) group and 12.5 mut/Mb in the TMB-H group (Figure 4E).

As expected, we observed a higher number of GAs in TMB-H versus TMB-L tumors (Figure 4C). TMB-H was enriched for GAs in *TP53*, *ARID1A*, *PTEN*, *FGF3*, and *KEAP1* (54.2% versus 36.3%, 20.5% versus 12.4%, 10.8% versus 5.4%, 9.6% versus 4.3%, 12.0% versus 3.5%, respectively; *P* < 0.05) (Figure 4A, C, and D). Apart from the rare event of an MSI tumor [only *n* = 3 (0.1%) within the total cohort, 3/3 TMB-H], the presence of a *KEAP1* GA was associated with TMB-H status [11.2%, odds ratio (OR) 3.8, 95% confidence interval (CI) 1.9-7.6, *P* < 0.05, Figure 4B]. Also, patients with alterations in *FGF3* (7.9%, OR 2.4, 95% CI 1.1-5.1, *P* < 0.05) or *PTEN* (7.3%, OR 2.1, 95% CI 1.1-4.3, *P* < 0.05) were more likely to be TMB-H.

### Potentially actionable GAs in HCC patients

In contrast to biliary cancers, HCCs are considered notoriously ‘undruggable’ malignancies. At this point, no concepts targeting key GAs, such as those affecting the *TERT* promoter or *CTNNB1*, have been implemented in routine clinical practice, but the field of targeted treatments for solid malignancies is evolving.

In our cohort, 532 of 8696 reported GAs (6.1%) were classified as potentially actionable, which were detected in 462 patients (19.5%) (Figure 4F). ‘Classical’ oncogenic drivers, including *BRAF*, *ERBB2*, or oncogenic fusions, were overall rare in the cohort (Supplementary Tables S4 and S13, available at https://doi.org/10.1016/j.esmoop.2025.106048).

The extended mutational landscape of HCC is, however, broad, and contains GAs that may serve as predictive markers to prioritize alternative treatments or that might be amenable to targeted (combination) strategies, especially in later lines of therapy. One of the evolving targets is FGF19, whose overexpression has been an inclusion criterion for treatment in a clinical phase II trial with the FGFR4 inhibitor irpagratinib (ABSK-011).^24^ Given the frequent association between overexpression and amplification, 5.7% of patients in our cohort might have qualified for the targeted treatment (Supplementary Figure S6, available at https://doi.org/10.1016/j.esmoop.2025.106048). Notably, lenvatinib is an established multi-TKI with FGFR activity,^25^ and prioritization might be considered. Another important target of lenvatinib and other multi-TKIs, including sorafenib, is the vascular endothelial growth factor (VEGF) receptor. Amplification of its ligand VEGF-A has been suggested to serve as a predictive biomarker for sorafenib or lenvatinib in HCC^26^ and was reported in 52 cases (2.1%) in our HCC cohort. Epidermal growth factor receptor (EGFR/ERBB1) belongs to the ERBB receptor tyrosine kinase superfamily. While EGFR can be a therapeutic target, its activation is a recurrent mechanism of resistance across different cancer types under TKI treatment. In HCC, *EGFR* amplifications (*n* = 11 in our cohort) have been shown to convey resistance to lenvatinib, with a potential for co-targeting strategies.^27^ In this regard, in a small prospective exploratory study, the addition of gefitinib in lenvatinib-resistant HCC patients with high-level expression of EGFR achieved disease control in 70% of patients.^28^

Despite disappointing results in an all-comer population, a *post hoc* analysis from the EVOLVE trial,^29^ flanked by additional case-report level evidence, suggests the efficacy of mechanistic target of rapamycin (mTOR) inhibition in *TSC2*-mutant HCC.^30^ In our cohort, 114 patients (4.8%) harbored *TSC2* alterations, and *PTEN* alterations were detected in 124 patients (5.2%), converging on the phosphatidylinositol 3-kinase/mTOR pathway.

*CCNE1* amplifications (occurring in 1.6% of the HCC cohort) as well as *FBXW7* mutations (occurring in 0.5% of the HCC cohort) are considered biomarkers for clinical trial-grade PKMYT1 inhibitors such as lunresertib.^31^ Furthermore, GAs affecting *BRCA1/2*, reported in 51 patients (2.6%), are mechanistically linked to DNA damage repair deficiency and serve as positive predictors for poly (ADP-ribose) polymerase (PARP) inhibitor- or platinum-based therapy across different cancer entities. Corresponding data in HCC are thus far lacking.^32^^,^^33^

As precision oncology is evolving, tumor suppressor genes are shifting into the focus. *TP53* as a master tumor suppressor is tightly regulated, with MDM2 acting as one of its main inhibitors. Recent developments have led to early clinical trials addressing MDM2 inhibition in the context of solid *MDM2*-amplified malignancies.^34^ Compared with other cancer entities, the frequency of *MDM2* amplifications is low in HCC (*n* = 13, 0.5% in our cohort), indicating this strategy may only cater to a minority of patients. An alternative approach is to directly target mutant *TP53*. However, few strategies have been translated into clinical trials thus far and may only be effective in the presence of highly selected *TP53* alterations. For instance, the p53-reactivating small molecule rezatapopt exclusively binds to *TP53 Y220C* (*n* = 7 patients, 0.3% in our cohort).^35^ In summary, it has to be acknowledged that precision oncology concepts are not implemented in clinical practice, and that targets established in other solid tumor entities are overall rare in HCC.

### HBV-positive cases

In 177 samples, sequencing reads mapping to the HBV genome were detected, indicative of positive viral status (Supplementary Figure S5, available at https://doi.org/10.1016/j.esmoop.2025.106048). Of note, not all hepatocytes uniformly harbor HBV DNA even in the setting of chronic HBV infection. Therefore, the absence of viral sequencing reads in the NGS analysis cannot fully exclude underlying HBV infection. High rates of HBV-related HCCs are reported from Asia, and while the overall rate of positivity was only 7.5% in our cohort, the relative percentage was highest in patients of East Asian ancestry (55.93%, 99/177). HBV-positive HCC patients were more likely to be younger (<65 years: 71.2%, 126/177 versus ≥65 years: 28.8%, 51/177; *P* < 0.05).

HBV-positive and -negative HCCs exhibited distinct differences in their respective mutational profiles: most notably, and in agreement with previous reports, the rate of *TP53* alterations was higher in HBV-positive specimens (57.1% versus 35.3%; *P* < 0.05) (Supplementary Figure S6A, available at https://doi.org/10.1016/j.esmoop.2025.106048), whereas two classical drivers in hepatocarcinogenesis, *TERT* promoter alterations (32.8% versus 61.4%) and *CTNNB1* GAs (23.7% versus 34.9%), occurred less frequently (Supplementary Table S14, available at https://doi.org/10.1016/j.esmoop.2025.106048). Further, GAs in *RB*, *PTEN*, and *TSC2* were enriched in HBV-positive versus -negative cases.

### Diagnostic re-assessment based on genomic profiles

A total of 117 cases with an initial diagnosis of HCC who harbored 120 GAs frequently associated with iCCA underwent histopathological re-evaluation by a trained pathologist, including 12 cases with *FGFR2* REs, 29 cases with *IDH1* SVs, 7 cases with *IDH2* SVs, 6 cases with *BRAF*^*V600E*^ mutations, 37 cases with *KRAS* SVs, and 29 cases with *ERBB2* CNAs (Table 1). Three cases harbored more than one index alteration. The initial HCC diagnosis was confirmed in only 48 cases (41%), whereas re-assessment resulted in a change of diagnosis to iCCA (*n* = 23 cases), to gall-bladder carcinoma (*n* = 1), or to cHCC–CCA (*n* = 13 cases). In 32 cases no definite diagnosis could be established based on the available material. This observation exemplifies how histopathological re-assessment triggered by genomic analysis can have diagnostic and therapeutic implications for patients.

**Table 1:** Diagnostic re-evaluation based on selected genomic alterations

Gene	GAs that prompted re-assessment (*n*)^a^	Confirmation of diagnosis after re-evaluation (*n*)	Reclassification of initial HCC diagnosis (*n*)^b^
*FGFR2*^*RE*^	12	1	11
*IDH1*^*SV*^	29	6	23
*IDH2*^*SV*^	7	1	6
*ERBB2*^*amp*^	29	19	10
*BRAF*^*V600E*^	6	1	5
*KRAS*^*SV*^	37	20	17
Sum of GAs	120	48	72

cHCC–CCA, combined hepatocellular carcinoma–cholangiocarcinoma; GA, genomic alteration; HCC, hepatocellular carcinoma; iCCA, intrahepatic cholangiocarcinoma.

a Three patients had more than one of the respective GAs. As a result, 117 patients harbored 120 GAs.

b Among 69 patients with 72 GAs, reclassification led to 23 patients with iCCA, 1 with gall-bladder carcinoma, 13 with cHCC–CCA, and 32 patients for whom no definitive diagnosis could be established.

## Discussion

This study offers a comprehensive genomic characterization of 2372 HCC patients profiled through a certified hybrid capture NGS platform. Leveraging real-world NGS data, we identified 8696 GAs across 236 genes, revealing the mutational heterogeneity of HCC and enabling comparisons across age, sex, ancestry, and viral status.

Consistent with prior studies, the most commonly altered genes included *TERT*, *TP53*, and *CTNNB1*, followed by *MYC*, *ARID1A*, *CDKN2A*, *RB1*, *CCND1*, *FGF19*, and *NFE2L2*. These alterations reflect core oncogenic pathways in HCC, such as telomerase reactivation, WNT signaling, and cell cycle deregulation. A subset of these alterations co-occurred frequently, suggesting cooperative oncogenic mechanisms, while others, such as *TP53* and *CTNNB1*, occurred at rates lower than expected.

Sex-based stratification revealed that male patients were more likely to harbor alterations in canonical oncogenic drivers such as *TERT*, *CTNNB1*, *MYC*, and *ARID1A*. In contrast, female patients exhibited higher rates of *RB1*, *PTEN*, *CDKN2B*, and, notably, *BAP1* mutations—the latter one being nearly six-fold more common in females than in males. These sex-specific mutational patterns may reflect both biological and environmental influences. Men have consistently higher HCC incidence worldwide, attributed in part to higher exposure to established risk factors such as alcohol, smoking, and chronic viral hepatitis. However, hormonal influences are also likely contributors.^36^ These observations support the need for sex-aware therapeutic approaches and the potential exploration of hormone-related targets in HCC subtypes.

Similarly, age-based analyses revealed distinct GA profiles. Patients ≥65 years of age were more likely to have *TERT*, *CTNNB1*, *MYC*, and *CCND1* alterations, while *BAP1* mutations were more frequent in younger individuals. Of note, with the median age of female patients being lower than that of male patients, this difference may in part be influenced by sex bias, among others.

The dataset allowed for genomic ancestry-based comparisons, revealing that East Asian ancestry—although underrepresented—was associated with absence of *BAP1*, lower rates of *TERT* and *CTNNB1*, and higher rates of *TP53* GAs, particularly occurring at the aflatoxin and HBV-associated *R249S* hotspot. These findings align with the predominance of HBV-related HCC in East Asia, where TERT activation may be consequential of viral integration rather than promoter mutation.^37^^,^^38^ Consistently, HBV-sequence reads could be detected in 55% of East Asian patients, versus 7.5% in the full cohort. Overall, our analyses underline ancestral variation of genomic profiles, albeit an underrepresentation of non-Caucasian populations has to be recognized which may, in part, result from unequal access to advanced diagnostic tools within the health care systems.

The lack of established actionable driver alterations in HCC justifies the comparatively low rate of tumor genomic analyses carried out in routine clinical practice. Today, NGS results will impact therapeutic decision making likely only in a few cases and in advanced lines of therapy (e.g. *FGF19*, *TSC2*). Contrary to CCA, the majority of current novel developments in clinical trials for HCC rely on histology-based and not sequencing-based diagnostics, evidenced by FGF19-targeted therapeutics in early clinical trials, or antibodies, antibody–drug conjugates, or cellular therapeutics (e.g. chimeric antigen receptor T cells) directed at, among others, GLYPICAN 3. Notably, cHCC–CCA cases, featuring distinctively fewer *CTNNB1* and more *TP53* mutations, are characterized by a higher rate of GAs considered actionable today, highlighting the clinical relevance of molecular profiling in these rare but therapeutically relevant tumors.

Our data further highlight the utility of NGS in refining the diagnosis of liver tumors, advocating for critical evaluation of sequencing results: among samples initially designated as HCC, 117 harbored alterations more typical of iCCA or cHCC–CCA. Subsequent histopathological re-assessment resulted in a reclassification in >30%, underscoring the role of NGS as a complementary diagnostic tool, particularly in liver tumors with ambiguous or mixed features.

Several limitations warrant discussion. Firstly, the panel-based approach focuses on a curated selection of genes, and automated annotation pipelines may undercall functional variants, as exemplified by *AXIN1* variants in our cohort. Secondly, given the low numbers in the non-European ancestry cohort, any associations should be interpreted with caution. Further, while the cohort is large, it reflects a United States-based patient population with access to clinical testing, and thus does not represent the full global spectrum of HCC. Thirdly, information on the primary tumor and biopsy site was at the discretion of the submitting physician, and the possibility of misclassifications cannot be fully excluded. Finally, clinical parameters, including disease etiology or severity of underlying liver disease, are largely lacking, and therefore, no clear correlations can be drawn between clinical parameters and genomic subgroups.

Despite these limitations, our study provides one of the largest NGS-profiled HCC cohorts to date and reinforces the importance of integrated molecular diagnostics: The observed molecular diversity—shaped by sex, age, ancestry, and viral status—may inform future therapeutic development. As precision oncology advances, NGS profiling holds value not only for identifying therapeutic targets but also for enhancing diagnostic precision in liver cancer.
