# Supplementary material for: Mutational patterns and ancestry-linked profiles in a large hepatocellular carcinoma and combined hepatocellular–cholangiocarcinoma cohort
Source: ESMO Open. 2026 Jan 20;11(2):106048. doi: 10.1016/j.esmoop.2025.106048 (PMC12857332; doi:10.1016/j.esmoop.2025.106048)
Supplement: Supplementary Material [file mmc1.docx]

**Mutational Patterns and Ancestry-Linked Profiles in a Large Hepatocellular Carcinoma and Combined Hepatocellular-Cholangiocarcinoma Cohort**

**Christoph Gerdes*,** **Shruthi Rengarajan*, Karthikeyan Murugesan, Jeffrey S Ross, Stephan Bartels, Arndt Vogel^†^ and Anna Saborowski^†^**

* equal contribution

† equal contribution

# **Supplementary Material and Methods**

FoundationCORE database - processing

All specimens submitted for sequencing featured a minimum of 20% tumor cell nuclear area and yielded a minimum of 50ng of extracted DNA. Comprehensive genomic profiling was performed on hybrid-capture, adapter ligation-based libraries, to identify GAs (base substitutions, small insertions and deletions, copy number alterations (CNAs) and REs) in coding exons (F1CDx (867 patients): *N* = 309; FoundationOne: *N* = 395) and select introns of cancer-associated genes (F1CDx (1,505 patients): *N* = 36; FoundationOne: *N* = 31). DNA-sequencing was performed on the Illumina HiSeq 4000 or NovaSeq 6000 platforms to a median sequencing depth of 896x (IQR 771x-1007x). Sequencing data were analyzed through a highly customized bioinformatic pipeline which detects base substitutions, short insertions and deletions (indels), copy number alterations (amplifications and homozygous deletions), and rearrangements.

For CNAs, a threshold of at least 6 for an amplification call adjusted for ploidy was used, with *ERBB2* as an exception with a minimum *ERBB2* call of 5. TMB was calculated as the number of non-driver somatic coding mutations per megabase (mutations/mb) of genome sequenced. TMB-high (TMB-H) was defined as 10 mutations/mb or higher. MSI status was determined by analyzing 114 intronic homopolymer repeat loci for length variability and MSI-high (MSI-H) was defined as described previously ^1,2^.

Nomenclature of variant types reported by F1CDx

Point Mutation/Indel: Functional short variant (SV) of the type missense or nonframeshift or nonstart or nonstop or promoter gene alteration. Truncation: Functional short variant of the type nonsense or frameshift or splice gene alteration. CNA, amplification: Functional gene copy number amplification; CNA, deletion: Functional gene copy number deletion. *FGFR2*RE: *FGFR2*RE is predicted to be a fusion when the breakpoint is within the *FGFR2* intron 17/exon 18 hotspot and the gene partner is known in the literature or is a novel partner that is predicted to be in-frame with *FGFR2*; alternatively, other *FGFR2* rearrangements not meeting the definition of fusions consist of rearrangements where the breakpoint is within the *FGFR2* intron 17/exon 18 hotspots but the partner gene is out-of-frame or out-of-strand with exon 17 of *FGFR2*. Alternatively, the downstream end of the breakpoint in a rearrangement may be in an intergenic region (the latter designated as *FGFR2*-NA).

Software and statistical analyses

For each genomic sub-cohort analyzed, the prevalence of other gene alterations and the co-occurrence and mutual exclusivity of these gene alterations were studied. For categorical variables, the Fisher's exact test was used to assess the odds ratio and *p*-value, and the *p*-values were corrected for multiple hypothesis testing using the Benjamini-Hochberg method. For continuous variables the Wilcoxon Rank Sum test was used to assess associations. The oncoprints depict the top 25 most prevalent genes. Gene associations with a Fisher's exact *p*-value of association < 0.05 are reported. For data management and statistical analysis R Software (R Foundation for Statistical Computing, Vienna, Austria, v.4.0.3 or higher), Python v.2.7.16 (Python Software Foundation, Wilmington, DE, US) and GraphPad Prism 10.2.3 (347) (GraphPad Software, California, USA) was used.

Genetic ancestry

Ancestry was inferred using a single nucleotide polymorphism (SNP)-based approach as described before to distinguish between the 5 ancestral superpopulations in the 1,000 Genomes Project reference dataset: African, admixed American (a proxy for Hispanic), East Asian, European, and South Asian ^3,4^.

Viral detection

Sequencing reads left unmapped to the human reference genome (hg19) are de novo assembled by Velvet ^5^, and the assembled contigs are competitively aligned by BLASTn to the National Center for Biotechnology Information (NCBI) database of more than 3 million known viral nucleotide sequences to detect oncoviruses, including the hepatitis B virus (HBV). A positive viral status is determined by contigs at least 80 nucleotides in length and with at least 97% identity to the BLAST sequence.

*AXIN1* variant prediction

For the *AXIN1* gene alterations an additional manual annotation of the functional effect of the single variant was performed. Evaluation of variant annotation was performed with the ANNOVAR software and database tools ^6^. Different *in silico* prediction tools and scores were taken into account (e.g. SIFT, PolyPhen2, CADD, FATHMM, MutationTaster, MutationAssesor). For nonsense, splice site, and frameshift mutations with a premature stop codon a loss-of-function pathogenicity for the tumor suppressor *AXIN1* was assumed.

Reevaluation of uncertain genetic alterations

For functional assessment, several bioinformatic and database-driven tools were consulted. GeneBe (https://genebe.net/) was used to retrieve all available in silico prediction scores, and VarSome (https://varsome.com/) to integrate ClinVar entries, ACMG classification, and additional public datasets. The oncogenic relevance of each CNA was then evaluated using the Cancer Knowledge Base (CKB, https://ckb.genomenon.com/gene/grid) and OncoKB (<https://www.oncokb.org/>) ^7,8^.

Assessment of Germline Status

FMI uses a computational approach to predict germline versus somatic status according to Sun et al. (PMID: 29415044). Briefly, in this method, the cancer specimen is sequenced to high depth (>500x) using MPS, in our implementation by a targeted clinical assay of 394 cancer-related genes and over 3,500 genome-wide SNPs. SGZ (somatic-germline-zygosity) leverages the precise measurement of the allele frequencies of variants of interest offered by deep sequencing and a statistical model of genome-wide copy number and tumor/normal admixture to characterize the mutational state of the variants.

Data sharing statement

Summary data that can be released are included in the article and its supplementary files. Patients were not consented for the release of individualized patient genomic sequencing data which contains potentially identifying or sensitive patient information. More information and mechanisms for data access can be obtained by contacting the corresponding author or the Foundation Medicine Data Governance Council at [data.governance.council@foundationmedicine.com](mailto:data.governance.council@foundationmedicine.com).

1. Chalmers ZR, Connelly CF, Fabrizio D, et al. Analysis of 100,000 human cancer genomes reveals the landscape of tumor mutational burden. *Genome Med*. Apr 19 2017;9(1):34. doi:10.1186/s13073-017-0424-2

2. Trabucco SE, Gowen K, Maund SL, et al. A Novel Next-Generation Sequencing Approach to Detecting Microsatellite Instability and Pan-Tumor Characterization of 1000 Microsatellite Instability-High Cases in 67,000 Patient Samples. *J Mol Diagn*. Nov 2019;21(6):1053-1066. doi:10.1016/j.jmoldx.2019.06.011

3. Mata DA, Rotenstein LS, Ramos MA, Jena AB. Disparities According to Genetic Ancestry in the Use of Precision Oncology Assays. *N Engl J Med*. Jan 19 2023;388(3):281-283. doi:10.1056/NEJMc2213457

4. Sivakumar S, Lee JK, Moore JA, et al. Comprehensive genomic profiling and treatment patterns across ancestries in advanced prostate cancer: a large-scale retrospective analysis. *Lancet Digit Health*. Jun 2023;5(6):e380-e389. doi:10.1016/s2589-7500(23)00053-5

5. Knepper TC, Montesion M, Russell JS, et al. The Genomic Landscape of Merkel Cell Carcinoma and Clinicogenomic Biomarkers of Response to Immune Checkpoint Inhibitor Therapy. *Clin Cancer Res*. Oct 1 2019;25(19):5961-5971. doi:10.1158/1078-0432.Ccr-18-4159

6. Wang K, Li M, Hakonarson H. ANNOVAR: functional annotation of genetic variants from high-throughput sequencing data. *Nucleic Acids Research*. 2010;38(16):e164-e164. doi:10.1093/nar/gkq603
7. Suehnholz SP, Nissan MH, Zhang H, et al. Quantifying the Expanding Landscape of Clinical Actionability for Patients with Cancer. Cancer Discovery. 2024;14(1):49-65. doi:10.1158/2159-8290.Cd-23-0467
8. Chakravarty D, Gao J, Phillips S, et al. OncoKB: A Precision Oncology Knowledge Base. JCO Precision Oncology. 2017;(1):1-16. doi:10.1200/po.17.00011
