# Supplementary material for: Mutational patterns and ancestry-linked profiles in a large hepatocellular carcinoma and combined hepatocellular–cholangiocarcinoma cohort
Source: ESMO Open. 2026 Jan 20;11(2):106048. doi: 10.1016/j.esmoop.2025.106048 (PMC12857332; doi:10.1016/j.esmoop.2025.106048)
Supplement: Supplementary Tables [file mmc2.docx]

**Mutational Patterns and Ancestry-Linked Profiles in a Large Hepatocellular Carcinoma and Combined Hepatocellular-Cholangiocarcinoma Cohort**

**Christoph Gerdes*,** **Shruthi Rengarajan*, Karthikeyan Murugesan, Jeffrey S Ross, Stephan Bartels, Arndt Vogel^†^ and Anna Saborowski^†^**

* equal contribution

† equal contribution

# **Supplementary Tables**

**Supplementary Table 1: Gene alterations in HCC patients.**

| **Gene** | **Incidence** | **Total Population** | **Frequency (%)** |
| --- | --- | --- | --- |
| *TERT* | 1406 | 2372 | 59.27% |
| *TP53* | 875 | 2372 | 36.89% |
| *CTNNB1* | 808 | 2372 | 34.06% |
| *MYC* | 334 | 2372 | 14.08% |
| *ARID1A* | 291 | 2372 | 12.27% |
| *CDKN2A* | 185 | 2372 | 7.80% |
| *RB1* | 181 | 2372 | 7.63% |
| *HBV* | 177 | 2372 | 7.46% |
| *CCND1* | 152 | 2372 | 6.41% |
| *FGF19* | 134 | 2372 | 5.65% |
| *NFE2L2* | 125 | 2372 | 5.27% |
| *PTEN* | 124 | 2372 | 5.23% |
| *TSC2* | 114 | 2372 | 4.81% |
| *CDKN2B* | 107 | 2372 | 4.51% |
| *LYN* | 105 | 2372 | 4.43% |
| *FGF4* | 105 | 2372 | 4.43% |
| *FGF3* | 101 | 2372 | 4.26% |
| *MCL1* | 96 | 2372 | 4.05% |
| *KEAP1* | 89 | 2372 | 3.75% |
| *ATM* | 72 | 2372 | 3.04% |
| *BAP1* | 68 | 2372 | 2.87% |
| *DNMT3A* | 66 | 2372 | 2.78% |
| *STK11* | 64 | 2372 | 2.70% |
| *NTRK1* | 63 | 2372 | 2.66% |
| *KMT2D* | 56 | 2372 | 2.36% |
| *VEGFA* | 54 | 2372 | 2.28% |
| *TSC1* | 54 | 2372 | 2.28% |
| *APC* | 52 | 2372 | 2.19% |
| *PIK3CA* | 52 | 2372 | 2.19% |
| *MUTYH* | 52 | 2372 | 2.19% |
| *TET2* | 49 | 2372 | 2.07% |
| *CDKN1A* | 48 | 2372 | 2.02% |
| *NF1* | 45 | 2372 | 1.90% |
| *CHEK2* | 45 | 2372 | 1.90% |
| *PBRM1* | 44 | 2372 | 1.85% |
| *MET* | 44 | 2372 | 1.85% |
| *KRAS* | 41 | 2372 | 1.73% |
| *CCNE1* | 39 | 2372 | 1.64% |
| *GNAS* | 38 | 2372 | 1.60% |
| *CDKN1B* | 37 | 2372 | 1.56% |
| *CREBBP* | 36 | 2372 | 1.52% |
| *PIK3C2B* | 34 | 2372 | 1.43% |
| *SMARCA4* | 34 | 2372 | 1.43% |
| *SETD2* | 32 | 2372 | 1.35% |
| *ERRFI1* | 31 | 2372 | 1.31% |
| *MDM4* | 31 | 2372 | 1.31% |
| *SF3B1* | 30 | 2372 | 1.26% |
| *FANCA* | 29 | 2372 | 1.22% |
| *HGF* | 28 | 2372 | 1.18% |
| *NOTCH3* | 28 | 2372 | 1.18% |
| *NOTCH2* | 28 | 2372 | 1.18% |
| *CCND3* | 27 | 2372 | 1.14% |
| *AKT3* | 27 | 2372 | 1.14% |
| *IRS2* | 26 | 2372 | 1.10% |
| *BRCA2* | 26 | 2372 | 1.10% |
| *BRCA1* | 25 | 2372 | 1.05% |
| *EGFR* | 24 | 2372 | 1.01% |
| *ASXL1* | 23 | 2372 | 0.97% |
| *KDM6A* | 22 | 2372 | 0.93% |
| *IRF2* | 22 | 2372 | 0.93% |
| *ZNF217* | 22 | 2372 | 0.93% |
| *ERBB2* | 21 | 2372 | 0.89% |
| *RICTOR* | 20 | 2372 | 0.84% |
| *CBL* | 20 | 2372 | 0.84% |
| *SMAD4* | 19 | 2372 | 0.80% |
| *FAS* | 19 | 2372 | 0.80% |
| *AKT2* | 18 | 2372 | 0.76% |
| *ATRX* | 18 | 2372 | 0.76% |
| *CDK6* | 18 | 2372 | 0.76% |
| *FLCN* | 18 | 2372 | 0.76% |
| *NF2* | 17 | 2372 | 0.72% |
| *NRAS* | 17 | 2372 | 0.72% |
| *RBM10* | 16 | 2372 | 0.67% |
| *ACVR1B* | 16 | 2372 | 0.67% |
| *ATR* | 16 | 2372 | 0.67% |
| *TERC* | 16 | 2372 | 0.67% |
| *PTPN11* | 15 | 2372 | 0.63% |
| *MAP2K4* | 15 | 2372 | 0.63% |
| *EP300* | 15 | 2372 | 0.63% |
| *JAK2* | 15 | 2372 | 0.63% |
| *AURKA* | 15 | 2372 | 0.63% |
| *FGF14* | 14 | 2372 | 0.59% |
| *PRKCI* | 14 | 2372 | 0.59% |
| *STAG2* | 14 | 2372 | 0.59% |
| *MDM2* | 13 | 2372 | 0.55% |
| *FBXW7* | 13 | 2372 | 0.55% |
| *BCORL1* | 13 | 2372 | 0.55% |
| *PIK3R1* | 13 | 2372 | 0.55% |
| *BRIP1* | 13 | 2372 | 0.55% |
| *PRKN* | 13 | 2372 | 0.55% |
| *IKBKE* | 13 | 2372 | 0.55% |
| *MEN1* | 13 | 2372 | 0.55% |
| *KEL* | 13 | 2372 | 0.55% |
| *CDK4* | 12 | 2372 | 0.51% |
| *ZNF703* | 12 | 2372 | 0.51% |
| *NOTCH1* | 12 | 2372 | 0.51% |
| *SPEN* | 12 | 2372 | 0.51% |
| *AXL* | 12 | 2372 | 0.51% |
| *BARD1* | 12 | 2372 | 0.51% |
| *FGFR1* | 12 | 2372 | 0.51% |
| *MSH6* | 11 | 2372 | 0.46% |
| *SMARCB1* | 11 | 2372 | 0.46% |
| *FGF10* | 11 | 2372 | 0.46% |
| *RET* | 11 | 2372 | 0.46% |
| *MTOR* | 11 | 2372 | 0.46% |
| *STAT3* | 11 | 2372 | 0.46% |
| *U2AF1* | 10 | 2372 | 0.42% |
| *CRKL* | 10 | 2372 | 0.42% |
| *BCOR* | 10 | 2372 | 0.42% |
| *INPP4B* | 10 | 2372 | 0.42% |
| *ARFRP1* | 10 | 2372 | 0.42% |
| *AMER1* | 10 | 2372 | 0.42% |
| *CDH1* | 10 | 2372 | 0.42% |
| *CCND2* | 10 | 2372 | 0.42% |
| *PRDM1* | 10 | 2372 | 0.42% |
| *FGF23* | 10 | 2372 | 0.42% |
| *FGFR3* | 10 | 2372 | 0.42% |
| *BRAF* | 10 | 2372 | 0.42% |
| *AXIN1* | 10 | 2372 | 0.42% |
| *JAK1* | 10 | 2372 | 0.42% |
| *EPHA3* | 10 | 2372 | 0.42% |
| *CD274* | 9 | 2372 | 0.38% |
| *PDCD1LG2* | 9 | 2372 | 0.38% |
| *MLH1* | 9 | 2372 | 0.38% |
| *PRKAR1A* | 9 | 2372 | 0.38% |
| *FGF6* | 9 | 2372 | 0.38% |
| *MYCL* | 9 | 2372 | 0.38% |
| *VHL* | 8 | 2372 | 0.34% |
| *EMSY* | 8 | 2372 | 0.34% |
| *HNF1A* | 8 | 2372 | 0.34% |
| *CDKN2C* | 8 | 2372 | 0.34% |
| *FGFR2* | 8 | 2372 | 0.34% |
| *CTCF* | 8 | 2372 | 0.34% |
| *RPTOR* | 8 | 2372 | 0.34% |
| *SDHA* | 8 | 2372 | 0.34% |
| *FANCC* | 8 | 2372 | 0.34% |
| *MAP3K1* | 8 | 2372 | 0.34% |
| *BCL2L1* | 8 | 2372 | 0.34% |
| *PALB2* | 8 | 2372 | 0.34% |
| *NFKBIA* | 7 | 2372 | 0.30% |
| *ERBB4* | 7 | 2372 | 0.30% |
| *BRD4* | 7 | 2372 | 0.30% |
| *RNF43* | 7 | 2372 | 0.30% |
| *MAP2K1* | 7 | 2372 | 0.30% |
| *JUN* | 7 | 2372 | 0.30% |
| *MRE11* | 7 | 2372 | 0.30% |
| *PDGFRA* | 7 | 2372 | 0.30% |
| *PTCH1* | 6 | 2372 | 0.25% |
| *WT1* | 6 | 2372 | 0.25% |
| *MSH2* | 6 | 2372 | 0.25% |
| *BCL2L2* | 6 | 2372 | 0.25% |
| *KDM5A* | 6 | 2372 | 0.25% |
| *IDH1* | 6 | 2372 | 0.25% |
| *CDC73* | 6 | 2372 | 0.25% |
| *ALK* | 6 | 2372 | 0.25% |
| *CEBPA* | 6 | 2372 | 0.25% |
| *SOX9* | 6 | 2372 | 0.25% |
| *GID4* | 6 | 2372 | 0.25% |
| *NKX2-1* | 5 | 2372 | 0.21% |
| *FANCL* | 5 | 2372 | 0.21% |
| *KDM5C* | 5 | 2372 | 0.21% |
| *AR* | 5 | 2372 | 0.21% |
| *PDGFRB* | 5 | 2372 | 0.21% |
| *ERBB3* | 5 | 2372 | 0.21% |
| *QKI* | 5 | 2372 | 0.21% |
| *FUBP1* | 5 | 2372 | 0.21% |
| *SRC* | 5 | 2372 | 0.21% |
| *KDR* | 5 | 2372 | 0.21% |
| *EPHB1* | 5 | 2372 | 0.21% |
| *KIT* | 5 | 2372 | 0.21% |
| *CDK12* | 5 | 2372 | 0.21% |
| *AKT1* | 4 | 2372 | 0.17% |
| *EZH2* | 4 | 2372 | 0.17% |
| *ROS1* | 4 | 2372 | 0.17% |
| *MYCN* | 4 | 2372 | 0.17% |
| *SMAD2* | 4 | 2372 | 0.17% |
| *CIC* | 4 | 2372 | 0.17% |
| *RAF1* | 4 | 2372 | 0.17% |
| *PMS2* | 4 | 2372 | 0.17% |
| *MITF* | 4 | 2372 | 0.17% |
| *MYB* | 3 | 2372 | 0.13% |
| *GATA6* | 3 | 2372 | 0.13% |
| *FH* | 3 | 2372 | 0.13% |
| *CSF1R* | 3 | 2372 | 0.13% |
| *GNA11* | 3 | 2372 | 0.13% |
| *CTNNA1* | 3 | 2372 | 0.13% |
| *NTRK3* | 3 | 2372 | 0.13% |
| *ABL1* | 3 | 2372 | 0.13% |
| *GRM3* | 3 | 2372 | 0.13% |
| *HSD3B1* | 3 | 2372 | 0.13% |
| *GATA4* | 3 | 2372 | 0.13% |
| *FGFR4* | 3 | 2372 | 0.13% |
| *IGF1R* | 2 | 2372 | 0.08% |
| *CARD11* | 2 | 2372 | 0.08% |
| *FANCG* | 2 | 2372 | 0.08% |
| *SDHC* | 2 | 2372 | 0.08% |
| *PIK3CB* | 2 | 2372 | 0.08% |
| *MYD88* | 2 | 2372 | 0.08% |
| *SUFU* | 2 | 2372 | 0.08% |
| *TBX3* | 2 | 2372 | 0.08% |
| *ETV6* | 2 | 2372 | 0.08% |
| *SDHB* | 2 | 2372 | 0.08% |
| *KMT2A* | 2 | 2372 | 0.08% |
| *TGFBR2* | 2 | 2372 | 0.08% |
| *PPP2R1A* | 2 | 2372 | 0.08% |
| *TNFRSF14* | 2 | 2372 | 0.08% |
| *CDK8* | 2 | 2372 | 0.08% |
| *BCL2* | 2 | 2372 | 0.08% |
| *DAXX* | 2 | 2372 | 0.08% |
| *NPM1* | 2 | 2372 | 0.08% |
| *GNAQ* | 2 | 2372 | 0.08% |
| *HRAS* | 2 | 2372 | 0.08% |
| *GABRA6* | 1 | 2372 | 0.04% |
| *SOCS1* | 1 | 2372 | 0.04% |
| *SOX2* | 1 | 2372 | 0.04% |
| *ERG* | 1 | 2372 | 0.04% |
| *DDR2* | 1 | 2372 | 0.04% |
| *CHEK1* | 1 | 2372 | 0.04% |
| *BTG1* | 1 | 2372 | 0.04% |
| *IDH2* | 1 | 2372 | 0.04% |
| *CUL3* | 1 | 2372 | 0.04% |
| *AURKB* | 1 | 2372 | 0.04% |
| *RAD51* | 1 | 2372 | 0.04% |
| *CBFB* | 1 | 2372 | 0.04% |
| *CD79A* | 1 | 2372 | 0.04% |
| *ARAF* | 1 | 2372 | 0.04% |
| *MAP2K2* | 1 | 2372 | 0.04% |
| *BTK* | 1 | 2372 | 0.04% |
| *SDHD* | 1 | 2372 | 0.04% |
| *POLE* | 1 | 2372 | 0.04% |
| *FLT1* | 1 | 2372 | 0.04% |
| *H3-3A* | 1 | 2372 | 0.04% |
| *DOT1L* | 1 | 2372 | 0.04% |
| *KLHL6* | 1 | 2372 | 0.04% |
| *PAX5* | 1 | 2372 | 0.04% |
| *MED12* | 1 | 2372 | 0.04% |
| *GNA13* | 1 | 2372 | 0.04% |

**Supplementary Table 2: TP53 alterations in HCC.**

| **Gene** | **Alteration Type** | **SV-proteinEffect/CN-ampOrDel/RE-partnerGene** | **Incidence** | **All *TP53* alterations** | **Frequency (%)** |
| --- | --- | --- | --- | --- | --- |
| *TP53* | SV | R249S | 51 | 915 | 5.57% |
| *TP53* | CN | deletion | 28 | 915 | 3.06% |
| *TP53* | SV | R175H | 11 | 915 | 1.20% |
| *TP53* | SV | R273C | 10 | 915 | 1.09% |
| *TP53* | SV | V157F | 9 | 915 | 0.98% |
| *TP53* | SV | R337L | 8 | 915 | 0.87% |
| *TP53* | SV | R282W | 8 | 915 | 0.87% |
| *TP53* | SV | H193R | 7 | 915 | 0.77% |
| *TP53* | RE | TP53 | 7 | 915 | 0.77% |
| *TP53* | SV | M246V | 7 | 915 | 0.77% |
| *TP53* | SV | G266V | 7 | 915 | 0.77% |
| *TP53* | SV | Y220C | 7 | 915 | 0.77% |
| *TP53* | SV | Y234C | 7 | 915 | 0.77% |
| *TP53* | SV | H179R | 6 | 915 | 0.66% |
| *TP53* | SV | R248Q | 6 | 915 | 0.66% |
| *TP53* | SV | Q192* | 6 | 915 | 0.66% |
| *TP53* | SV | R248W | 6 | 915 | 0.66% |
| *TP53* | SV | R273H | 6 | 915 | 0.66% |
| *TP53* | SV | R273L | 6 | 915 | 0.66% |
| *TP53* | SV | R110L | 6 | 915 | 0.66% |
| *TP53* | SV | Y163C | 6 | 915 | 0.66% |
| *TP53* | SV | E298* | 6 | 915 | 0.66% |
| *TP53* | SV | C135Y | 5 | 915 | 0.55% |
| *TP53* | SV | L111Q | 5 | 915 | 0.55% |
| *TP53* | SV | R158L | 5 | 915 | 0.55% |
| *TP53* | SV | R337C | 5 | 915 | 0.55% |
| *TP53* | SV | R342* | 5 | 915 | 0.55% |
| *TP53* | SV | M237I | 5 | 915 | 0.55% |
| *TP53* | SV | G334V | 4 | 915 | 0.44% |
| *TP53* | SV | R196* | 4 | 915 | 0.44% |
| *TP53* | SV | W146* | 4 | 915 | 0.44% |
| *TP53* | SV | C141Y | 4 | 915 | 0.44% |
| *TP53* | SV | P190L | 4 | 915 | 0.44% |
| *TP53* | SV | Y236C | 4 | 915 | 0.44% |
| *TP53* | SV | G266E | 4 | 915 | 0.44% |
| *TP53* | SV | A161T | 4 | 915 | 0.44% |
| *TP53* | SV | splice site 673-2A>T | 4 | 915 | 0.44% |
| *TP53* | SV | R213Q | 4 | 915 | 0.44% |
| *TP53* | SV | S240G | 4 | 915 | 0.44% |
| *TP53* | SV | R248L | 4 | 915 | 0.44% |
| *TP53* | SV | E204* | 4 | 915 | 0.44% |
| *TP53* | SV | R213* | 4 | 915 | 0.44% |
| *TP53* | SV | G245D | 4 | 915 | 0.44% |
| *TP53* | SV | splice site 375G>T | 4 | 915 | 0.44% |
| *TP53* | SV | S90fs*33 | 4 | 915 | 0.44% |
| *TP53* | SV | P153fs*28 | 4 | 915 | 0.44% |
| *TP53* | SV | S241C | 4 | 915 | 0.44% |
| *TP53* | SV | G245V | 4 | 915 | 0.44% |
| *TP53* | SV | R213L | 4 | 915 | 0.44% |
| *TP53* | SV | R248G | 3 | 915 | 0.33% |
| *TP53* | SV | Y163H | 3 | 915 | 0.33% |
| *TP53* | SV | F113V | 3 | 915 | 0.33% |
| *TP53* | SV | Q144* | 3 | 915 | 0.33% |
| *TP53* | SV | Y103* | 3 | 915 | 0.33% |
| *TP53* | SV | C275Y | 3 | 915 | 0.33% |
| *TP53* | SV | G105V | 3 | 915 | 0.33% |
| *TP53* | SV | E294* | 3 | 915 | 0.33% |
| *TP53* | SV | splice site 673-1G>A | 3 | 915 | 0.33% |
| *TP53* | SV | E221* | 3 | 915 | 0.33% |
| *TP53* | SV | K132N | 3 | 915 | 0.33% |
| *TP53* | SV | Q104* | 3 | 915 | 0.33% |
| *TP53* | SV | V173M | 3 | 915 | 0.33% |
| *TP53* | SV | P250L | 3 | 915 | 0.33% |
| *TP53* | SV | R158H | 3 | 915 | 0.33% |
| *TP53* | SV | P301fs*44 | 3 | 915 | 0.33% |
| *TP53* | SV | P151R | 3 | 915 | 0.33% |
| *TP53* | SV | R181C | 3 | 915 | 0.33% |
| *TP53* | SV | R181H | 3 | 915 | 0.33% |
| *TP53* | SV | splice site 376-1G>A | 3 | 915 | 0.33% |
| *TP53* | SV | H214R | 3 | 915 | 0.33% |
| *TP53* | SV | M246I | 3 | 915 | 0.33% |
| *TP53* | SV | Y126C | 3 | 915 | 0.33% |
| *TP53* | SV | C238Y | 3 | 915 | 0.33% |
| *TP53* | SV | S127F | 3 | 915 | 0.33% |
| *TP53* | SV | splice site 375+1G>A | 3 | 915 | 0.33% |
| *TP53* | SV | F270L | 3 | 915 | 0.33% |
| *TP53* | SV | V143M | 3 | 915 | 0.33% |
| *TP53* | SV | Y220H | 3 | 915 | 0.33% |
| *TP53* | SV | E294fs*51 | 3 | 915 | 0.33% |
| *TP53* | SV | V274F | 3 | 915 | 0.33% |
| *TP53* | SV | V272M | 3 | 915 | 0.33% |
| *TP53* | SV | L194R | 3 | 915 | 0.33% |
| *TP53* | SV | splice site 672+1G>A | 2 | 915 | 0.22% |
| *TP53* | SV | L130R | 2 | 915 | 0.22% |
| *TP53* | SV | R248P | 2 | 915 | 0.22% |
| *TP53* | SV | R249K | 2 | 915 | 0.22% |
| *TP53* | SV | E224D | 2 | 915 | 0.22% |
| *TP53* | SV | P151A | 2 | 915 | 0.22% |
| *TP53* | SV | L257V | 2 | 915 | 0.22% |
| *TP53* | SV | P177R | 2 | 915 | 0.22% |
| *TP53* | SV | H179L | 2 | 915 | 0.22% |
| *TP53* | SV | S240R | 2 | 915 | 0.22% |
| *TP53* | SV | G244D | 2 | 915 | 0.22% |
| *TP53* | SV | splice site 673-2A>G | 2 | 915 | 0.22% |
| *TP53* | SV | E326* | 2 | 915 | 0.22% |
| *TP53* | SV | Q317* | 2 | 915 | 0.22% |
| *TP53* | SV | R337G | 2 | 915 | 0.22% |
| *TP53* | SV | R249M | 2 | 915 | 0.22% |
| *TP53* | SV | N239D | 2 | 915 | 0.22% |
| *TP53* | SV | I232S | 2 | 915 | 0.22% |
| *TP53* | SV | T253P | 2 | 915 | 0.22% |
| *TP53* | SV | splice site 994-1G>A | 2 | 915 | 0.22% |
| *TP53* | SV | L35fs*9 | 2 | 915 | 0.22% |
| *TP53* | SV | splice site 920-1G>T | 2 | 915 | 0.22% |
| *TP53* | SV | splice site 560-1G>A | 2 | 915 | 0.22% |
| *TP53* | SV | C135R | 2 | 915 | 0.22% |
| *TP53* | SV | S241T | 2 | 915 | 0.22% |
| *TP53* | SV | R158C | 2 | 915 | 0.22% |
| *TP53* | SV | splice site 375+2T>A | 2 | 915 | 0.22% |
| *TP53* | SV | I232F | 2 | 915 | 0.22% |
| *TP53* | SV | L145Q | 2 | 915 | 0.22% |
| *TP53* | SV | S127Y | 2 | 915 | 0.22% |
| *TP53* | SV | T155I | 2 | 915 | 0.22% |
| *TP53* | SV | R280I | 2 | 915 | 0.22% |
| *TP53* | SV | R333fs*12 | 2 | 915 | 0.22% |
| *TP53* | SV | G245S | 2 | 915 | 0.22% |
| *TP53* | SV | P278S | 2 | 915 | 0.22% |
| *TP53* | SV | C277F | 2 | 915 | 0.22% |
| *TP53* | SV | F270I | 2 | 915 | 0.22% |
| *TP53* | SV | splice site 376-2A>G | 2 | 915 | 0.22% |
| *TP53* | SV | E286K | 2 | 915 | 0.22% |
| *TP53* | SV | M1T | 2 | 915 | 0.22% |
| *TP53* | SV | E258* | 2 | 915 | 0.22% |
| *TP53* | SV | L257Q | 2 | 915 | 0.22% |
| *TP53* | SV | splice site 560-2A>C | 2 | 915 | 0.22% |
| *TP53* | SV | A138V | 2 | 915 | 0.22% |
| *TP53* | SV | S215G | 2 | 915 | 0.22% |
| *TP53* | SV | Y107D | 2 | 915 | 0.22% |
| *TP53* | SV | C141W | 2 | 915 | 0.22% |
| *TP53* | SV | R337H | 2 | 915 | 0.22% |
| *TP53* | SV | splice site 782+1G>A | 2 | 915 | 0.22% |
| *TP53* | SV | S215N | 2 | 915 | 0.22% |
| *TP53* | SV | Q136* | 2 | 915 | 0.22% |
| *TP53* | SV | G154V | 2 | 915 | 0.22% |
| *TP53* | SV | R267W | 2 | 915 | 0.22% |
| *TP53* | SV | E285K | 2 | 915 | 0.22% |
| *TP53* | SV | K139N | 2 | 915 | 0.22% |
| *TP53* | SV | E171G | 2 | 915 | 0.22% |
| *TP53* | SV | Y327* | 2 | 915 | 0.22% |
| *TP53* | SV | E271K | 2 | 915 | 0.22% |
| *TP53* | SV | Y205C | 2 | 915 | 0.22% |
| *TP53* | SV | splice site 559+1G>T | 2 | 915 | 0.22% |
| *TP53* | SV | Q165* | 2 | 915 | 0.22% |
| *TP53* | SV | W91* | 2 | 915 | 0.22% |
| *TP53* | SV | E286V | 2 | 915 | 0.22% |
| *TP53* | SV | R306* | 2 | 915 | 0.22% |
| *TP53* | SV | G262V | 2 | 915 | 0.22% |
| *TP53* | SV | P190S | 2 | 915 | 0.22% |
| *TP53* | SV | N131del | 2 | 915 | 0.22% |
| *TP53* | SV | P177_C182del | 2 | 915 | 0.22% |
| *TP53* | SV | P152L | 2 | 915 | 0.22% |
| *TP53* | SV | V173L | 2 | 915 | 0.22% |
| *TP53* | SV | Y163N | 2 | 915 | 0.22% |
| *TP53* | SV | R156P | 2 | 915 | 0.22% |
| *TP53* | SV | splice site 673-1G>C | 2 | 915 | 0.22% |
| *TP53* | SV | C176S | 2 | 915 | 0.22% |
| *TP53* | SV | G266R | 2 | 915 | 0.22% |
| *TP53* | SV | T125M | 2 | 915 | 0.22% |
| *TP53* | SV | splice site 993+2T>A | 2 | 915 | 0.22% |
| *TP53* | SV | R280K | 2 | 915 | 0.22% |
| *TP53* | SV | splice site 672+1G>T | 2 | 915 | 0.22% |
| *TP53* | SV | A276D | 2 | 915 | 0.22% |
| *TP53* | SV | D259Y | 2 | 915 | 0.22% |
| *TP53* | SV | I195F | 2 | 915 | 0.22% |
| *TP53* | SV | S215R | 2 | 915 | 0.22% |
| *TP53* | SV | splice site 375G>A | 2 | 915 | 0.22% |
| *TP53* | SV | G244V | 2 | 915 | 0.22% |
| *TP53* | SV | C176Y | 2 | 915 | 0.22% |
| *TP53* | SV | G245C | 2 | 915 | 0.22% |
| *TP53* | SV | I195T | 2 | 915 | 0.22% |
| *TP53* | SV | I195S | 2 | 915 | 0.22% |
| *TP53* | SV | S240fs*20 | 1 | 915 | 0.11% |
| *TP53* | SV | splice site 97-2A>T | 1 | 915 | 0.11% |
| *TP53* | SV | V172F | 1 | 915 | 0.11% |
| *TP53* | SV | N247fs*98 | 1 | 915 | 0.11% |
| *TP53* | SV | C238F | 1 | 915 | 0.11% |
| *TP53* | SV | Y126H | 1 | 915 | 0.11% |
| *TP53* | SV | K164* | 1 | 915 | 0.11% |
| *TP53* | SV | Y234* | 1 | 915 | 0.11% |
| *TP53* | SV | R209fs*6 | 1 | 915 | 0.11% |
| *TP53* | SV | splice site 375+1G>T | 1 | 915 | 0.11% |
| *TP53* | SV | P190R | 1 | 915 | 0.11% |
| *TP53* | SV | Q167* | 1 | 915 | 0.11% |
| *TP53* | SV | I50fs*64 | 1 | 915 | 0.11% |
| *TP53* | SV | R181G | 1 | 915 | 0.11% |
| *TP53* | SV | N288fs*57 | 1 | 915 | 0.11% |
| *TP53* | SV | N268I | 1 | 915 | 0.11% |
| *TP53* | SV | splice site 783-5_800>TCCCA | 1 | 915 | 0.11% |
| *TP53* | SV | V122fs*47 | 1 | 915 | 0.11% |
| *TP53* | SV | W146R | 1 | 915 | 0.11% |
| *TP53* | SV | D148fs*31 | 1 | 915 | 0.11% |
| *TP53* | SV | T150fs*20 | 1 | 915 | 0.11% |
| *TP53* | SV | V157fs*23 | 1 | 915 | 0.11% |
| *TP53* | SV | L130H | 1 | 915 | 0.11% |
| *TP53* | SV | A70fs*79 | 1 | 915 | 0.11% |
| *TP53* | SV | splice site 97-2A>C | 1 | 915 | 0.11% |
| *TP53* | SV | splice site 1101-1G>A | 1 | 915 | 0.11% |
| *TP53* | SV | S303fs*42 | 1 | 915 | 0.11% |
| *TP53* | SV | P278T | 1 | 915 | 0.11% |
| *TP53* | SV | S241F | 1 | 915 | 0.11% |
| *TP53* | SV | D49fs*74 | 1 | 915 | 0.11% |
| *TP53* | SV | N263fs*5 | 1 | 915 | 0.11% |
| *TP53* | SV | V272E | 1 | 915 | 0.11% |
| *TP53* | SV | G105D | 1 | 915 | 0.11% |
| *TP53* | SV | P151H | 1 | 915 | 0.11% |
| *TP53* | SV | splice site 911_993+23del198 | 1 | 915 | 0.11% |
| *TP53* | SV | N310fs*33 | 1 | 915 | 0.11% |
| *TP53* | SV | splice site 375+2T>C | 1 | 915 | 0.11% |
| *TP53* | SV | L194H | 1 | 915 | 0.11% |
| *TP53* | SV | P219fs*31 | 1 | 915 | 0.11% |
| *TP53* | SV | K132M | 1 | 915 | 0.11% |
| *TP53* | SV | splice site 993+1G>T | 1 | 915 | 0.11% |
| *TP53* | SV | S149fs*18 | 1 | 915 | 0.11% |
| *TP53* | SV | H193L | 1 | 915 | 0.11% |
| *TP53* | SV | G105A | 1 | 915 | 0.11% |
| *TP53* | SV | V157G | 1 | 915 | 0.11% |
| *TP53* | SV | S90fs*59 | 1 | 915 | 0.11% |
| *TP53* | SV | splice site 85_96+3AACAACGTTCTGGTA>CG | 1 | 915 | 0.11% |
| *TP53* | SV | *215Qext*68 | 1 | 915 | 0.11% |
| *TP53* | SV | T125P | 1 | 915 | 0.11% |
| *TP53* | SV | P177L | 1 | 915 | 0.11% |
| *TP53* | SV | P151T | 1 | 915 | 0.11% |
| *TP53* | SV | splice site 919+2T>A | 1 | 915 | 0.11% |
| *TP53* | SV | C242S | 1 | 915 | 0.11% |
| *TP53* | SV | P153fs*16 | 1 | 915 | 0.11% |
| *TP53* | SV | I255F | 1 | 915 | 0.11% |
| *TP53* | SV | R280G | 1 | 915 | 0.11% |
| *TP53* | SV | L93fs*30 | 1 | 915 | 0.11% |
| *TP53* | SV | C135F | 1 | 915 | 0.11% |
| *TP53* | SV | M169I | 1 | 915 | 0.11% |
| *TP53* | SV | D281G | 1 | 915 | 0.11% |
| *TP53* | SV | L111P | 1 | 915 | 0.11% |
| *TP53* | SV | I254S | 1 | 915 | 0.11% |
| *TP53* | SV | P36fs*8 | 1 | 915 | 0.11% |
| *TP53* | SV | F212fs*3 | 1 | 915 | 0.11% |
| *TP53* | SV | N247S | 1 | 915 | 0.11% |
| *TP53* | SV | P47fs*65 | 1 | 915 | 0.11% |
| *TP53* | SV | S241A | 1 | 915 | 0.11% |
| *TP53* | SV | N239S | 1 | 915 | 0.11% |
| *TP53* | SV | Y205N | 1 | 915 | 0.11% |
| *TP53* | SV | E258D | 1 | 915 | 0.11% |
| *TP53* | SV | N239_S240insN | 1 | 915 | 0.11% |
| *TP53* | SV | A86fs*63 | 1 | 915 | 0.11% |
| *TP53* | SV | T284fs*21 | 1 | 915 | 0.11% |
| *TP53* | SV | E336* | 1 | 915 | 0.11% |
| *TP53* | SV | Y220N | 1 | 915 | 0.11% |
| *TP53* | SV | F113I | 1 | 915 | 0.11% |
| *TP53* | SV | I255S | 1 | 915 | 0.11% |
| *TP53* | SV | splice site 783-9_790del17 | 1 | 915 | 0.11% |
| *TP53* | SV | splice site 94_96+31del34 | 1 | 915 | 0.11% |
| *TP53* | SV | P316fs*30 | 1 | 915 | 0.11% |
| *TP53* | SV | splice site 376-8_381delTCCTACAGTACTCC | 1 | 915 | 0.11% |
| *TP53* | SV | S116C | 1 | 915 | 0.11% |
| *TP53* | SV | splice site 920-2A>C | 1 | 915 | 0.11% |
| *TP53* | SV | G59fs*85 | 1 | 915 | 0.11% |
| *TP53* | SV | P72fs*76 | 1 | 915 | 0.11% |
| *TP53* | SV | H233L | 1 | 915 | 0.11% |
| *TP53* | SV | R280T | 1 | 915 | 0.11% |
| *TP53* | SV | D281E | 1 | 915 | 0.11% |
| *TP53* | SV | T253A | 1 | 915 | 0.11% |
| *TP53* | SV | S94* | 1 | 915 | 0.11% |
| *TP53* | SV | R342fs*3 | 1 | 915 | 0.11% |
| *TP53* | SV | F212I | 1 | 915 | 0.11% |
| *TP53* | SV | H179Y | 1 | 915 | 0.11% |
| *TP53* | SV | A159V | 1 | 915 | 0.11% |
| *TP53* | SV | A83fs*40 | 1 | 915 | 0.11% |
| *TP53* | SV | S149fs*20 | 1 | 915 | 0.11% |
| *TP53* | SV | splice site 617_672+109>CTG | 1 | 915 | 0.11% |
| *TP53* | SV | E171* | 1 | 915 | 0.11% |
| *TP53* | SV | P152fs*14 | 1 | 915 | 0.11% |
| *TP53* | SV | C135fs*35 | 1 | 915 | 0.11% |
| *TP53* | SV | D281Y | 1 | 915 | 0.11% |
| *TP53* | SV | P177S | 1 | 915 | 0.11% |
| *TP53* | SV | splice site 372_375+3delCACGGTC | 1 | 915 | 0.11% |
| *TP53* | SV | G266fs*81 | 1 | 915 | 0.11% |
| *TP53* | SV | A84V | 1 | 915 | 0.11% |
| *TP53* | SV | R280S | 1 | 915 | 0.11% |
| *TP53* | SV | H297fs*10 | 1 | 915 | 0.11% |
| *TP53* | SV | P142_V143insCP | 1 | 915 | 0.11% |
| *TP53* | SV | L43* | 1 | 915 | 0.11% |
| *TP53* | SV | K132* | 1 | 915 | 0.11% |
| *TP53* | SV | N239fs*24 | 1 | 915 | 0.11% |
| *TP53* | SV | A39fs*5 | 1 | 915 | 0.11% |
| *TP53* | SV | splice site 560-2A>T | 1 | 915 | 0.11% |
| *TP53* | SV | R335fs*10 | 1 | 915 | 0.11% |
| *TP53* | SV | splice site 993+2T>C | 1 | 915 | 0.11% |
| *TP53* | SV | F113L | 1 | 915 | 0.11% |
| *TP53* | SV | C141R | 1 | 915 | 0.11% |
| *TP53* | SV | I254V | 1 | 915 | 0.11% |
| *TP53* | SV | P152T | 1 | 915 | 0.11% |
| *TP53* | SV | T253N | 1 | 915 | 0.11% |
| *TP53* | SV | H168R | 1 | 915 | 0.11% |
| *TP53* | SV | splice site 672_672+2GGT>CGG | 1 | 915 | 0.11% |
| *TP53* | SV | G262fs*83 | 1 | 915 | 0.11% |
| *TP53* | SV | T170fs*3 | 1 | 915 | 0.11% |
| *TP53* | SV | M160fs*10 | 1 | 915 | 0.11% |
| *TP53* | SV | Y205S | 1 | 915 | 0.11% |
| *TP53* | SV | splice site 920-1G>A | 1 | 915 | 0.11% |
| *TP53* | SV | splice site 672+2T>C | 1 | 915 | 0.11% |
| *TP53* | SV | S183* | 1 | 915 | 0.11% |
| *TP53* | SV | splice site 373_375+9delACGGTCAGTTGC | 1 | 915 | 0.11% |
| *TP53* | SV | T284fs*22 | 1 | 915 | 0.11% |
| *TP53* | SV | W53fs*70 | 1 | 915 | 0.11% |
| *TP53* | SV | splice site 560-1G>C | 1 | 915 | 0.11% |
| *TP53* | SV | F134L | 1 | 915 | 0.11% |
| *TP53* | SV | D208V | 1 | 915 | 0.11% |
| *TP53* | SV | A159fs*21 | 1 | 915 | 0.11% |
| *TP53* | SV | R342Q | 1 | 915 | 0.11% |
| *TP53* | SV | I251F | 1 | 915 | 0.11% |
| *TP53* | SV | V203M | 1 | 915 | 0.11% |
| *TP53* | SV | A138_C141del | 1 | 915 | 0.11% |
| *TP53* | SV | P316fs*19 | 1 | 915 | 0.11% |
| *TP53* | SV | L194P | 1 | 915 | 0.11% |
| *TP53* | SV | Y107fs*40 | 1 | 915 | 0.11% |
| *TP53* | SV | G187fs*60 | 1 | 915 | 0.11% |
| *TP53* | SV | K120E | 1 | 915 | 0.11% |
| *TP53* | SV | K164fs*6 | 1 | 915 | 0.11% |
| *TP53* | SV | E198* | 1 | 915 | 0.11% |
| *TP53* | SV | K139* | 1 | 915 | 0.11% |
| *TP53* | SV | Y236S | 1 | 915 | 0.11% |
| *TP53* | SV | M246fs*1 | 1 | 915 | 0.11% |
| *TP53* | SV | K132R | 1 | 915 | 0.11% |
| *TP53* | SV | M246L | 1 | 915 | 0.11% |
| *TP53* | SV | A161S | 1 | 915 | 0.11% |
| *TP53* | SV | M160I | 1 | 915 | 0.11% |
| *TP53* | SV | D48fs*4 | 1 | 915 | 0.11% |
| *TP53* | SV | R174W | 1 | 915 | 0.11% |
| *TP53* | SV | R196fs*1 | 1 | 915 | 0.11% |
| *TP53* | SV | G105S | 1 | 915 | 0.11% |
| *TP53* | SV | P77fs*69 | 1 | 915 | 0.11% |
| *TP53* | SV | N131fs*18 | 1 | 915 | 0.11% |
| *TP53* | SV | M237V | 1 | 915 | 0.11% |
| *TP53* | SV | splice site 919+1G>T | 1 | 915 | 0.11% |
| *TP53* | SV | V157L | 1 | 915 | 0.11% |
| *TP53* | SV | Q375* | 1 | 915 | 0.11% |
| *TP53* | SV | L289V | 1 | 915 | 0.11% |
| *TP53* | SV | V157A | 1 | 915 | 0.11% |
| *TP53* | SV | P128fs*42 | 1 | 915 | 0.11% |
| *TP53* | SV | S241Y | 1 | 915 | 0.11% |
| *TP53* | SV | H179fs*65 | 1 | 915 | 0.11% |
| *TP53* | SV | H178fs*69 | 1 | 915 | 0.11% |
| *TP53* | SV | S240fs*7 | 1 | 915 | 0.11% |
| *TP53* | SV | splice site 961_993+195del228 | 1 | 915 | 0.11% |
| *TP53* | SV | C275F | 1 | 915 | 0.11% |
| *TP53* | SV | T125R | 1 | 915 | 0.11% |
| *TP53* | SV | T155N | 1 | 915 | 0.11% |
| *TP53* | SV | L145P | 1 | 915 | 0.11% |
| *TP53* | SV | N235_Y236del | 1 | 915 | 0.11% |
| *TP53* | SV | E349* | 1 | 915 | 0.11% |
| *TP53* | SV | C176fs*5 | 1 | 915 | 0.11% |
| *TP53* | SV | Y163* | 1 | 915 | 0.11% |
| *TP53* | SV | K381fs*3 | 1 | 915 | 0.11% |
| *TP53* | SV | R283P | 1 | 915 | 0.11% |
| *TP53* | SV | Y236H | 1 | 915 | 0.11% |
| *TP53* | SV | G199V | 1 | 915 | 0.11% |
| *TP53* | SV | P36fs*7 | 1 | 915 | 0.11% |
| *TP53* | SV | E343* | 1 | 915 | 0.11% |
| *TP53* | SV | P278R | 1 | 915 | 0.11% |
| *TP53* | SV | splice site 673-2A>C | 1 | 915 | 0.11% |
| *TP53* | SV | A79fs*48 | 1 | 915 | 0.11% |
| *TP53* | SV | I232fs*15 | 1 | 915 | 0.11% |
| *TP53* | SV | V272L | 1 | 915 | 0.11% |
| *TP53* | SV | splice site 902_919+2>G | 1 | 915 | 0.11% |
| *TP53* | SV | K132E | 1 | 915 | 0.11% |
| *TP53* | SV | P85fs*38 | 1 | 915 | 0.11% |
| *TP53* | SV | splice site 993+1G>A | 1 | 915 | 0.11% |
| *TP53* | SV | splice site 994-2A>T | 1 | 915 | 0.11% |
| *TP53* | SV | G334fs*10 | 1 | 915 | 0.11% |
| *TP53* | SV | L265R | 1 | 915 | 0.11% |
| *TP53* | SV | L289fs*16 | 1 | 915 | 0.11% |
| *TP53* | SV | splice site 672G>A | 1 | 915 | 0.11% |
| *TP53* | SV | splice site 920-2A>G | 1 | 915 | 0.11% |
| *TP53* | SV | Y220D | 1 | 915 | 0.11% |
| *TP53* | SV | P219fs*3 | 1 | 915 | 0.11% |
| *TP53* | SV | C124fs*25 | 1 | 915 | 0.11% |
| *TP53* | SV | L330fs*15 | 1 | 915 | 0.11% |
| *TP53* | SV | K320* | 1 | 915 | 0.11% |
| *TP53* | SV | E339* | 1 | 915 | 0.11% |
| *TP53* | SV | H179P | 1 | 915 | 0.11% |
| *TP53* | SV | A161fs*17 | 1 | 915 | 0.11% |
| *TP53* | SV | L201fs*46 | 1 | 915 | 0.11% |
| *TP53* | SV | splice site 97-1G>A | 1 | 915 | 0.11% |
| *TP53* | SV | W146fs*2 | 1 | 915 | 0.11% |
| *TP53* | SV | M160fs*11 | 1 | 915 | 0.11% |
| *TP53* | SV | H179Q | 1 | 915 | 0.11% |
| *TP53* | SV | C275S | 1 | 915 | 0.11% |
| *TP53* | SV | P151_P152insPPGTRVRAT | 1 | 915 | 0.11% |
| *TP53* | SV | Q331H | 1 | 915 | 0.11% |
| *TP53* | SV | S127_P128insS | 1 | 915 | 0.11% |
| *TP53* | SV | I251T | 1 | 915 | 0.11% |
| *TP53* | SV | Y205F | 1 | 915 | 0.11% |
| *TP53* | SV | Q136H | 1 | 915 | 0.11% |
| *TP53* | SV | L188fs*55 | 1 | 915 | 0.11% |
| *TP53* | SV | S183fs*1 | 1 | 915 | 0.11% |
| *TP53* | SV | splice site 559+1G>C | 1 | 915 | 0.11% |
| *TP53* | SV | G244C | 1 | 915 | 0.11% |
| *TP53* | SV | splice site 920-1G>C | 1 | 915 | 0.11% |
| *TP53* | SV | splice site 783-150_799del165 | 1 | 915 | 0.11% |
| *TP53* | SV | T81fs*42 | 1 | 915 | 0.11% |
| *TP53* | SV | P190fs*57 | 1 | 915 | 0.11% |
| *TP53* | SV | I162F | 1 | 915 | 0.11% |
| *TP53* | SV | G266* | 1 | 915 | 0.11% |
| *TP53* | SV | Y220del | 1 | 915 | 0.11% |
| *TP53* | SV | S106_L114>M | 1 | 915 | 0.11% |
| *TP53* | SV | V157_R158insV | 1 | 915 | 0.11% |
| *TP53* | SV | F338fs*4 | 1 | 915 | 0.11% |
| *TP53* | SV | N235D | 1 | 915 | 0.11% |
| *TP53* | SV | N263fs*30 | 1 | 915 | 0.11% |
| *TP53* | SV | M243I | 1 | 915 | 0.11% |
| *TP53* | SV | P67fs*56 | 1 | 915 | 0.11% |
| *TP53* | SV | D228fs*12 | 1 | 915 | 0.11% |
| *TP53* | SV | F113C | 1 | 915 | 0.11% |
| *TP53* | SV | Q136E | 1 | 915 | 0.11% |
| *TP53* | SV | C238fs*1 | 1 | 915 | 0.11% |
| *TP53* | SV | V147G | 1 | 915 | 0.11% |
| *TP53* | SV | Y126* | 1 | 915 | 0.11% |
| *TP53* | SV | V157D | 1 | 915 | 0.11% |
| *TP53* | SV | L330H | 1 | 915 | 0.11% |
| *TP53* | SV | G245R | 1 | 915 | 0.11% |
| *TP53* | SV | P177T | 1 | 915 | 0.11% |
| *TP53* | SV | K101* | 1 | 915 | 0.11% |
| *TP53* | SV | E171fs*2 | 1 | 915 | 0.11% |
| *TP53* | SV | Y220S | 1 | 915 | 0.11% |
| *TP53* | SV | splice site 376-1G>T | 1 | 915 | 0.11% |
| *TP53* | SV | splice site 783-1G>A | 1 | 915 | 0.11% |
| *TP53* | SV | V173A | 1 | 915 | 0.11% |
| *TP53* | SV | R249G | 1 | 915 | 0.11% |
| *TP53* | SV | N247I | 1 | 915 | 0.11% |
| *TP53* | SV | R283H | 1 | 915 | 0.11% |
| *TP53* | SV | L130fs*19 | 1 | 915 | 0.11% |
| *TP53* | SV | C242F | 1 | 915 | 0.11% |
| *TP53* | SV | V73fs*76 | 1 | 915 | 0.11% |
| *TP53* | SV | V216G | 1 | 915 | 0.11% |
| *TP53* | SV | R181P | 1 | 915 | 0.11% |
| *TP53* | SV | A347P | 1 | 915 | 0.11% |
| *TP53* | SV | K373fs*49 | 1 | 915 | 0.11% |
| *TP53* | SV | splice site 783-2A>C | 1 | 915 | 0.11% |
| *TP53* | SV | V97fs*55 | 1 | 915 | 0.11% |
| *TP53* | SV | Y234D | 1 | 915 | 0.11% |
| *TP53* | SV | D259V | 1 | 915 | 0.11% |
| *TP53* | SV | W53* | 1 | 915 | 0.11% |
| *TP53* | SV | splice site 559+1G>A | 1 | 915 | 0.11% |
| *TP53* | SV | R175C | 1 | 915 | 0.11% |
| *TP53* | SV | R175L | 1 | 915 | 0.11% |
| *TP53* | SV | E346* | 1 | 915 | 0.11% |
| *TP53* | SV | K305* | 1 | 915 | 0.11% |
| *TP53* | SV | E287K | 1 | 915 | 0.11% |
| *TP53* | SV | S314fs*30 | 1 | 915 | 0.11% |
| *TP53* | SV | A129G | 1 | 915 | 0.11% |
| *TP53* | SV | splice site 75-1G>A | 1 | 915 | 0.11% |
| *TP53* | SV | splice site 96+1G>A | 1 | 915 | 0.11% |
| *TP53* | SV | P87fs*36 | 1 | 915 | 0.11% |
| *TP53* | SV | A159P | 1 | 915 | 0.11% |
| *TP53* | SV | splice site 75-1G>C | 1 | 915 | 0.11% |
| *TP53* | SV | M237fs*25 | 1 | 915 | 0.11% |
| *TP53* | SV | M243L | 1 | 915 | 0.11% |
| *TP53* | SV | C229fs*9 | 1 | 915 | 0.11% |
| *TP53* | SV | N131S | 1 | 915 | 0.11% |
| *TP53* | SV | S261fs*78 | 1 | 915 | 0.11% |
| *TP53* | SV | K164N | 1 | 915 | 0.11% |
| *TP53* | SV | F134fs*1 | 1 | 915 | 0.11% |
| *TP53* | SV | C275_D281del | 1 | 915 | 0.11% |
| *TP53* | SV | splice site 560-5_563delCTTAGGTCT | 1 | 915 | 0.11% |
| *TP53* | SV | K120fs*3 | 1 | 915 | 0.11% |
| *TP53* | SV | T231del | 1 | 915 | 0.11% |
| *TP53* | SV | P250F | 1 | 915 | 0.11% |
| *TP53* | SV | C176G | 1 | 915 | 0.11% |
| *TP53* | SV | P219H | 1 | 915 | 0.11% |
| *TP53* | SV | S260fs*85 | 1 | 915 | 0.11% |
| *TP53* | SV | D186fs*22 | 1 | 915 | 0.11% |
| *TP53* | SV | S20fs*24 | 1 | 915 | 0.11% |
| *TP53* | SV | L130V | 1 | 915 | 0.11% |
| *TP53* | SV | V197M | 1 | 915 | 0.11% |
| *TP53* | SV | R282P | 1 | 915 | 0.11% |

**Supplementary Table 3: Copy number alteration in HCC patients.**

| **Gene** | **Copy number alteration** | **Incidence** | **Total Population** | **Frequency (%)** |
| --- | --- | --- | --- | --- |
| *MYC* | amplification | 330 | 2372 | 13.91% |
| *CCND1* | amplification | 150 | 2372 | 6.32% |
| *FGF19* | amplification | 134 | 2372 | 5.65% |
| *FGF4* | amplification | 105 | 2372 | 4.43% |
| *LYN* | amplification | 103 | 2372 | 4.34% |
| *FGF3* | amplification | 101 | 2372 | 4.26% |
| *MCL1* | amplification | 95 | 2372 | 4.01% |
| *NTRK1* | amplification | 57 | 2372 | 2.40% |
| *VEGFA* | amplification | 52 | 2372 | 2.19% |
| *CCNE1* | amplification | 38 | 2372 | 1.60% |
| *MET* | amplification | 36 | 2372 | 1.52% |
| *PIK3C2B* | amplification | 34 | 2372 | 1.43% |
| *MDM4* | amplification | 31 | 2372 | 1.31% |
| *CCND3* | amplification | 27 | 2372 | 1.14% |
| *HGF* | amplification | 27 | 2372 | 1.14% |
| *AKT3* | amplification | 27 | 2372 | 1.14% |
| *IRS2* | amplification | 25 | 2372 | 1.05% |
| *KRAS* | amplification | 22 | 2372 | 0.93% |
| *ZNF217* | amplification | 21 | 2372 | 0.89% |
| *ERBB2* | amplification | 19 | 2372 | 0.80% |
| *RICTOR* | amplification | 19 | 2372 | 0.80% |
| *GNAS* | amplification | 18 | 2372 | 0.76% |
| *CDK6* | amplification | 18 | 2372 | 0.76% |
| *AKT2* | amplification | 17 | 2372 | 0.72% |
| *NOTCH3* | amplification | 17 | 2372 | 0.72% |
| *TERC* | amplification | 16 | 2372 | 0.67% |
| *AURKA* | amplification | 15 | 2372 | 0.63% |
| *PRKCI* | amplification | 14 | 2372 | 0.59% |
| *MDM2* | amplification | 13 | 2372 | 0.55% |
| *FGF14* | amplification | 13 | 2372 | 0.55% |
| *IKBKE* | amplification | 13 | 2372 | 0.55% |
| *ZNF703* | amplification | 12 | 2372 | 0.51% |
| *CDK4* | amplification | 11 | 2372 | 0.46% |
| *AXL* | amplification | 11 | 2372 | 0.46% |
| *FGF10* | amplification | 11 | 2372 | 0.46% |
| *EGFR* | amplification | 11 | 2372 | 0.46% |
| *CRKL* | amplification | 10 | 2372 | 0.42% |
| *FGFR1* | amplification | 10 | 2372 | 0.42% |
| *ARFRP1* | amplification | 10 | 2372 | 0.42% |
| *CD274* | amplification | 9 | 2372 | 0.38% |
| *PDCD1LG2* | amplification | 9 | 2372 | 0.38% |
| *MYCL* | amplification | 9 | 2372 | 0.38% |
| *EMSY* | amplification | 8 | 2372 | 0.34% |
| *JAK2* | amplification | 8 | 2372 | 0.34% |
| *RPTOR* | amplification | 8 | 2372 | 0.34% |
| *JUN* | amplification | 7 | 2372 | 0.30% |
| *CCND2* | amplification | 7 | 2372 | 0.30% |
| *FGF23* | amplification | 7 | 2372 | 0.30% |
| *FGF6* | amplification | 7 | 2372 | 0.30% |
| *BCL2L1* | amplification | 7 | 2372 | 0.30% |
| *BCL2L2* | amplification | 6 | 2372 | 0.25% |
| *KDM5A* | amplification | 6 | 2372 | 0.25% |
| *GID4* | amplification | 6 | 2372 | 0.25% |
| *EPHA3* | amplification | 6 | 2372 | 0.25% |
| *SRC* | amplification | 5 | 2372 | 0.21% |
| *NFKBIA* | amplification | 4 | 2372 | 0.17% |
| *NKX2-1* | amplification | 4 | 2372 | 0.17% |
| *RET* | amplification | 4 | 2372 | 0.17% |
| *KIT* | amplification | 4 | 2372 | 0.17% |
| *PDGFRA* | amplification | 4 | 2372 | 0.17% |
| *ERBB4* | amplification | 3 | 2372 | 0.13% |
| *ERBB3* | amplification | 3 | 2372 | 0.13% |
| *FGFR3* | amplification | 3 | 2372 | 0.13% |
| *GATA6* | amplification | 3 | 2372 | 0.13% |
| *BRAF* | amplification | 3 | 2372 | 0.13% |
| *MYCN* | amplification | 3 | 2372 | 0.13% |
| *AR* | amplification | 2 | 2372 | 0.08% |
| *PIK3CA* | amplification | 2 | 2372 | 0.08% |
| *MAP2K1* | amplification | 2 | 2372 | 0.08% |
| *NRAS* | amplification | 2 | 2372 | 0.08% |
| *KDR* | amplification | 2 | 2372 | 0.08% |
| *RAF1* | amplification | 2 | 2372 | 0.08% |
| *CDK8* | amplification | 2 | 2372 | 0.08% |
| *MITF* | amplification | 2 | 2372 | 0.08% |
| *ALK* | amplification | 2 | 2372 | 0.08% |
| *HRAS* | amplification | 2 | 2372 | 0.08% |
| *IGF1R* | amplification | 1 | 2372 | 0.04% |
| *AKT1* | amplification | 1 | 2372 | 0.04% |
| *SOX2* | amplification | 1 | 2372 | 0.04% |
| *PIK3CB* | amplification | 1 | 2372 | 0.04% |
| *FGFR2* | amplification | 1 | 2372 | 0.04% |
| *EPHB1* | amplification | 1 | 2372 | 0.04% |
| *AURKB* | amplification | 1 | 2372 | 0.04% |
| *MTOR* | amplification | 1 | 2372 | 0.04% |
| *PDGFRB* | amplification | 1 | 2372 | 0.04% |
| *FGFR4* | amplification | 1 | 2372 | 0.04% |
| *KEL* | amplification | 1 | 2372 | 0.04% |
| *CDKN2A* | deletion | 121 | 2372 | 5.10% |
| *CDKN2B* | deletion | 105 | 2372 | 4.43% |
| *PTEN* | deletion | 73 | 2372 | 3.08% |
| *RB1* | deletion | 64 | 2372 | 2.70% |
| *STK11* | deletion | 32 | 2372 | 1.35% |
| *TP53* | deletion | 28 | 2372 | 1.18% |
| *TSC2* | deletion | 20 | 2372 | 0.84% |
| *FAS* | deletion | 14 | 2372 | 0.59% |
| *ARID1A* | deletion | 11 | 2372 | 0.46% |
| *IRF2* | deletion | 11 | 2372 | 0.46% |
| *TSC1* | deletion | 10 | 2372 | 0.42% |
| *APC* | deletion | 8 | 2372 | 0.34% |
| *SMAD4* | deletion | 7 | 2372 | 0.30% |
| *KDM6A* | deletion | 7 | 2372 | 0.30% |
| *CDKN2C* | deletion | 7 | 2372 | 0.30% |
| *KEAP1* | deletion | 6 | 2372 | 0.25% |
| *ERRFI1* | deletion | 5 | 2372 | 0.21% |
| *BCOR* | deletion | 5 | 2372 | 0.21% |
| *SMARCA4* | deletion | 5 | 2372 | 0.21% |
| *PBRM1* | deletion | 5 | 2372 | 0.21% |
| *MAP2K4* | deletion | 4 | 2372 | 0.17% |
| *PIK3R1* | deletion | 3 | 2372 | 0.13% |
| *BRCA2* | deletion | 3 | 2372 | 0.13% |
| *BCORL1* | deletion | 3 | 2372 | 0.13% |
| *BRCA1* | deletion | 3 | 2372 | 0.13% |
| *NF1* | deletion | 3 | 2372 | 0.13% |
| *BAP1* | deletion | 3 | 2372 | 0.13% |
| *CREBBP* | deletion | 3 | 2372 | 0.13% |
| *FLCN* | deletion | 3 | 2372 | 0.13% |
| *PRKN* | deletion | 3 | 2372 | 0.13% |
| *BARD1* | deletion | 2 | 2372 | 0.08% |
| *KDM5C* | deletion | 2 | 2372 | 0.08% |
| *FANCA* | deletion | 2 | 2372 | 0.08% |
| *CDKN1A* | deletion | 2 | 2372 | 0.08% |
| *CHEK2* | deletion | 2 | 2372 | 0.08% |
| *STAG2* | deletion | 2 | 2372 | 0.08% |
| *CDKN1B* | deletion | 2 | 2372 | 0.08% |
| *TET2* | deletion | 1 | 2372 | 0.04% |
| *ATRX* | deletion | 1 | 2372 | 0.04% |
| *SMAD2* | deletion | 1 | 2372 | 0.04% |
| *MLH1* | deletion | 1 | 2372 | 0.04% |
| *PRDM1* | deletion | 1 | 2372 | 0.04% |
| *TNFRSF14* | deletion | 1 | 2372 | 0.04% |
| *QKI* | deletion | 1 | 2372 | 0.04% |
| *CBFB* | deletion | 1 | 2372 | 0.04% |
| *ATM* | deletion | 1 | 2372 | 0.04% |
| *GATA4* | deletion | 1 | 2372 | 0.04% |
| *CTNNA1* | deletion | 1 | 2372 | 0.04% |
| *ACVR1B* | deletion | 1 | 2372 | 0.04% |

**Supplementary Table 4: Rearrangements in HCC patients.**

| **Gene** | **Alteration Type** | **Incidence** | **Total Population** | **Frequency (%)** |
| --- | --- | --- | --- | --- |
| *CDKN2A* | RE | 15 | 2372 | 0.63% |
| *ARID1A* | RE | 12 | 2372 | 0.51% |
| *CTNNB1* | RE | 10 | 2372 | 0.42% |
| *RB1* | RE | 10 | 2372 | 0.42% |
| *BRCA1* | RE | 10 | 2372 | 0.42% |
| *APC* | RE | 9 | 2372 | 0.38% |
| *FANCA* | RE | 8 | 2372 | 0.34% |
| *TP53* | RE | 7 | 2372 | 0.30% |
| *TSC2* | RE | 7 | 2372 | 0.30% |
| *PBRM1* | RE | 7 | 2372 | 0.30% |
| *NF1* | RE | 7 | 2372 | 0.30% |
| *CREBBP* | RE | 7 | 2372 | 0.30% |
| *MYC* | RE | 6 | 2372 | 0.25% |
| *STK11* | RE | 6 | 2372 | 0.25% |
| *NF2* | RE | 6 | 2372 | 0.25% |
| *CHEK2* | RE | 6 | 2372 | 0.25% |
| *ATM* | RE | 6 | 2372 | 0.25% |
| *BAP1* | RE | 6 | 2372 | 0.25% |
| *PTEN* | RE | 5 | 2372 | 0.21% |
| *FGFR3* | RE | 5 | 2372 | 0.21% |
| *KMT2D* | RE | 4 | 2372 | 0.17% |
| *SMARCA4* | RE | 4 | 2372 | 0.17% |
| *BRD4* | RE | 4 | 2372 | 0.17% |
| *CDC73* | RE | 4 | 2372 | 0.17% |
| *SPEN* | RE | 3 | 2372 | 0.13% |
| *MYB* | RE | 3 | 2372 | 0.13% |
| *KEAP1* | RE | 3 | 2372 | 0.13% |
| *KDM6A* | RE | 3 | 2372 | 0.13% |
| *ROS1* | RE | 3 | 2372 | 0.13% |
| *NTRK1* | RE | 3 | 2372 | 0.13% |
| *EGFR* | RE | 3 | 2372 | 0.13% |
| *EZH2* | RE | 3 | 2372 | 0.13% |
| *CEBPA* | RE | 3 | 2372 | 0.13% |
| *PIK3R1* | RE | 3 | 2372 | 0.13% |
| *FANCL* | RE | 2 | 2372 | 0.08% |
| *NOTCH1* | RE | 2 | 2372 | 0.08% |
| *NOTCH3* | RE | 2 | 2372 | 0.08% |
| *TSC1* | RE | 2 | 2372 | 0.08% |
| *ACVR1B* | RE | 2 | 2372 | 0.08% |
| *EP300* | RE | 2 | 2372 | 0.08% |
| *RAF1* | RE | 2 | 2372 | 0.08% |
| *CDKN1B* | RE | 2 | 2372 | 0.08% |
| *ETV6* | RE | 2 | 2372 | 0.08% |
| *PTCH1* | RE | 2 | 2372 | 0.08% |
| *BRIP1* | RE | 2 | 2372 | 0.08% |
| *FGFR1* | RE | 2 | 2372 | 0.08% |
| *MLH1* | RE | 2 | 2372 | 0.08% |
| *BRCA2* | RE | 2 | 2372 | 0.08% |
| *MAP2K4* | RE | 2 | 2372 | 0.08% |
| *PRKN* | RE | 2 | 2372 | 0.08% |
| *IRF2* | RE | 2 | 2372 | 0.08% |
| *CDKN2B* | RE | 2 | 2372 | 0.08% |
| *FBXW7* | RE | 1 | 2372 | 0.04% |
| *SMARCB1* | RE | 1 | 2372 | 0.04% |
| *SETD2* | RE | 1 | 2372 | 0.04% |
| *SOCS1* | RE | 1 | 2372 | 0.04% |
| *SDHC* | RE | 1 | 2372 | 0.04% |
| *FANCC* | RE | 1 | 2372 | 0.04% |
| *KEL* | RE | 1 | 2372 | 0.04% |
| *FUBP1* | RE | 1 | 2372 | 0.04% |
| *ABL1* | RE | 1 | 2372 | 0.04% |
| *CBL* | RE | 1 | 2372 | 0.04% |
| *MRE11* | RE | 1 | 2372 | 0.04% |
| *FGFR2* | RE | 1 | 2372 | 0.04% |
| *DNMT3A* | RE | 1 | 2372 | 0.04% |
| *GATA4* | RE | 1 | 2372 | 0.04% |
| *CDKN2C* | RE | 1 | 2372 | 0.04% |
| *STAG2* | RE | 1 | 2372 | 0.04% |
| *FLCN* | RE | 1 | 2372 | 0.04% |
| *ATR* | RE | 1 | 2372 | 0.04% |
| *BARD1* | RE | 1 | 2372 | 0.04% |
| *BCL2* | RE | 1 | 2372 | 0.04% |
| *DAXX* | RE | 1 | 2372 | 0.04% |
| *MAP2K2* | RE | 1 | 2372 | 0.04% |
| *ATRX* | RE | 1 | 2372 | 0.04% |
| *SDHD* | RE | 1 | 2372 | 0.04% |
| *FH* | RE | 1 | 2372 | 0.04% |
| *CD274* | RE | 1 | 2372 | 0.04% |
| *ALK* | RE | 1 | 2372 | 0.04% |
| *KDM5C* | RE | 1 | 2372 | 0.04% |
| *FGFR4* | RE | 1 | 2372 | 0.04% |
| *CSF1R* | RE | 1 | 2372 | 0.04% |
| *PRDM1* | RE | 1 | 2372 | 0.04% |
| *SMAD4* | RE | 1 | 2372 | 0.04% |
| *MEN1* | RE | 1 | 2372 | 0.04% |
| *BCOR* | RE | 1 | 2372 | 0.04% |
| *JAK2* | RE | 1 | 2372 | 0.04% |
| *PDGFRA* | RE | 1 | 2372 | 0.04% |
| *RET* | RE | 1 | 2372 | 0.04% |
| *ERRFI1* | RE | 1 | 2372 | 0.04% |
| *NOTCH2* | RE | 1 | 2372 | 0.04% |
| *TET2* | RE | 1 | 2372 | 0.04% |

**Supplementary Table 5: AXIN1 variants initially classified as variants of unknown significance.**

| ***AXIN1* variant** | **n** | **final evaluation** |
| --- | --- | --- |
| 'P494fs*211' | 1 | pathogenic |
| 'M1V' | 3 | likely benign |
| 'V742I' | 2 | benign |
| 'R492fs*213' | 1 | pathogenic |
| 'AXIN1_RE' | 6 | VUS |
| 'I637fs*2' | 1 | pathogenic |
| 'H534Q' | 3 | benign |
| 'T104fs*31' | 1 | pathogenic |
| 'V805fs*8' | 1 | pathogenic |
| 'splice site 1020-1G>A' | 1 | pathogenic |
| 'G650fs*55' | 1 | pathogenic |
| 'Q476fs*4' | 1 | pathogenic |
| 'R395L' | 1 | likely benign |
| 'splice site 1116+1G>C' | 1 | pathogenic |
| 'R604fs*100' | 1 | pathogenic |
| 'G508fs*197' | 2 | pathogenic |
| 'D151fs*22' | 1 | pathogenic |
| 'D151E' | 1 | benign |
| 'E358*' | 1 | pathogenic |
| 'G455fs*24' | 1 | pathogenic |
| 'G856R' | 1 | likely benign |
| 'V800_R803>C' | 1 | likely benign |
| 'A443fs*37' | 1 | pathogenic |
| 'K232*' | 1 | pathogenic |
| 'deletion' | 25 | pathogenic |
| 'R712*' | 1 | pathogenic |
| 'splice site 1116+1G>A' | 4 | pathogenic |
| 'K136*' | 1 | pathogenic |
| 'G154fs*30' | 1 | pathogenic |
| 'R647S' | 1 | likely benign |
| 'R712Q' | 1 | pathogenic |
| 'W635*' | 1 | pathogenic |
| 'K719fs*82' | 1 | pathogenic |
| 'V366fs*48' | 1 | pathogenic |
| 'L807fs*8' | 1 | pathogenic |
| 'Q224fs*18' | 1 | pathogenic |
| 'P449H' | 1 | likely benign |
| 'R680fs*100' | 1 | pathogenic |
| 'N133fs*3' | 1 | pathogenic |
| 'E423*' | 1 | pathogenic |
| 'G483C' | 1 | likely benign |
| 'P661S' | 1 | benign |
| 'T230fs*12' | 1 | pathogenic |
| 'Q357*' | 1 | pathogenic |
| 'R158W' | 1 | likely benign |
| 'L592P' | 1 | likely benign |
| 'amplification' | 1 | likely benign |
| 'W674*' | 1 | pathogenic |
| 'splice site 2295-1G>A' | 1 | pathogenic |
| 'A86fs*14' | 1 | pathogenic |
| 'R841*' | 2 | pathogenic |
| 'splice site 878+1G>A' | 1 | pathogenic |
| 'A522fs*68' | 1 | pathogenic |
| 'I234M' | 1 | benign |
| 'V836L' | 1 | likely benign |
| 'R290*' | 1 | pathogenic |
| 'G49S' | 1 | benign |
| 'L312fs*104' | 1 | pathogenic |
| 'E627*' | 1 | pathogenic |
| 'G856fs*82' | 1 | pathogenic |
| 'splice site 1785-2A>C' | 1 | pathogenic |
| 'A804S' | 1 | benign |
| 'CD2AP' | 1 | VUS |
| 'splice site 1019+1G>A' | 1 | pathogenic |
| 'E243*' | 1 | pathogenic |
| 'C130fs*20' | 1 | pathogenic |
| 'H530fs*61' | 1 | pathogenic |
| 'N242D' | 1 | benign |
| 'D781fs*1' | 1 | pathogenic |
| 'S644R' | 1 | benign |
| 'E642fs*22' | 1 | pathogenic |
| 'A505T' | 1 | benign |
| 'A509fs*82' | 1 | pathogenic |
| 'K628*' | 1 | pathogenic |
| 'Q108*' | 1 | pathogenic |
| 'R365W' | 1 | likely benign |
| 'E842*' | 1 | pathogenic |
| 'splice site 1255-1G>A' | 1 | pathogenic |
| 'M735I' | 1 | benign |
| 'Q184*' | 1 | pathogenic |
| 'splice site 1785-2A>T' | 1 | pathogenic |
| 'D97fs*35' | 1 | pathogenic |
| 'G775fs*4' | 2 | pathogenic |
| 'Q6fs*24' | 1 | pathogenic |
| 'K519*' | 1 | pathogenic |
| 'K248*' | 2 | pathogenic |
| 'splice site 1956-2A>T' | 1 | pathogenic |
| 'Y148H' | 1 | likely benign |
| 'A745P' | 1 | benign |
| 'A40T' | 1 | benign |
| 'K519fs*186' | 1 | pathogenic |
| 'S429*' | 1 | pathogenic |
| 'Y305fs*109' | 1 | pathogenic |
| 'K165R' | 1 | benign |
| 'PGAP6' | 1 | VUS |
| 'W297*' | 1 | pathogenic |
| 'Q559*' | 1 | pathogenic |
| 'R484fs*216' | 1 | pathogenic |
| 'E709fs*92' | 1 | pathogenic |
| 'Y70*' | 1 | pathogenic |
| 'L12fs*11' | 1 | pathogenic |
| 'splice site 1113_1116+2delTCCCGT' | 1 | likely benign |
| 'N133fs*6' | 1 | pathogenic |
| 'D862fs*22' | 1 | pathogenic |
| 'Q186P' | 1 | likely benign |
| 'S561N' | 1 | benign |
| 'Q357L' | 1 | likely benign |
| '1020-3_1050del34' | 1 | benign |
| 'S654fs*10' | 1 | pathogenic |
| 'S560fs*145' | 1 | pathogenic |
| 'V301I' | 1 | benign |
| 'K203fs*39' | 1 | pathogenic |
| 'E715*' | 1 | pathogenic |
| 'S493fs*209' | 1 | pathogenic |
| 'P129S' | 1 | benign |
| 'E384*' | 1 | pathogenic |
| 'E618fs*87' | 1 | pathogenic |
| 'Q251fs*163' | 1 | pathogenic |
| 'M759T' | 1 | benign |
| 'E553*' | 1 | pathogenic |
| 'splice site 1020-1_1020-1delG' | 1 | likely benign |
| 'K379fs*43' | 1 | pathogenic |
| 'Q269fs*16' | 1 | pathogenic |
| 'G265fs*149' | 1 | pathogenic |
| 'T213fs*29' | 1 | pathogenic |
| 'H662fs*43' | 1 | pathogenic |
| 'E407*' | 1 | pathogenic |
| 'S589fs*116' | 1 | pathogenic |
| 'splice site 1784_1784+1GG>TT' | 1 | pathogenic |
| 'K219*' | 1 | pathogenic |
| 'K84*' | 1 | pathogenic |
| 'L704fs*1' | 1 | pathogenic |
| 'V377fs*37' | 1 | pathogenic |
| 'G295*' | 1 | pathogenic |
| 'P37L' | 1 | likely benign |
| 'S654fs*9' | 1 | pathogenic |
| 'R284S' | 1 | benign |
| 'F201V' | 1 | likely benign |
| 'R645fs*61' | 1 | pathogenic |
| 'E640fs*65' | 1 | pathogenic |
| 'R648fs*13' | 1 | pathogenic |
| 'Q323*' | 1 | pathogenic |
| 'R373fs*50' | 1 | pathogenic |
| 'S428L' | 1 | likely benign |
| 'K169fs*5' | 1 | pathogenic |
| 'G110V' | 1 | likely benign |
| 'P179fs*63' | 1 | pathogenic |
| 'Q778*' | 1 | pathogenic |
| 'E580*' | 1 | pathogenic |
| 'PIGQ' | 1 | VUS |
| 'E411*' | 1 | pathogenic |
| 'E618*' | 1 | pathogenic |
| 'Q4*' | 1 | pathogenic |
| 'E288*' | 1 | pathogenic |
| 'A120D' | 1 | pathogenic |
| 'C435*' | 1 | pathogenic |
| 'E128*' | 1 | pathogenic |
| 'L326V' | 1 | likely benign |

The functional status of *AXIN1* variants of unknown significance (VUS) was manually re-annotated by a certified molecular pathologist. Consequently, 143 of the 199 VUS cases were reclassified as pathogenic, resulting in a total of 153 pathogenic AXIN1 alterations (6.5%) across all cases. For more information see Supplementary Material and Methods.

**Supplementary Table 6: Comparison of FoundationOne (T7) and F1CDx (DX1/DX2) panels. OR: odds ratio. FDR: false discovery rate.**

| **Gene** | **#Assessable T7** | **#Altered T7** | **Prevalence T7** | **#Assessable DX1/DX2** | **#Altered DX1/DX2** | **Prevalence DX1/DX2** | **OR** | **FDR** |
| --- | --- | --- | --- | --- | --- | --- | --- | --- |
| *TERT* | 867 | 468 | 53.98% | 1505 | 938 | 62.33% | 0.71 | 0.00 |
| *MYC* | 867 | 93 | 10.73% | 1505 | 241 | 16.01% | 0.63 | 0.01 |
| *LYN* | 867 | 26 | 3.00% | 1505 | 79 | 5.25% | 0.56 | 0.10 |
| *PIK3C2B* | 867 | 20 | 2.31% | 1505 | 14 | 0.93% | 2.51 | 0.10 |
| *CTNNB1* | 867 | 267 | 30.80% | 1505 | 541 | 35.95% | 0.79 | 0.10 |
| *MDM4* | 867 | 19 | 2.19% | 1505 | 12 | 0.80% | 2.79 | 0.10 |
| *NFE2L2* | 867 | 34 | 3.92% | 1505 | 91 | 6.05% | 0.63 | 0.21 |
| *ARID1A* | 867 | 91 | 10.50% | 1505 | 200 | 13.29% | 0.77 | 0.33 |
| *NOTCH2* | 867 | 15 | 1.73% | 1505 | 13 | 0.86% | 2.02 | 0.43 |
| *GNAS* | 867 | 19 | 2.19% | 1505 | 19 | 1.26% | 1.75 | 0.47 |
| *BRCA2* | 867 | 14 | 1.61% | 1505 | 12 | 0.80% | 2.04 | 0.47 |
| *TP53* | 867 | 305 | 35.18% | 1505 | 570 | 37.87% | 0.89 | 0.55 |
| *NF1* | 867 | 21 | 2.42% | 1505 | 24 | 1.59% | 1.53 | 0.55 |
| *TSC2* | 867 | 34 | 3.92% | 1505 | 80 | 5.32% | 0.73 | 0.55 |
| *APC* | 867 | 24 | 2.77% | 1505 | 28 | 1.86% | 1.50 | 0.55 |
| *TSC1* | 867 | 15 | 1.73% | 1505 | 39 | 2.59% | 0.66 | 0.55 |
| *VEGFA* | 867 | 15 | 1.73% | 1505 | 39 | 2.59% | 0.66 | 0.55 |
| *NTRK1* | 867 | 28 | 3.23% | 1505 | 35 | 2.33% | 1.40 | 0.55 |
| *STK11* | 867 | 18 | 2.08% | 1505 | 46 | 3.06% | 0.67 | 0.55 |
| *KEAP1* | 867 | 38 | 4.38% | 1505 | 51 | 3.39% | 1.31 | 0.57 |
| *CUL4A* | 867 | 13 | 1.50% | 1505 | 14 | 0.93% | 1.62 | 0.57 |
| *MCL1* | 867 | 40 | 4.61% | 1505 | 56 | 3.72% | 1.25 | 0.73 |
| *ERBB2* | 867 | 10 | 1.15% | 1505 | 11 | 0.73% | 1.58 | 0.73 |
| *CDKN2A* | 867 | 74 | 8.54% | 1505 | 111 | 7.38% | 1.17 | 0.73 |
| *CDKN1A* | 867 | 14 | 1.61% | 1505 | 34 | 2.26% | 0.71 | 0.73 |
| *KMT2D* | 867 | 24 | 2.77% | 1505 | 32 | 2.13% | 1.31 | 0.73 |
| *CDKN1B* | 867 | 16 | 1.85% | 1505 | 21 | 1.40% | 1.33 | 0.73 |
| *CCND1* | 867 | 50 | 5.77% | 1505 | 102 | 6.78% | 0.84 | 0.73 |
| *FGF3* | 867 | 33 | 3.81% | 1505 | 68 | 4.52% | 0.84 | 0.77 |
| *TET2* | 867 | 15 | 1.73% | 1505 | 34 | 2.26% | 0.76 | 0.77 |
| *PIK3CA* | 867 | 16 | 1.85% | 1505 | 36 | 2.39% | 0.77 | 0.77 |
| *CDKN2B* | 867 | 43 | 4.96% | 1505 | 65 | 4.32% | 1.16 | 0.77 |
| *IRS2* | 867 | 11 | 1.27% | 1505 | 15 | 1.00% | 1.28 | 0.86 |
| *FGF4* | 867 | 36 | 4.15% | 1505 | 69 | 4.58% | 0.90 | 0.95 |
| *EGFR* | 867 | 10 | 1.15% | 1505 | 14 | 0.93% | 1.24 | 0.95 |
| *KRAS* | 867 | 13 | 1.50% | 1505 | 28 | 1.86% | 0.80 | 0.95 |
| *FGF19* | 867 | 46 | 5.31% | 1505 | 88 | 5.85% | 0.90 | 0.95 |
| *CREBBP* | 867 | 12 | 1.38% | 1505 | 24 | 1.59% | 0.87 | 1.00 |
| *SMARCA4* | 867 | 13 | 1.50% | 1505 | 21 | 1.40% | 1.08 | 1.00 |
| *DNMT3A* | 867 | 23 | 2.65% | 1505 | 43 | 2.86% | 0.93 | 1.00 |
| *CCNE1* | 867 | 15 | 1.73% | 1505 | 24 | 1.59% | 1.09 | 1.00 |
| *SETD2* | 867 | 11 | 1.27% | 1505 | 21 | 1.40% | 0.91 | 1.00 |
| *ATM* | 867 | 26 | 3.00% | 1505 | 46 | 3.06% | 0.98 | 1.00 |
| *RB1* | 867 | 67 | 7.73% | 1505 | 114 | 7.57% | 1.02 | 1.00 |
| *BRCA1* | 867 | 10 | 1.15% | 1505 | 15 | 1.00% | 1.16 | 1.00 |
| *MET* | 867 | 16 | 1.85% | 1505 | 28 | 1.86% | 0.99 | 1.00 |
| *MUTYH* | 867 | 18 | 2.08% | 1505 | 34 | 2.26% | 0.92 | 1.00 |
| *PTEN* | 867 | 46 | 5.31% | 1505 | 78 | 5.18% | 1.03 | 1.00 |
| *BAP1* | 867 | 26 | 3.00% | 1505 | 42 | 2.79% | 1.08 | 1.00 |
| *CHEK2* | 867 | 17 | 1.96% | 1505 | 28 | 1.86% | 1.06 | 1.00 |
| *AKT3* | 867 | 10 | 1.15% | 1505 | 17 | 1.13% | 1.02 | 1.00 |
| *PBRM1* | 867 | 16 | 1.85% | 1505 | 28 | 1.86% | 0.99 | 1.00 |
| *GREM1* | 867 | 0 | 0.00% | 1505 | 0 | 0.00% | . | . |
| *BARD1* | 867 | 4 | 0.46% | 1505 | 8 | 0.53% | . | . |
| *TSHR* | 867 | 0 | 0.00% | 1505 | 0 | 0.00% | . | . |
| *MYCL* | 867 | 6 | 0.69% | 1505 | 3 | 0.20% | . | . |
| *NOTCH4* | 867 | 0 | 0.00% | 1505 | 0 | 0.00% | . | . |
| *ERG* | 867 | 0 | 0.00% | 1505 | 1 | 0.07% | . | . |
| *FGF14* | 867 | 5 | 0.58% | 1505 | 9 | 0.60% | . | . |
| *MYB* | 867 | 2 | 0.23% | 1505 | 1 | 0.07% | . | . |
| *SDHD* | 867 | 0 | 0.00% | 1505 | 1 | 0.07% | . | . |
| *CD274* | 867 | 3 | 0.35% | 1505 | 6 | 0.40% | . | . |
| *PPP2R2A* | 867 | 0 | 0.00% | 1505 | 0 | 0.00% | . | . |
| *MSH2* | 867 | 2 | 0.23% | 1505 | 4 | 0.27% | . | . |
| *PARP2* | 867 | 0 | 0.00% | 1505 | 0 | 0.00% | . | . |
| *NOTCH3* | 867 | 7 | 0.81% | 1505 | 21 | 1.40% | . | . |
| *MITF* | 867 | 2 | 0.23% | 1505 | 2 | 0.13% | . | . |
| *LRP6* | 867 | 0 | 0.00% | 1505 | 0 | 0.00% | . | . |
| *SRSF2* | 867 | 0 | 0.00% | 1505 | 0 | 0.00% | . | . |
| *EPHA7* | 867 | 0 | 0.00% | 1505 | 0 | 0.00% | . | . |
| *PRKAR1A* | 867 | 3 | 0.35% | 1505 | 6 | 0.40% | . | . |
| *EPHA5* | 867 | 0 | 0.00% | 1505 | 0 | 0.00% | . | . |
| *FGF10* | 867 | 3 | 0.35% | 1505 | 8 | 0.53% | . | . |
| *GATA4* | 867 | 1 | 0.12% | 1505 | 2 | 0.13% | . | . |
| *SDHA* | 867 | 4 | 0.46% | 1505 | 4 | 0.27% | . | . |
| *CUL4B* | 867 | 0 | 0.00% | 1505 | 0 | 0.00% | . | . |
| *ERCC4* | 867 | 2 | 0.23% | 1505 | 0 | 0.00% | . | . |
| *SGK1* | 867 | 0 | 0.00% | 1505 | 0 | 0.00% | . | . |
| *HGF* | 867 | 4 | 0.46% | 1505 | 24 | 1.59% | . | . |
| *FUBP1* | 867 | 2 | 0.23% | 1505 | 3 | 0.20% | . | . |
| *ROS1* | 867 | 1 | 0.12% | 1505 | 3 | 0.20% | . | . |
| *BTK* | 867 | 0 | 0.00% | 1505 | 1 | 0.07% | . | . |
| *ALOX12B* | 867 | 0 | 0.00% | 1505 | 1 | 0.07% | . | . |
| *SOX2* | 867 | 0 | 0.00% | 1505 | 1 | 0.07% | . | . |
| *PLCG2* | 867 | 0 | 0.00% | 1505 | 0 | 0.00% | . | . |
| *BMPR1A* | 867 | 0 | 0.00% | 1505 | 0 | 0.00% | . | . |
| *FANCL* | 867 | 2 | 0.23% | 1505 | 3 | 0.20% | . | . |
| *NRAS* | 867 | 9 | 1.04% | 1505 | 8 | 0.53% | . | . |
| *MKNK1* | 867 | 0 | 0.00% | 1505 | 0 | 0.00% | . | . |
| *BCL2L1* | 867 | 3 | 0.35% | 1505 | 5 | 0.33% | . | . |
| *NCOR1* | 867 | 0 | 0.00% | 1505 | 0 | 0.00% | . | . |
| *ARFRP1* | 867 | 3 | 0.35% | 1505 | 7 | 0.47% | . | . |
| *AKT2* | 867 | 7 | 0.81% | 1505 | 11 | 0.73% | . | . |
| *CDK12* | 867 | 2 | 0.23% | 1505 | 3 | 0.20% | . | . |
| *SF3B1* | 867 | 8 | 0.92% | 1505 | 22 | 1.46% | . | . |
| *MST1R* | 867 | 0 | 0.00% | 1505 | 1 | 0.07% | . | . |
| *GABRA6* | 867 | 0 | 0.00% | 1505 | 1 | 0.07% | . | . |
| *RPA1* | 867 | 0 | 0.00% | 1505 | 0 | 0.00% | . | . |
| *ETV1* | 867 | 0 | 0.00% | 1505 | 0 | 0.00% | . | . |
| *AKT1* | 867 | 2 | 0.23% | 1505 | 2 | 0.13% | . | . |
| *SDHB* | 867 | 1 | 0.12% | 1505 | 1 | 0.07% | . | . |
| *PIK3CB* | 867 | 1 | 0.12% | 1505 | 1 | 0.07% | . | . |
| *BCL6* | 867 | 0 | 0.00% | 1505 | 0 | 0.00% | . | . |
| *WT1* | 867 | 2 | 0.23% | 1505 | 4 | 0.27% | . | . |
| *BRD4* | 867 | 2 | 0.23% | 1505 | 5 | 0.33% | . | . |
| *CCN6* | 867 | 0 | 0.00% | 1505 | 0 | 0.00% | . | . |
| *MTOR* | 867 | 3 | 0.35% | 1505 | 8 | 0.53% | . | . |
| *GNAQ* | 867 | 1 | 0.12% | 1505 | 1 | 0.07% | . | . |
| *FANCM* | 867 | 0 | 0.00% | 1505 | 0 | 0.00% | . | . |
| *CALR* | 867 | 0 | 0.00% | 1505 | 0 | 0.00% | . | . |
| *ERBB3* | 867 | 3 | 0.35% | 1505 | 2 | 0.13% | . | . |
| *CIC* | 867 | 0 | 0.00% | 1505 | 4 | 0.27% | . | . |
| *MPL* | 867 | 0 | 0.00% | 1505 | 0 | 0.00% | . | . |
| *FANCE* | 867 | 0 | 0.00% | 1505 | 0 | 0.00% | . | . |
| *TRRAP* | 867 | 0 | 0.00% | 1505 | 0 | 0.00% | . | . |
| *PIM1* | 867 | 0 | 0.00% | 1505 | 0 | 0.00% | . | . |
| *IGF2R* | 867 | 0 | 0.00% | 1505 | 0 | 0.00% | . | . |
| *RET* | 867 | 6 | 0.69% | 1505 | 5 | 0.33% | . | . |
| *IGF1R* | 867 | 0 | 0.00% | 1505 | 2 | 0.13% | . | . |
| *QKI* | 867 | 0 | 0.00% | 1505 | 5 | 0.33% | . | . |
| *MTAP* | 867 | 0 | 0.00% | 1505 | 0 | 0.00% | . | . |
| *MAP2K4* | 867 | 7 | 0.81% | 1505 | 8 | 0.53% | . | . |
| *INSR* | 867 | 0 | 0.00% | 1505 | 0 | 0.00% | . | . |
| *FGFR4* | 867 | 3 | 0.35% | 1505 | 0 | 0.00% | . | . |
| *RAD21* | 867 | 0 | 0.00% | 1505 | 0 | 0.00% | . | . |
| *AXL* | 867 | 4 | 0.46% | 1505 | 8 | 0.53% | . | . |
| *EZR* | 867 | 0 | 0.00% | 1505 | 0 | 0.00% | . | . |
| *PAX5* | 867 | 1 | 0.12% | 1505 | 0 | 0.00% | . | . |
| *RAD51* | 867 | 0 | 0.00% | 1505 | 1 | 0.07% | . | . |
| *GNA13* | 867 | 1 | 0.12% | 1505 | 0 | 0.00% | . | . |
| *P2RY8* | 867 | 0 | 0.00% | 1505 | 0 | 0.00% | . | . |
| *FOXL2* | 867 | 0 | 0.00% | 1505 | 0 | 0.00% | . | . |
| *CXCR4* | 867 | 0 | 0.00% | 1505 | 0 | 0.00% | . | . |
| *RICTOR* | 867 | 6 | 0.69% | 1505 | 14 | 0.93% | . | . |
| *SYK* | 867 | 0 | 0.00% | 1505 | 0 | 0.00% | . | . |
| *NOTCH1* | 867 | 3 | 0.35% | 1505 | 9 | 0.60% | . | . |
| *RARA* | 867 | 0 | 0.00% | 1505 | 0 | 0.00% | . | . |
| *NUP93* | 867 | 0 | 0.00% | 1505 | 0 | 0.00% | . | . |
| *EZH2* | 867 | 0 | 0.00% | 1505 | 4 | 0.27% | . | . |
| *SNCAIP* | 867 | 0 | 0.00% | 1505 | 0 | 0.00% | . | . |
| *AXIN1* | 867 | 5 | 0.58% | 1505 | 5 | 0.33% | . | . |
| *REL* | 867 | 1 | 0.12% | 1505 | 0 | 0.00% | . | . |
| *SOX9* | 867 | 3 | 0.35% | 1505 | 3 | 0.20% | . | . |
| *MSH3* | 867 | 0 | 0.00% | 1505 | 0 | 0.00% | . | . |
| *GATA2* | 867 | 0 | 0.00% | 1505 | 0 | 0.00% | . | . |
| *SMO* | 867 | 0 | 0.00% | 1505 | 0 | 0.00% | . | . |
| *NSD1* | 867 | 0 | 0.00% | 1505 | 0 | 0.00% | . | . |
| *RPTOR* | 867 | 4 | 0.46% | 1505 | 4 | 0.27% | . | . |
| *SRC* | 867 | 1 | 0.12% | 1505 | 4 | 0.27% | . | . |
| *BCL2L2* | 867 | 0 | 0.00% | 1505 | 6 | 0.40% | . | . |
| *CCND3* | 867 | 5 | 0.58% | 1505 | 22 | 1.46% | . | . |
| *CD79B* | 867 | 0 | 0.00% | 1505 | 0 | 0.00% | . | . |
| *KDM5A* | 867 | 3 | 0.35% | 1505 | 3 | 0.20% | . | . |
| *CTNNA1* | 867 | 2 | 0.23% | 1505 | 1 | 0.07% | . | . |
| *SMAD3* | 867 | 0 | 0.00% | 1505 | 0 | 0.00% | . | . |
| *ALK* | 867 | 2 | 0.23% | 1505 | 4 | 0.27% | . | . |
| *CBL* | 867 | 4 | 0.46% | 1505 | 16 | 1.06% | . | . |
| *GLI1* | 867 | 0 | 0.00% | 1505 | 0 | 0.00% | . | . |
| *RAD51D* | 867 | 0 | 0.00% | 1505 | 3 | 0.20% | . | . |
| *NF2* | 867 | 3 | 0.35% | 1505 | 14 | 0.93% | . | . |
| *TMPRSS2* | 867 | 0 | 0.00% | 1505 | 0 | 0.00% | . | . |
| *MLH1* | 867 | 1 | 0.12% | 1505 | 8 | 0.53% | . | . |
| *PARP4* | 867 | 0 | 0.00% | 1505 | 0 | 0.00% | . | . |
| *PTCH1* | 867 | 2 | 0.23% | 1505 | 4 | 0.27% | . | . |
| *XRCC2* | 867 | 1 | 0.12% | 1505 | 0 | 0.00% | . | . |
| *PDGFRB* | 867 | 0 | 0.00% | 1505 | 5 | 0.33% | . | . |
| *IKZF1* | 867 | 0 | 0.00% | 1505 | 0 | 0.00% | . | . |
| *HDAC1* | 867 | 0 | 0.00% | 1505 | 0 | 0.00% | . | . |
| *SPTA1* | 867 | 0 | 0.00% | 1505 | 0 | 0.00% | . | . |
| *IGF1* | 867 | 0 | 0.00% | 1505 | 0 | 0.00% | . | . |
| *PDCD1LG2* | 867 | 3 | 0.35% | 1505 | 6 | 0.40% | . | . |
| *MYD88* | 867 | 0 | 0.00% | 1505 | 2 | 0.13% | . | . |
| *SMARCB1* | 867 | 3 | 0.35% | 1505 | 8 | 0.53% | . | . |
| *TP53BP1* | 867 | 0 | 0.00% | 1505 | 0 | 0.00% | . | . |
| *TNKS* | 867 | 0 | 0.00% | 1505 | 0 | 0.00% | . | . |
| *CARD11* | 867 | 0 | 0.00% | 1505 | 2 | 0.13% | . | . |
| *SOCS1* | 867 | 0 | 0.00% | 1505 | 1 | 0.07% | . | . |
| *FGF12* | 867 | 0 | 0.00% | 1505 | 0 | 0.00% | . | . |
| *NTRK3* | 867 | 1 | 0.12% | 1505 | 2 | 0.13% | . | . |
| *PTCH2* | 867 | 0 | 0.00% | 1505 | 0 | 0.00% | . | . |
| *CASP8* | 867 | 7 | 0.81% | 1505 | 11 | 0.73% | . | . |
| *RAD50* | 867 | 0 | 0.00% | 1505 | 0 | 0.00% | . | . |
| *CYP17A1* | 867 | 0 | 0.00% | 1505 | 0 | 0.00% | . | . |
| *EPHB6* | 867 | 0 | 0.00% | 1505 | 0 | 0.00% | . | . |
| *KEL* | 867 | 5 | 0.58% | 1505 | 8 | 0.53% | . | . |
| *KDM6A* | 867 | 5 | 0.58% | 1505 | 17 | 1.13% | . | . |
| *AR* | 867 | 2 | 0.23% | 1505 | 3 | 0.20% | . | . |
| *FLT1* | 867 | 1 | 0.12% | 1505 | 0 | 0.00% | . | . |
| *NTRK2* | 867 | 0 | 0.00% | 1505 | 0 | 0.00% | . | . |
| *CHUK* | 867 | 0 | 0.00% | 1505 | 0 | 0.00% | . | . |
| *PALB2* | 867 | 3 | 0.35% | 1505 | 5 | 0.33% | . | . |
| *PPP2R1A* | 867 | 1 | 0.12% | 1505 | 1 | 0.07% | . | . |
| *FANCG* | 867 | 0 | 0.00% | 1505 | 2 | 0.13% | . | . |
| *PTPRD* | 867 | 0 | 0.00% | 1505 | 0 | 0.00% | . | . |
| *ARID1B* | 867 | 0 | 0.00% | 1505 | 0 | 0.00% | . | . |
| *ABRAXAS1* | 867 | 0 | 0.00% | 1505 | 0 | 0.00% | . | . |
| *PARP1* | 867 | 19 | 2.19% | 1505 | 5 | 0.33% | . | . |
| *SLIT2* | 867 | 0 | 0.00% | 1505 | 0 | 0.00% | . | . |
| *HNF1A* | 867 | 2 | 0.23% | 1505 | 6 | 0.40% | . | . |
| *GATA6* | 867 | 0 | 0.00% | 1505 | 3 | 0.20% | . | . |
| *PTPN11* | 867 | 5 | 0.58% | 1505 | 10 | 0.66% | . | . |
| *ASXL1* | 867 | 6 | 0.69% | 1505 | 17 | 1.13% | . | . |
| *MRE11* | 867 | 1 | 0.12% | 1505 | 6 | 0.40% | . | . |
| *ABL2* | 867 | 0 | 0.00% | 1505 | 0 | 0.00% | . | . |
| *RAC1* | 867 | 0 | 0.00% | 1505 | 0 | 0.00% | . | . |
| *RUNX1* | 867 | 0 | 0.00% | 1505 | 0 | 0.00% | . | . |
| *JAK3* | 867 | 0 | 0.00% | 1505 | 0 | 0.00% | . | . |
| *LMO1* | 867 | 0 | 0.00% | 1505 | 0 | 0.00% | . | . |
| *TEK* | 867 | 1 | 0.12% | 1505 | 1 | 0.07% | . | . |
| *TBX3* | 867 | 1 | 0.12% | 1505 | 1 | 0.07% | . | . |
| *TYRO3* | 867 | 0 | 0.00% | 1505 | 0 | 0.00% | . | . |
| *CD22* | 867 | 0 | 0.00% | 1505 | 0 | 0.00% | . | . |
| *KMT2A* | 867 | 0 | 0.00% | 1505 | 2 | 0.13% | . | . |
| *DICER1* | 867 | 0 | 0.00% | 1505 | 0 | 0.00% | . | . |
| *SUFU* | 867 | 0 | 0.00% | 1505 | 2 | 0.13% | . | . |
| *AURKA* | 867 | 6 | 0.69% | 1505 | 9 | 0.60% | . | . |
| *B2M* | 867 | 0 | 0.00% | 1505 | 0 | 0.00% | . | . |
| *FANCF* | 867 | 0 | 0.00% | 1505 | 0 | 0.00% | . | . |
| *FAT3* | 867 | 0 | 0.00% | 1505 | 0 | 0.00% | . | . |
| *BCORL1* | 867 | 3 | 0.35% | 1505 | 10 | 0.66% | . | . |
| *GALNT12* | 867 | 0 | 0.00% | 1505 | 0 | 0.00% | . | . |
| *SMAD2* | 867 | 0 | 0.00% | 1505 | 4 | 0.27% | . | . |
| *CYLD* | 867 | 0 | 0.00% | 1505 | 0 | 0.00% | . | . |
| *HSP90AA1* | 867 | 0 | 0.00% | 1505 | 0 | 0.00% | . | . |
| *EMSY* | 867 | 2 | 0.23% | 1505 | 6 | 0.40% | . | . |
| *PRDM1* | 867 | 5 | 0.58% | 1505 | 5 | 0.33% | . | . |
| *NUDT1* | 867 | 0 | 0.00% | 1505 | 0 | 0.00% | . | . |
| *VHL* | 867 | 3 | 0.35% | 1505 | 5 | 0.33% | . | . |
| *FGFR2* | 867 | 3 | 0.35% | 1505 | 5 | 0.33% | . | . |
| *ZNRF3* | 867 | 0 | 0.00% | 1505 | 0 | 0.00% | . | . |
| *CHEK1* | 867 | 0 | 0.00% | 1505 | 1 | 0.07% | . | . |
| *CDH20* | 867 | 0 | 0.00% | 1505 | 0 | 0.00% | . | . |
| *KDM5C* | 867 | 1 | 0.12% | 1505 | 4 | 0.27% | . | . |
| *GRM3* | 867 | 2 | 0.23% | 1505 | 1 | 0.07% | . | . |
| *FANCA* | 867 | 9 | 1.04% | 1505 | 20 | 1.33% | . | . |
| *MKNK2* | 867 | 0 | 0.00% | 1505 | 0 | 0.00% | . | . |
| *LRP1B* | 867 | 0 | 0.00% | 1505 | 0 | 0.00% | . | . |
| *LZTR1* | 867 | 0 | 0.00% | 1505 | 0 | 0.00% | . | . |
| *GSK3B* | 867 | 0 | 0.00% | 1505 | 0 | 0.00% | . | . |
| *GATA1* | 867 | 0 | 0.00% | 1505 | 0 | 0.00% | . | . |
| *SPEN* | 867 | 4 | 0.46% | 1505 | 8 | 0.53% | . | . |
| *RBM10* | 867 | 8 | 0.92% | 1505 | 8 | 0.53% | . | . |
| *MDM2* | 867 | 4 | 0.46% | 1505 | 9 | 0.60% | . | . |
| *ERBB4* | 867 | 0 | 0.00% | 1505 | 7 | 0.47% | . | . |
| *PRSS1* | 867 | 0 | 0.00% | 1505 | 0 | 0.00% | . | . |
| *PDGFRA* | 867 | 2 | 0.23% | 1505 | 5 | 0.33% | . | . |
| *HRAS* | 867 | 2 | 0.23% | 1505 | 0 | 0.00% | . | . |
| *TENT5C* | 867 | 0 | 0.00% | 1505 | 0 | 0.00% | . | . |
| *EPHA3* | 867 | 3 | 0.35% | 1505 | 7 | 0.47% | . | . |
| *TNKS2* | 867 | 0 | 0.00% | 1505 | 0 | 0.00% | . | . |
| *PRKCI* | 867 | 4 | 0.46% | 1505 | 10 | 0.66% | . | . |
| *DDR1* | 867 | 1 | 0.12% | 1505 | 1 | 0.07% | . | . |
| *SDHC* | 867 | 0 | 0.00% | 1505 | 2 | 0.13% | . | . |
| *CRLF2* | 867 | 0 | 0.00% | 1505 | 0 | 0.00% | . | . |
| *FOXP1* | 867 | 0 | 0.00% | 1505 | 0 | 0.00% | . | . |
| *STAT3* | 867 | 1 | 0.12% | 1505 | 10 | 0.66% | . | . |
| *XRCC3* | 867 | 0 | 0.00% | 1505 | 0 | 0.00% | . | . |
| *IL7R* | 867 | 0 | 0.00% | 1505 | 0 | 0.00% | . | . |
| *JUN* | 867 | 2 | 0.23% | 1505 | 5 | 0.33% | . | . |
| *CDK8* | 867 | 0 | 0.00% | 1505 | 2 | 0.13% | . | . |
| *HLA-A* | 867 | 0 | 0.00% | 1505 | 0 | 0.00% | . | . |
| *CDH1* | 867 | 2 | 0.23% | 1505 | 8 | 0.53% | . | . |
| *RANBP2* | 867 | 0 | 0.00% | 1505 | 0 | 0.00% | . | . |
| *CDH5* | 867 | 0 | 0.00% | 1505 | 0 | 0.00% | . | . |
| *ARAF* | 867 | 0 | 0.00% | 1505 | 1 | 0.07% | . | . |
| *GID4* | 867 | 3 | 0.35% | 1505 | 3 | 0.20% | . | . |
| *FANCD2* | 867 | 0 | 0.00% | 1505 | 0 | 0.00% | . | . |
| *PIK3C2G* | 867 | 0 | 0.00% | 1505 | 3 | 0.20% | . | . |
| *XPO1* | 867 | 0 | 0.00% | 1505 | 0 | 0.00% | . | . |
| *ATR* | 867 | 4 | 0.46% | 1505 | 12 | 0.80% | . | . |
| *PTPRO* | 867 | 0 | 0.00% | 1505 | 0 | 0.00% | . | . |
| *PIK3R1* | 867 | 5 | 0.58% | 1505 | 8 | 0.53% | . | . |
| *PPARG* | 867 | 0 | 0.00% | 1505 | 0 | 0.00% | . | . |
| *KMT2C* | 867 | 0 | 0.00% | 1505 | 0 | 0.00% | . | . |
| *DIS3* | 867 | 1 | 0.12% | 1505 | 1 | 0.07% | . | . |
| *MEF2B* | 867 | 0 | 0.00% | 1505 | 0 | 0.00% | . | . |
| *CUL3* | 867 | 0 | 0.00% | 1505 | 1 | 0.07% | . | . |
| *JAK1* | 867 | 4 | 0.46% | 1505 | 6 | 0.40% | . | . |
| *FH* | 867 | 1 | 0.12% | 1505 | 2 | 0.13% | . | . |
| *FRS2* | 867 | 0 | 0.00% | 1505 | 0 | 0.00% | . | . |
| *FGF23* | 867 | 4 | 0.46% | 1505 | 6 | 0.40% | . | . |
| *RAF1* | 867 | 2 | 0.23% | 1505 | 2 | 0.13% | . | . |
| *TGFBR2* | 867 | 0 | 0.00% | 1505 | 2 | 0.13% | . | . |
| *NSD2* | 867 | 0 | 0.00% | 1505 | 0 | 0.00% | . | . |
| *KAT6A* | 867 | 0 | 0.00% | 1505 | 0 | 0.00% | . | . |
| *PIK3CG* | 867 | 0 | 0.00% | 1505 | 0 | 0.00% | . | . |
| *CDH2* | 867 | 0 | 0.00% | 1505 | 0 | 0.00% | . | . |
| *CSF1R* | 867 | 1 | 0.12% | 1505 | 2 | 0.13% | . | . |
| *FGFR1* | 867 | 6 | 0.69% | 1505 | 6 | 0.40% | . | . |
| *FLCN* | 867 | 9 | 1.04% | 1505 | 9 | 0.60% | . | . |
| *ETV5* | 867 | 0 | 0.00% | 1505 | 0 | 0.00% | . | . |
| *PIK3R2* | 867 | 0 | 0.00% | 1505 | 0 | 0.00% | . | . |
| *RUNX1T1* | 867 | 0 | 0.00% | 1505 | 0 | 0.00% | . | . |
| *MAP3K1* | 867 | 2 | 0.23% | 1505 | 6 | 0.40% | . | . |
| *DOT1L* | 867 | 1 | 0.12% | 1505 | 0 | 0.00% | . | . |
| *HLA-C* | 867 | 0 | 0.00% | 1505 | 0 | 0.00% | . | . |
| *CHD4* | 867 | 0 | 0.00% | 1505 | 0 | 0.00% | . | . |
| *SLC34A2* | 867 | 0 | 0.00% | 1505 | 0 | 0.00% | . | . |
| *FLT3* | 867 | 0 | 0.00% | 1505 | 0 | 0.00% | . | . |
| *CD70* | 867 | 0 | 0.00% | 1505 | 0 | 0.00% | . | . |
| *TERC* | 867 | 4 | 0.46% | 1505 | 12 | 0.80% | . | . |
| *PIK3C3* | 867 | 0 | 0.00% | 1505 | 0 | 0.00% | . | . |
| *MAP3K13* | 867 | 2 | 0.23% | 1505 | 6 | 0.40% | . | . |
| *DDR2* | 867 | 1 | 0.12% | 1505 | 1 | 0.07% | . | . |
| *H3-3A* | 867 | 1 | 0.12% | 1505 | 0 | 0.00% | . | . |
| *TAF1* | 867 | 0 | 0.00% | 1505 | 0 | 0.00% | . | . |
| *ATRX* | 867 | 7 | 0.81% | 1505 | 11 | 0.73% | . | . |
| *CCND2* | 867 | 6 | 0.69% | 1505 | 4 | 0.27% | . | . |
| *NFKBIA* | 867 | 2 | 0.23% | 1505 | 5 | 0.33% | . | . |
| *RAD52* | 867 | 0 | 0.00% | 1505 | 0 | 0.00% | . | . |
| *RAD51C* | 867 | 2 | 0.23% | 1505 | 1 | 0.07% | . | . |
| *INHBA* | 867 | 0 | 0.00% | 1505 | 0 | 0.00% | . | . |
| *TNFRSF14* | 867 | 0 | 0.00% | 1505 | 2 | 0.13% | . | . |
| *EED* | 867 | 0 | 0.00% | 1505 | 0 | 0.00% | . | . |
| *POLE* | 867 | 1 | 0.12% | 1505 | 0 | 0.00% | . | . |
| *MED12* | 867 | 1 | 0.12% | 1505 | 0 | 0.00% | . | . |
| *ARID2* | 867 | 0 | 0.00% | 1505 | 0 | 0.00% | . | . |
| *SH2B3* | 867 | 0 | 0.00% | 1505 | 0 | 0.00% | . | . |
| *PREX2* | 867 | 0 | 0.00% | 1505 | 0 | 0.00% | . | . |
| *HOXB13* | 867 | 0 | 0.00% | 1505 | 0 | 0.00% | . | . |
| *ETV6* | 867 | 1 | 0.12% | 1505 | 1 | 0.07% | . | . |
| *RAD54L* | 867 | 4 | 0.46% | 1505 | 4 | 0.27% | . | . |
| *PRKDC* | 867 | 0 | 0.00% | 1505 | 0 | 0.00% | . | . |
| *FLT4* | 867 | 0 | 0.00% | 1505 | 0 | 0.00% | . | . |
| *POLD1* | 867 | 0 | 0.00% | 1505 | 0 | 0.00% | . | . |
| *CEBPA* | 867 | 2 | 0.23% | 1505 | 4 | 0.27% | . | . |
| *TOP1* | 867 | 0 | 0.00% | 1505 | 0 | 0.00% | . | . |
| *RAD51B* | 867 | 2 | 0.23% | 1505 | 5 | 0.33% | . | . |
| *CHD2* | 867 | 0 | 0.00% | 1505 | 0 | 0.00% | . | . |
| *PAK3* | 867 | 0 | 0.00% | 1505 | 0 | 0.00% | . | . |
| *FANCC* | 867 | 4 | 0.46% | 1505 | 4 | 0.27% | . | . |
| *BCOR* | 867 | 1 | 0.12% | 1505 | 9 | 0.60% | . | . |
| *ACVR1B* | 867 | 6 | 0.69% | 1505 | 10 | 0.66% | . | . |
| *KIT* | 867 | 1 | 0.12% | 1505 | 4 | 0.27% | . | . |
| *CDC73* | 867 | 1 | 0.12% | 1505 | 5 | 0.33% | . | . |
| *MEN1* | 867 | 7 | 0.81% | 1505 | 6 | 0.40% | . | . |
| *BLM* | 867 | 0 | 0.00% | 1505 | 0 | 0.00% | . | . |
| *BACH1* | 867 | 0 | 0.00% | 1505 | 0 | 0.00% | . | . |
| *CTCF* | 867 | 4 | 0.46% | 1505 | 4 | 0.27% | . | . |
| *PRKN* | 867 | 6 | 0.69% | 1505 | 7 | 0.47% | . | . |
| *AURKB* | 867 | 0 | 0.00% | 1505 | 1 | 0.07% | . | . |
| *HSD3B1* | 867 | 2 | 0.23% | 1505 | 1 | 0.07% | . | . |
| *ESR1* | 867 | 0 | 0.00% | 1505 | 0 | 0.00% | . | . |
| *PAK5* | 867 | 0 | 0.00% | 1505 | 0 | 0.00% | . | . |
| *HLA-B* | 867 | 0 | 0.00% | 1505 | 0 | 0.00% | . | . |
| *STAT4* | 867 | 0 | 0.00% | 1505 | 0 | 0.00% | . | . |
| *CBFB* | 867 | 0 | 0.00% | 1505 | 1 | 0.07% | . | . |
| *KDR* | 867 | 2 | 0.23% | 1505 | 3 | 0.20% | . | . |
| *BRIP1* | 867 | 4 | 0.46% | 1505 | 9 | 0.60% | . | . |
| *CDK4* | 867 | 4 | 0.46% | 1505 | 8 | 0.53% | . | . |
| *SMAD4* | 867 | 9 | 1.04% | 1505 | 10 | 0.66% | . | . |
| *EWSR1* | 867 | 0 | 0.00% | 1505 | 0 | 0.00% | . | . |
| *IDH2* | 867 | 0 | 0.00% | 1505 | 1 | 0.07% | . | . |
| *TIPARP* | 867 | 1 | 0.12% | 1505 | 1 | 0.07% | . | . |
| *EPHB4* | 867 | 4 | 0.46% | 1505 | 5 | 0.33% | . | . |
| *MAPK1* | 867 | 0 | 0.00% | 1505 | 0 | 0.00% | . | . |
| *IKBKE* | 867 | 9 | 1.04% | 1505 | 4 | 0.27% | . | . |
| *EP300* | 867 | 7 | 0.81% | 1505 | 8 | 0.53% | . | . |
| *ZBTB2* | 867 | 0 | 0.00% | 1505 | 0 | 0.00% | . | . |
| *GNA11* | 867 | 0 | 0.00% | 1505 | 3 | 0.20% | . | . |
| *KLHL6* | 867 | 1 | 0.12% | 1505 | 0 | 0.00% | . | . |
| *STAG2* | 867 | 5 | 0.58% | 1505 | 9 | 0.60% | . | . |
| *NPM1* | 867 | 0 | 0.00% | 1505 | 2 | 0.13% | . | . |
| *ERRFI1* | 867 | 7 | 0.81% | 1505 | 24 | 1.59% | . | . |
| *JAK2* | 867 | 6 | 0.69% | 1505 | 9 | 0.60% | . | . |
| *PRSS8* | 867 | 0 | 0.00% | 1505 | 0 | 0.00% | . | . |
| *GATA3* | 867 | 0 | 0.00% | 1505 | 0 | 0.00% | . | . |
| *PHLPP2* | 867 | 0 | 0.00% | 1505 | 0 | 0.00% | . | . |
| *CRKL* | 867 | 2 | 0.23% | 1505 | 8 | 0.53% | . | . |
| *BTG1* | 867 | 0 | 0.00% | 1505 | 1 | 0.07% | . | . |
| *MERTK* | 867 | 0 | 0.00% | 1505 | 0 | 0.00% | . | . |
| *TOP2A* | 867 | 0 | 0.00% | 1505 | 0 | 0.00% | . | . |
| *ZNF217* | 867 | 5 | 0.58% | 1505 | 17 | 1.13% | . | . |
| *GEN1* | 867 | 0 | 0.00% | 1505 | 0 | 0.00% | . | . |
| *CDK6* | 867 | 6 | 0.69% | 1505 | 12 | 0.80% | . | . |
| *PARP3* | 867 | 0 | 0.00% | 1505 | 0 | 0.00% | . | . |
| *TNFAIP3* | 867 | 0 | 0.00% | 1505 | 0 | 0.00% | . | . |
| *PNRC1* | 867 | 0 | 0.00% | 1505 | 0 | 0.00% | . | . |
| *ADGRA2* | 867 | 0 | 0.00% | 1505 | 0 | 0.00% | . | . |
| *IRF4* | 867 | 0 | 0.00% | 1505 | 0 | 0.00% | . | . |
| *NT5C2* | 867 | 0 | 0.00% | 1505 | 0 | 0.00% | . | . |
| *TNF* | 867 | 0 | 0.00% | 1505 | 0 | 0.00% | . | . |
| *BCL2A1* | 867 | 0 | 0.00% | 1505 | 0 | 0.00% | . | . |
| *CRBN* | 867 | 0 | 0.00% | 1505 | 0 | 0.00% | . | . |
| *BTG2* | 867 | 0 | 0.00% | 1505 | 0 | 0.00% | . | . |
| *FBXW7* | 867 | 6 | 0.69% | 1505 | 7 | 0.47% | . | . |
| *CSF3R* | 867 | 0 | 0.00% | 1505 | 0 | 0.00% | . | . |
| *FAT1* | 867 | 0 | 0.00% | 1505 | 0 | 0.00% | . | . |
| *MSH6* | 867 | 5 | 0.58% | 1505 | 6 | 0.40% | . | . |
| *IRF2* | 867 | 9 | 1.04% | 1505 | 13 | 0.86% | . | . |
| *PDCD1* | 867 | 0 | 0.00% | 1505 | 0 | 0.00% | . | . |
| *NBN* | 867 | 4 | 0.46% | 1505 | 5 | 0.33% | . | . |
| *BRAF* | 867 | 3 | 0.35% | 1505 | 7 | 0.47% | . | . |
| *MAGI2* | 867 | 0 | 0.00% | 1505 | 0 | 0.00% | . | . |
| *FGF6* | 867 | 5 | 0.58% | 1505 | 4 | 0.27% | . | . |
| *CD74* | 867 | 0 | 0.00% | 1505 | 0 | 0.00% | . | . |
| *MAP2K1* | 867 | 2 | 0.23% | 1505 | 5 | 0.33% | . | . |
| *FANCI* | 867 | 0 | 0.00% | 1505 | 0 | 0.00% | . | . |
| *SMARCD1* | 867 | 0 | 0.00% | 1505 | 0 | 0.00% | . | . |
| *IGF2* | 867 | 0 | 0.00% | 1505 | 0 | 0.00% | . | . |
| *NKX2-1* | 867 | 0 | 0.00% | 1505 | 5 | 0.33% | . | . |
| *MAF* | 867 | 0 | 0.00% | 1505 | 0 | 0.00% | . | . |
| *CD79A* | 867 | 0 | 0.00% | 1505 | 1 | 0.07% | . | . |
| *IDH1* | 867 | 1 | 0.12% | 1505 | 5 | 0.33% | . | . |
| *GRIN2A* | 867 | 0 | 0.00% | 1505 | 0 | 0.00% | . | . |
| *PDK1* | 867 | 0 | 0.00% | 1505 | 0 | 0.00% | . | . |
| *APCDD1* | 867 | 0 | 0.00% | 1505 | 0 | 0.00% | . | . |
| *NSD3* | 867 | 0 | 0.00% | 1505 | 0 | 0.00% | . | . |
| *FAS* | 867 | 6 | 0.69% | 1505 | 13 | 0.86% | . | . |
| *EPHB1* | 867 | 0 | 0.00% | 1505 | 5 | 0.33% | . | . |
| *U2AF1* | 867 | 2 | 0.23% | 1505 | 8 | 0.53% | . | . |
| *ZRSR2* | 867 | 0 | 0.00% | 1505 | 0 | 0.00% | . | . |
| *ZNF703* | 867 | 7 | 0.81% | 1505 | 5 | 0.33% | . | . |
| *DAXX* | 867 | 1 | 0.12% | 1505 | 1 | 0.07% | . | . |
| *PMS2* | 867 | 2 | 0.23% | 1505 | 2 | 0.13% | . | . |
| *BCL2* | 867 | 1 | 0.12% | 1505 | 1 | 0.07% | . | . |
| *AMER1* | 867 | 5 | 0.58% | 1505 | 5 | 0.33% | . | . |
| *ID3* | 867 | 0 | 0.00% | 1505 | 0 | 0.00% | . | . |
| *LTK* | 867 | 1 | 0.12% | 1505 | 1 | 0.07% | . | . |
| *INPP4B* | 867 | 5 | 0.58% | 1505 | 5 | 0.33% | . | . |
| *CDKN2C* | 867 | 3 | 0.35% | 1505 | 5 | 0.33% | . | . |
| *MYCN* | 867 | 0 | 0.00% | 1505 | 4 | 0.27% | . | . |
| *EPHA6* | 867 | 0 | 0.00% | 1505 | 0 | 0.00% | . | . |
| *MAP2K2* | 867 | 0 | 0.00% | 1505 | 1 | 0.07% | . | . |
| *FGFR3* | 867 | 3 | 0.35% | 1505 | 7 | 0.47% | . | . |
| *SOX10* | 867 | 0 | 0.00% | 1505 | 0 | 0.00% | . | . |
| *SPOP* | 867 | 0 | 0.00% | 1505 | 0 | 0.00% | . | . |
| *ABL1* | 867 | 2 | 0.23% | 1505 | 1 | 0.07% | . | . |
| *RNF43* | 867 | 0 | 0.00% | 1505 | 7 | 0.47% | . | . |

**Supplementary Table 7: Co-occurrences of GA in the indicated genes, with absolute frequency of co-occurrence above 20, odds ratio above 1 and adjusted p-value below 0.05.**

| **Gene1** | **Gene2** | **Gene1(-)**  **\|gene2(-)** | **Gene1(+)**  **\|gene2(-)** | **Gene1(-)**  **\|gene2(+)** | **Gene1(+)**  **\|gene2(+)** | **Odds ratio** | **pValue (fdr)** |
| --- | --- | --- | --- | --- | --- | --- | --- |
| *CCND1* | *FGF19* | 2219 | 19 | 1 | 133 | 15533.00 | 2.57E-191 |
| *FGF19* | *FGF4* | 2237 | 30 | 1 | 104 | 7754.93 | 1.20E-148 |
| *FGF19* | *FGF3* | 2237 | 34 | 1 | 100 | 6579.41 | 1.38E-141 |
| *FGF4* | *FGF3* | 2261 | 10 | 6 | 95 | 3579.92 | 6.14E-146 |
| *CCND1* | *FGF4* | 2218 | 49 | 2 | 103 | 2331.16 | 1.58E-135 |
| *CCND1* | *FGF3* | 2218 | 53 | 2 | 99 | 2071.53 | 4.60E-129 |
| *MDM4* | *PIK3C2B* | 2332 | 6 | 9 | 25 | 1079.63 | 8.14E-43 |
| *CDKN2A* | *CDKN2B* | 2182 | 83 | 5 | 102 | 536.30 | 2.10E-116 |
| *LYN* | *MYC* | 2025 | 13 | 242 | 92 | 59.22 | 3.15E-65 |
| *NTRK1* | *MCL1* | 2250 | 26 | 59 | 37 | 54.27 | 5.35E-35 |
| *CTNNB1* | *NFE2L2* | 1529 | 718 | 35 | 90 | 5.48 | 9.57E-16 |
| *TP53* | *MET* | 1488 | 848 | 9 | 27 | 5.26 | 1.47E-3 |
| *TP53* | *CCND1* | 1452 | 768 | 45 | 107 | 4.50 | 7.18E-15 |
| *TP53* | *FGF19* | 1456 | 782 | 41 | 93 | 4.22 | 3.78E-12 |
| *TP53* | *FGF3* | 1466 | 805 | 31 | 70 | 4.11 | 1.40E-8 |
| *TP53* | *FGF4* | 1463 | 804 | 34 | 71 | 3.80 | 4.18E-8 |
| *TERT* | *LYN* | 949 | 1318 | 17 | 88 | 3.73 | 1.81E-5 |
| *TP53* | *CCNE1* | 1484 | 849 | 13 | 26 | 3.50 | 4.28E-2 |
| *TERT* | *MYC* | 900 | 1138 | 66 | 268 | 3.21 | 2.81E-15 |
| *RB1* | *TSC2* | 2099 | 159 | 92 | 22 | 3.16 | 8.78E-3 |
| *NFE2L2* | *TERT* | 942 | 24 | 1305 | 101 | 3.04 | 7.54E-05 |
| *RB1* | *PTEN* | 2090 | 158 | 101 | 23 | 3.01 | 1.05E-2 |
| *TP53* | *TSC1* | 1477 | 841 | 20 | 34 | 2.99 | 2.51E-2 |
| *MYC* | *MCL1* | 1971 | 305 | 67 | 29 | 2.80 | 9.40E-3 |
| *TP53* | *PTEN* | 1448 | 800 | 49 | 75 | 2.77 | 2.22E-5 |
| *TP53* | *TSC2* | 1452 | 806 | 45 | 69 | 2.76 | 9.44E-5 |
| *CTNNB1* | *ARID1A* | 1431 | 650 | 133 | 158 | 2.62 | 2.95E-11 |
| *CTNNB1* | *TERT* | 748 | 218 | 816 | 590 | 2.48 | 4.83E-20 |
| *TERT* | *FGF19* | 933 | 1305 | 33 | 101 | 2.19 | 2.39E-2 |
| *TERT* | *CCND1* | 927 | 1293 | 39 | 113 | 2.08 | 2.18E-2 |
| *TERT* | *ARID1A* | 886 | 1195 | 80 | 211 | 1.96 | 2.27E-4 |
| *CTNNB1* | *MYC* | 1378 | 660 | 186 | 148 | 1.66 | 9.01E-3 |

(fdr = false discovery rate)

**Supplementary Table 8: Co-occurrence of GA in indicated genes, with odds ratio below 1 and adjusted p-value below 0.05.**

| **Gene1** | **Gene2** | **Gene1(-)**  **\|gene2(-)** | **Gene1(+)**  **\|gene2(-)** | **Gene1(-)**  **\|gene2(+)** | **Gene1(+)**  **\|gene2(+)** | **Odds ratio** | **pValue (fdr)** |
| --- | --- | --- | --- | --- | --- | --- | --- |
| *TERT* | *PTEN* | 894 | 1354 | 72 | 52 | 0.48 | 2.05E-2 |
| *CTNNB1* | *TP53* | 885 | 612 | 679 | 196 | 0.42 | 1.80E-17 |
| *TP53* | *CDKN2A* | 1350 | 837 | 147 | 38 | 0.42 | 2.59E-4 |
| *CTNNB1* | *FGF19* | 1454 | 784 | 110 | 24 | 0.40 | 9.40E-3 |
| *CTNNB1* | *CCND1* | 1438 | 782 | 126 | 26 | 0.38 | 7.64E-4 |
| *NFE2L2* | *TP53* | 1393 | 104 | 854 | 21 | 0.33 | 2.06E-4 |
| *TP53* | *CDKN2B* | 1407 | 858 | 90 | 17 | 0.31 | 5.82E-4 |
| *CTNNB1* | *PTEN* | 1457 | 791 | 107 | 17 | 0.29 | 1.02E-4 |
| *CTNNB1* | *TSC2* | 1465 | 793 | 99 | 15 | 0.28 | 1.64E-4 |
| *CTNNB1* | *NTRK1* | 1509 | 800 | 55 | 8 | 0.27 | 3.52E-2 |
| *CTNNB1* | *BAP1* | 1503 | 801 | 61 | 7 | 0.22 | 2.23E-3 |
| *TERT* | *ATRX* | 950 | 1404 | 16 | 2 | 0.08 | 9.59E-3 |
| *APC* | *CTNNB1* | 1514 | 50 | 806 | 2 | 0.08 | 6.56E-05 |
| *CTNNB1* | *RB1* | 1389 | 802 | 175 | 6 | 0.06 | 1.53E-22 |
| *CTNNB1* | *CCNE1* | 1526 | 807 | 38 | 1 | 0.05 | 1.04E-3 |
| *TERT* | *BAP1* | 901 | 1403 | 65 | 3 | 0.03 | 1.83E-19 |
| TP53 | BAP1 | 1429 | 875 | 68 | 0 | 1.00E-05 | 1.68E-11 |

**Supplementary Table 9: Genomic alterations in cHCC-CCA patients.**

| **Gene** | **Incidence** | **Total Population** | **Frequency (%)** |
| --- | --- | --- | --- |
| *TP53* | 90 | 150 | 60.00 |
| *TERT* | 75 | 150 | 50.00 |
| *MYC* | 23 | 150 | 15.33 |
| *CDKN2A* | 16 | 150 | 10.67 |
| *IDH1/2* | 16 | 150 | 10.67 |
| *CCND1* | 12 | 150 | 8.00 |
| *IDH1* | 12 | 150 | 8.00 |
| *FGFR2* | 11 | 150 | 7.33 |
| *KRAS* | 11 | 150 | 7.33 |
| *FGF19* | 11 | 150 | 7.33 |
| *PTEN* | 11 | 150 | 7.33 |
| *CTNNB1* | 10 | 150 | 6.67 |
| *ARID1A* | 10 | 150 | 6.67 |
| *PIK3CA* | 10 | 150 | 6.67 |
| *STK11* | 9 | 150 | 6.00 |
| *CDKN2B* | 9 | 150 | 6.00 |
| *BRCA1/2* | 9 | 150 | 6.00 |
| *BRCA2* | 8 | 150 | 5.33 |
| *CCNE1* | 8 | 150 | 5.33 |
| *ATM* | 8 | 150 | 5.33 |
| *KEAP1* | 8 | 150 | 5.33 |
| *ERBB2* | 7 | 150 | 4.67 |
| *FGF4* | 7 | 150 | 4.67 |
| *EGFR* | 7 | 150 | 4.67 |
| *TET2* | 7 | 150 | 4.67 |
| *MCL1* | 7 | 150 | 4.67 |
| *CDK6* | 6 | 150 | 4.00 |
| *BRAF* | 5 | 150 | 3.33 |
| *MET* | 4 | 150 | 2.67 |
| *KRAS^G12D^* | 2 | 150 | 1.33 |
| *KRAS^G12C^* | 2 | 150 | 1.33 |
| *TMB-H* | 2 | 150 | 1.33 |
| *MDM2^amp^* | 1 | 150 | 0.67 |

**Supplementary Table 10: Genomic alterations in cHCC-CCA vs. HCC vs. iCCA.**

|  | **cHCC-CCA** | | **HCC** | | **iCCA** | | **pValue (fdr)** | | |
| --- | --- | --- | --- | --- | --- | --- | --- | --- | --- |
| ***Gene*** | **n/150** | **Prevalence (%)** | **n/2372** | **Prevalence (%)** | **n/6130** | **Prevalence (%)** | **cHCC-CCA vs. HCC** | **cHCC-CCA vs. iCCA** | **HCC vs. iCCA** |
| ***TP53*** | 90 | 60.0 | 875 | 36.9 | 2066 | 33.7 | **2.60E-07** | **2.21E-09** | **7.67E-03** |
| ***TERT*** | 75 | 50.0 | 1406 | 59.3 | 369 | 6.0 | 5.80E-02 | **3.34E-46** | **<2.23E-308** |
| ***MYC*** | 23 | 15.3 | 334 | 14.1 | 280 | 4.6 | 6.94 E-01 | **4.20E-06** | **3.04E-46** |
| ***CDKN2A*** | 16 | 10.7 | 185 | 7.8 | 1848 | 30.2 | 3.34E-01 | **3.49E-07** | **1.67E-120** |
| ***CCND1*** | 12 | 8.0 | 152 | 6.4 | 222 | 3.6 | 5.03E-01 | **3.58E-02** | **1.27E-07** |
| ***IDH1*** | 12 | 8.0 | 6 | 0.3 | 878 | 14.3 | **2.87E-10** | 6.20E-02 | **7.08E-121** |
| ***FGFR2*** | 11 | 7.3 | 8 | 0.3 | 713 | 11.6 | **1.27E-08** | 1.79E-01 | **1.79E-92** |
| ***KRAS*** | 11 | 7.3 | 41 | 1.7 | 1231 | 20.1 | **9.11E-04** | **1.28E-04** | **5.98E-134** |
| ***FGF19*** | 11 | 7.3 | 134 | 5.7 | 204 | 3.3 | 4.96E-01 | **4.71E-02** | **3.68E-06** |
| ***PTEN*** | 11 | 7.3 | 124 | 5.2 | 176 | 2.9 | 3.73E-01 | **1.48E-02** | **6.44E-07** |
| ***CTNNB1*** | 10 | 6.7 | 808 | 34.1 | 72 | 1.2 | **3.51E-13** | **1.21E-04** | **<2.23E-308** |
| ***ARID1A*** | 10 | 6.7 | 291 | 12.3 | 1095 | 17.9 | 6.93E-02 | **5.14E-04** | **3.31E-10** |
| ***PIK3CA*** | 10 | 6.7 | 52 | 2.2 | 400 | 6.5 | **8.31E-03** | 9.23E-01 | **2.03E-17** |
| ***STK11*** | 9 | 6.0 | 64 | 2.7 | 200 | 3.3 | 6.93E-02 | 1.55E-01 | 2.19E-01 |
| ***CDKN2B*** | 9 | 6.0 | 107 | 4.5 | 1321 | 21.6 | 5.10E-01 | **3.52E-06** | **1.86E-95** |
| ***BRCA2*** | 8 | 5.3 | 26 | 1.1 | 147 | 2.4 | **2.96E-03** | 6.20E-02 | **1.14E-04** |
| ***CCNE1*** | 8 | 5.3 | 39 | 1.6 | 141 | 2.3 | **1.42E-02** | 5.67E-02 | 7.87E-02 |
| ***ATM*** | 8 | 5.3 | 72 | 3.0 | 221 | 3.6 | 2.34E-01 | 3.36E-01 | 2.37E-01 |
| ***KEAP1*** | 8 | 5.3 | 89 | 3.8 | 72 | 1.2 | 4.96E-01 | **2.18E-03** | **4.67E-13** |
| ***ERBB2*** | 7 | 4.7 | 21 | 0.9 | 314 | 5.1 | **3.86E-03** | 1 | **6.59E-24** |
| ***FGF4*** | 7 | 4.7 | 105 | 4.4 | 187 | 3.1 | 8.64E-01 | 3.06E-01 | **2.94E-03** |
| ***EGFR*** | 7 | 4.7 | 24 | 1.0 | 136 | 2.2 | **5.92E-03** | 1.40E-01 | **2.46E-04** |
| ***TET2*** | 7 | 4.7 | 49 | 2.1 | 136 | 2.2 | 7.92E-02 | 1.40E-01 | 7.63E-01 |
| ***MCL1*** | 7 | 4.7 | 96 | 4.1 | 163 | 2.7 | 7.12E-01 | 1.82E-01 | **1.61E-03** |
| ***CDK6*** | 6 | 4.0 | 18 | 0.8 | 110 | 1.8 | **6.61E-03** | 1.07E-01 | **4.40E-04** |
| ***BRAF*** | 5 | 3.3 | 10 | 0.4 | 308 | 5.0 | **4.72E-03** | 5.48E-01 | **1.13E-31** |
| ***MET*** | 4 | 2.7 | 44 | 1.9 | 137 | 2.2 | 6.23E-01 | 6.61E-01 | 3.46E-01 |
| ***IDH2*** | 4 | 2.7 | 1 | 0.04 | 247 | 4.0 | **3.79E-04** | 6.22E-01 | **7.25E-34** |
| ***KRAS^G12D^*** | 2 | 1.3 | 3 | 0.1 | 451 | 7.4 | 6.44E-02 | **6.55E-03** | **1.89E-60** |
| ***KRAS^G12C^*** | 2 | 1.3 | 2 | 0.1 | 67 | 1.1 | **4.59E-02** | 7.49E-01 | **1.71E-07** |
| ***TMB-H*** | 2 | 1.3 | 83 | 3.5 | 227 | 3.7 | 3.56E-01 | 2.48E-01 | 7.44E-01 |
| ***BRCA1*** | 1 | 0.7 | 25 | 1.1 | 63 | 1.0 | 1 | 1 | 9.05E-01 |
| ***MDM2^amp^*** | 1 | 0.7 | 13 | 0.6 | 261 | 4.3 | 6.57E-01 | 5.06E-02 | **3.77E-23** |

Prevalence of iCCA GA were obtained from Kendre et al. 2022^41^. Adjusted *p*-values are listed (fdr = false discovery rate). Significant differences are marked in bold.

**Supplementary Table 11: Sex and age distribution according to genomic ancestry.**

| **Ancestry** | **Total (n)** | **Male (n)** | **Female (n)** | **< 65y (n)** | **≥ 65y (n)** |
| --- | --- | --- | --- | --- | --- |
| European | 1527 | 1152 | 374 | 631 | 894 |
| African | 322 | 239 | 83 | 180 | 142 |
| American | 213 | 156 | 56 | 109 | 103 |
| East Asian | 177 | 135 | 42 | 100 | 76 |
| South Asian | 36 | 26 | 10 | 15 | 21 |

**Supplementary Table 12: Distribution of TP53^R249S^ variant according to genomic ancestry in HCC.**

| **Ancestry** | **Total n** | **All *TP53* variants (n)** | **R249S (n)** | **R249S (%)** |
| --- | --- | --- | --- | --- |
| European | 1527 | 533 | 19 | 3.6 |
| African | 322 | 141 | 8 | 5.7 |
| American | 213 | 66 | 6 | 9.1 |
| East Asian | 177 | 88 | 16 | 18.2 |
| South Asian | 36 | 10 | 2 | 20.0 |

**Supplementary Table 13: Potentially actionable alterations in HCC.**

| **Gene** | **Patients (n) in the HCC cohort** | **Drug** | **ESCAT** | **Reference** |
| --- | --- | --- | --- | --- |
| *FGF19^amp^* | 134 | irpagratinib (ABSK-011)  lenvatinib | IC  IIIb / I | ^1^ |
| *TSC2*   - *TSC2^SV^* - *TSC2^del^* - *TSC2^RE^* | 88  20  7 | mTOR inhibitors (e.g. everolimus) | II | ^2^  ^3^ |
| *TMB-H* | 83 | immune checkpoint inhibition | III | ^4^ |
| *BRCA1*   - *BRCA1^SV^* - *BRCA1^RE^* - *BRCA1^del^* | 12  10  3 | PARP inhibitors (e.g. olaparib) | III | ^5^ |
| *BRCA2*   - *BRCA2^SV^* - *BRCA2^del^* - *BRCA2^RE^* | 21  3  2 | PARP inhibitors (e.g. olaparib) | III | ^5^ |
| *MET*   - *MET^amp^* - *(MET^SV^)* - *MET^exon14 skipping^* | 36  (6)  6 | capmatinib  tepotinib | III | ^6^  ^7^ |
| *CCNE1^amp^* | 38 | lunresertib | III | ^8^ |
| *ERBB2*   - *ERBB2^amp^* - *(ERBB^SV^)* | 19  (2) | trastuzumab  trastuzumab-deruxtecan  trastuzumab/tucatinib  zanidatamab  trastuzumab/pertuzumab | III | ^9^  ^10^  ^11^  ^12^  ^13^ |
| *MDM2^amp^* | 13 | brigimadlin | III | ^14^ |
| *FBXW7* | 13 | lunresertib | III | ^8^ |
| *EGFR^amp^* | 11 | lenvatinib combination therapies | V | ^15^ |
| *BRAF*   - *(BRAF^non-V600E-SV^)* - *(BRAF^amp^)* - *BRAF^V600E^* | (6)  (3)  1 | encorafenib  vemurafenib  dabrafenib | III | ^16^  ^17^  ^18^  ^19^ |
| *TP53^Y220C^* | 7 | rezatapopt (PC14586) | III | ^20^ |
| *MSI-H* | 3 | immune checkpoint inhibition | IC | ^21^ |
| *NTRK^RE^* | 3 | larotrectinib  entrectinib | IC | ^22^  ^23^ |
| *ROS1^RE^* | 3 | crizotinib  entrectinib  repotrectinib | III | ^24^  ^25^  ^26^ |
| *KRAS*   - *KRAS^G12C^* - *KRAS^G12D^* | 2  3 | sotorasib  adagrasib  ASP3082  zoldonrasib | III/ IC | ^27^  ^28,29^  ^30^  ^31^ |
| *FGFR2^RE^* | 1 | pemigatinib  futibatinib  erdafitinib  lirafugratinib  tinengotinib | III | ^32^  ^33^  ^34^  ^35^  ^36^ |
| *ALK^RE^* | 1 | crizotinib  entrectinib  alectinib | III | ^37^  ^23^  ^38^ |
| *RET^RE^* | 1 | selpercatinib | III | ^39^ |

For each genetic alteration (amp: amplification; del: deletion; RE: rearrangement/fusion; SV: single variation) the potential drug is listed and the according ESMO Scale for Clinical Actionability of Molecular Targets (ESCAT) level ^40^).

**Supplementary Table 14: *TP53* or *TERT* alterations in patients with detection of HBV integration, with odds ratio above or below 1.**

| **Gene1** | **Gene2**  ***** | **Gene1(-) \|gene2(-)** | **Gene1(+)**  **\|gene2(-)** | **Gene1(-)**  **\|gene2(+)** | **Gene1(+)**  **\|gene2(+)** | **Odds ratio** | **pValue (fdr)** |
| --- | --- | --- | --- | --- | --- | --- | --- |
| *TP53* | *HBV* | 1421 | 774 | 76 | 101 | 2.44 | 7.22E-06 |
| *TERT* | *HBV* | 847 | 1348 | 119 | 58 | 0.31 | 1.56E-10 |

Adjusted p-values are listed (fdr = false discovery rate).

*detected HBV integration

# **Supplementary References**

1. Cheng Q, Zhang Y, Wang J, et al. 149MO Irpagratinib (ABSK-011) plus atezolizumab in first-line (1L) and immune checkpoint inhibitors (ICIs) treated advanced hepatocellular carcinoma (HCC) with FGF19 overexpression (+): Updated results of the phase II ABSK-011-201 study. *Annals of Oncology*. 2025;36:S63. doi:10.1016/j.annonc.2025.05.162

2. Huynh H, Hao HX, Chan SL, et al. Loss of Tuberous Sclerosis Complex 2 (TSC2) Is Frequent in Hepatocellular Carcinoma and Predicts Response to mTORC1 Inhibitor Everolimus. *Mol Cancer Ther*. May 2015;14(5):1224-35. doi:10.1158/1535-7163.MCT-14-0768

3. Limousin W, Laurent-Puig P, Ziol M, et al. Molecular-based targeted therapies in patients with hepatocellular carcinoma and hepato-cholangiocarcinoma refractory to atezolizumab/bevacizumab. *J Hepatol*. Dec 2023;79(6):1450-1458. doi:10.1016/j.jhep.2023.08.017

4. Marabelle A, Le DT, Ascierto PA, et al. Efficacy of Pembrolizumab in Patients With Noncolorectal High Microsatellite Instability/Mismatch Repair-Deficient Cancer: Results From the Phase II KEYNOTE-158 Study. *J Clin Oncol*. Jan 1 2020;38(1):1-10. doi:10.1200/JCO.19.02105

5. Golan T, Hammel P, Reni M, et al. Maintenance Olaparib for Germline BRCA-Mutated Metastatic Pancreatic Cancer. *N Engl J Med*. Jul 25 2019;381(4):317-327. doi:10.1056/NEJMoa1903387

6. Wolf J, Seto T, Han JY, et al. Capmatinib in MET Exon 14-Mutated or MET-Amplified Non-Small-Cell Lung Cancer. *N Engl J Med*. Sep 3 2020;383(10):944-957. doi:10.1056/NEJMoa2002787

7. Paik PK, Felip E, Veillon R, et al. Tepotinib in Non-Small-Cell Lung Cancer with MET Exon 14 Skipping Mutations. *N Engl J Med*. Sep 3 2020;383(10):931-943. doi:10.1056/NEJMoa2004407

8. Schram AM, Lee EK, Højgaard M, et al. Abstract CT262: Efficacy and safety of the combination PKMYT1-inhibitor lunresertib and ATR-inhibitor camonsertib in patients with ovarian and endometrial cancers: Phase I MYTHIC study (NCT04855656). *Cancer Research*. 2025;85(8_Supplement_2):CT262-CT262. doi:10.1158/1538-7445.AM2025-CT262

9. Slamon DJ, Leyland-Jones B, Shak S, et al. Use of chemotherapy plus a monoclonal antibody against HER2 for metastatic breast cancer that overexpresses HER2. *N Engl J Med*. Mar 15 2001;344(11):783-92. doi:10.1056/NEJM200103153441101

10. Ohba A, Morizane C, Kawamoto Y, et al. Trastuzumab Deruxtecan in Human Epidermal Growth Factor Receptor 2–Expressing Biliary Tract Cancer (HERB; NCCH1805): A Multicenter, Single-Arm, Phase II Trial. *Journal of Clinical Oncology*. 2024;42(27):3207-3217. doi:10.1200/jco.23.02010

11. Nakamura Y, Mizuno N, Sunakawa Y, et al. Tucatinib and trastuzumab for previously treated HER2-positive metastatic biliary tract cancer (SGNTUC-019): A phase 2 basket study. *Journal of Clinical Oncology*. 2023;41(16_suppl):4007-4007. doi:10.1200/JCO.2023.41.16_suppl.4007

12. Harding JJ, Fan J, Oh DY, et al. Zanidatamab for HER2-amplified, unresectable, locally advanced or metastatic biliary tract cancer (HERIZON-BTC-01): a multicentre, single-arm, phase 2b study. *Lancet Oncol*. Jul 2023;24(7):772-782. doi:10.1016/S1470-2045(23)00242-5

13. Javle M, Borad MJ, Azad NS, et al. Pertuzumab and trastuzumab for HER2-positive, metastatic biliary tract cancer (MyPathway): a multicentre, open-label, phase 2a, multiple basket study. *Lancet Oncol*. Sep 2021;22(9):1290-1300. doi:10.1016/S1470-2045(21)00336-3

14. Reichardt P, Schöffski P, Lorusso P, et al. 61MO Phase Ia/Ib study of the MDM2-p53 antagonist brigimadlin (BI 907828) in advanced solid tumours: Overall safety, and efficacy in patients (pts) with well-differentiated liposarcoma (WDLPS). *ESMO Open*. 2024;9doi:10.1016/j.esmoop.2024.102451

15. Jin H, Shi Y, Lv Y, et al. EGFR activation limits the response of liver cancer to lenvatinib. *Nature*. Jul 2021;595(7869):730-734. doi:10.1038/s41586-021-03741-7

16. Kopetz S, Grothey A, Yaeger R, et al. Encorafenib, Binimetinib, and Cetuximab in BRAF V600E-Mutated Colorectal Cancer. *N Engl J Med*. Oct 24 2019;381(17):1632-1643. doi:10.1056/NEJMoa1908075

17. Chapman PB, Hauschild A, Robert C, et al. Improved survival with vemurafenib in melanoma with BRAF V600E mutation. *N Engl J Med*. Jun 30 2011;364(26):2507-16. doi:10.1056/NEJMoa1103782

18. Robert C, Grob JJ, Stroyakovskiy D, et al. Five-Year Outcomes with Dabrafenib plus Trametinib in Metastatic Melanoma. *N Engl J Med*. Aug 15 2019;381(7):626-636. doi:10.1056/NEJMoa1904059

19. Subbiah V, Kreitman RJ, Wainberg ZA, et al. Dabrafenib plus trametinib in BRAFV600E-mutated rare cancers: the phase 2 ROAR trial. *Nat Med*. May 2023;29(5):1103-1112. doi:10.1038/s41591-023-02321-8

20. Dumbrava EE, Johnson ML, Tolcher AW, et al. First-in-human study of PC14586, a small molecule structural corrector of Y220C mutant p53, in patients with advanced solid tumors harboring a <i>TP53</i> Y220C mutation. *Journal of Clinical Oncology*. 2022;40(16_suppl):3003-3003. doi:10.1200/JCO.2022.40.16_suppl.3003

21. Marcus L, Lemery SJ, Keegan P, Pazdur R. FDA Approval Summary: Pembrolizumab for the Treatment of Microsatellite Instability-High Solid Tumors. *Clin Cancer Res*. Jul 1 2019;25(13):3753-3758. doi:10.1158/1078-0432.CCR-18-4070

22. Drilon A, Laetsch TW, Kummar S, et al. Efficacy of Larotrectinib in TRK Fusion-Positive Cancers in Adults and Children. *N Engl J Med*. Feb 22 2018;378(8):731-739. doi:10.1056/NEJMoa1714448

23. Drilon A, Siena S, Ou SI, et al. Safety and Antitumor Activity of the Multitargeted Pan-TRK, ROS1, and ALK Inhibitor Entrectinib: Combined Results from Two Phase I Trials (ALKA-372-001 and STARTRK-1). *Cancer Discov*. Apr 2017;7(4):400-409. doi:10.1158/2159-8290.CD-16-1237

24. Shaw AT, Ou SH, Bang YJ, et al. Crizotinib in ROS1-rearranged non-small-cell lung cancer. *N Engl J Med*. Nov 20 2014;371(21):1963-71. doi:10.1056/NEJMoa1406766

25. Dziadziuszko R, Krebs MG, De Braud F, et al. Updated Integrated Analysis of the Efficacy and Safety of Entrectinib in Locally Advanced or Metastatic ROS1 Fusion-Positive Non-Small-Cell Lung Cancer. *J Clin Oncol*. Apr 10 2021;39(11):1253-1263. doi:10.1200/JCO.20.03025

26. Drilon A, Camidge DR, Lin JJ, et al. Repotrectinib in ROS1 Fusion-Positive Non-Small-Cell Lung Cancer. *N Engl J Med*. Jan 11 2024;390(2):118-131. doi:10.1056/NEJMoa2302299

27. de Langen AJ, Johnson ML, Mazieres J, et al. Sotorasib versus docetaxel for previously treated non-small-cell lung cancer with KRAS(G12C) mutation: a randomised, open-label, phase 3 trial. *Lancet*. Mar 4 2023;401(10378):733-746. doi:10.1016/S0140-6736(23)00221-0

28. Jänne PA, Riely GJ, Gadgeel SM, et al. Adagrasib in Non-Small-Cell Lung Cancer Harboring a KRAS(G12C) Mutation. *N Engl J Med*. Jul 14 2022;387(2):120-131. doi:10.1056/NEJMoa2204619

29. Bekaii-Saab TS, Yaeger R, Spira AI, et al. Adagrasib in Advanced Solid Tumors Harboring a KRAS(G12C) Mutation. *J Clin Oncol*. Sep 1 2023;41(25):4097-4106. doi:10.1200/jco.23.00434

30. Park W, Kasi A, Spira AI, et al. 608O Preliminary safety and clinical activity of ASP3082, a first-in-class, KRAS G12D selective protein degrader in adults with advanced pancreatic (PC), colorectal (CRC), and non-small cell lung cancer (NSCLC). *Annals of Oncology*. 2024;35:S486-S487. doi:10.1016/j.annonc.2024.08.675

31. Arbour KC, Tawee T, Yaeger R, et al. Abstract CT019: Preliminary safety and antitumor activity of zoldonrasib (RMC-9805), an oral, RAS(ON) G12D-selective, tri-complex inhibitor in patients with KRAS G12D non-small cell lung cancer (NSCLC) from a phase 1 study in advanced solid tumors. *Cancer Research*. 2025;85(8_Supplement_2):CT019-CT019. doi:10.1158/1538-7445.Am2025-ct019

32. Abou-Alfa GK, Sahai V, Hollebecque A, et al. Pemigatinib for previously treated, locally advanced or metastatic cholangiocarcinoma: a multicentre, open-label, phase 2 study. *Lancet Oncol*. May 2020;21(5):671-684. doi:10.1016/S1470-2045(20)30109-1

33. Goyal L, Meric-Bernstam F, Hollebecque A, et al. Futibatinib for FGFR2-Rearranged Intrahepatic Cholangiocarcinoma. *N Engl J Med*. Jan 19 2023;388(3):228-239. doi:10.1056/NEJMoa2206834

34. Loriot Y, Matsubara N, Park SH, et al. Erdafitinib or Chemotherapy in Advanced or Metastatic Urothelial Carcinoma. *N Engl J Med*. Nov 23 2023;389(21):1961-1971. doi:10.1056/NEJMoa2308849

35. Subbiah V, Sahai V, Maglic D, et al. RLY-4008, the First Highly Selective FGFR2 Inhibitor with Activity across FGFR2 Alterations and Resistance Mutations. *Cancer Discov*. Sep 6 2023;13(9):2012-2031. doi:10.1158/2159-8290.Cd-23-0475

36. Fountzilas C, Liao C-Y, Pelster M, et al. Tinengotinib in patients with advanced, metastatic cholangiocarcinoma: Overall survival results and biomarker correlative analysis from a phase 2 clinical trial. *Journal of Clinical Oncology*. 2025;43(4_suppl):608-608. doi:10.1200/JCO.2025.43.4_suppl.608

37. Shaw AT, Kim DW, Nakagawa K, et al. Crizotinib versus chemotherapy in advanced ALK-positive lung cancer. *N Engl J Med*. Jun 20 2013;368(25):2385-94. doi:10.1056/NEJMoa1214886

38. Peters S, Camidge DR, Shaw AT, et al. Alectinib versus Crizotinib in Untreated ALK-Positive Non-Small-Cell Lung Cancer. *N Engl J Med*. Aug 31 2017;377(9):829-838. doi:10.1056/NEJMoa1704795

39. Subbiah V, Wolf J, Konda B, et al. Tumour-agnostic efficacy and safety of selpercatinib in patients with RET fusion-positive solid tumours other than lung or thyroid tumours (LIBRETTO-001): a phase 1/2, open-label, basket trial. *Lancet Oncol*. Oct 2022;23(10):1261-1273. doi:10.1016/S1470-2045(22)00541-1

40. Mosele F, Remon J, Mateo J, et al. Recommendations for the use of next-generation sequencing (NGS) for patients with metastatic cancers: a report from the ESMO Precision Medicine Working Group. *Ann Oncol*. Nov 2020;31(11):1491-1505. doi:10.1016/j.annonc.2020.07.014

41. Kendre G, Murugesan K, Brummer T, Segatto O, Saborowski A, Vogel A. Charting co-mutation patterns associated with actionable drivers in intrahepatic cholangiocarcinoma. *J Hepatol*. Mar 2023;78(3):614-626. doi:10.1016/j.jhep.2022.11.030
