# Supplementary material for: Mutational patterns and ancestry-linked profiles in a large hepatocellular carcinoma and combined hepatocellular–cholangiocarcinoma cohort
Source: ESMO Open. 2026 Jan 20;11(2):106048. doi: 10.1016/j.esmoop.2025.106048 (PMC12857332; doi:10.1016/j.esmoop.2025.106048)
Supplement: Supplementary Figures [file mmc3.pdf]

Mutational Patterns and Ancestry-Linked Profiles in a Large Hepatocellular Carcinoma and Combined Hepatocellular-Cholangiocarcinoma Cohort

Christoph Gerdes*, Shruthi Rengarajan*, Karthikeyan Murugesan, Jeffrey S Ross, Stephan Bartels, Arndt Vogel† and Anna Saborowski†

* equal contribution

† equal contribution

Supplementary Figure 1

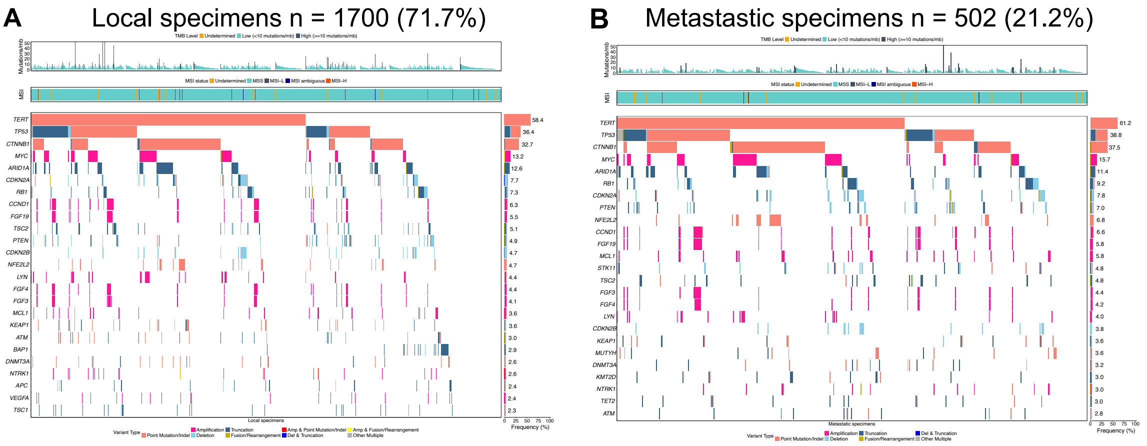

Supp. Fig. 1. Tileplots according to indicated specimen site. Tileplots of the top 25 GA in the (A) local vs. (B) metastatic specimens. Absolute patient numbers and percentages are indicated above.

Supplementary Figure 2

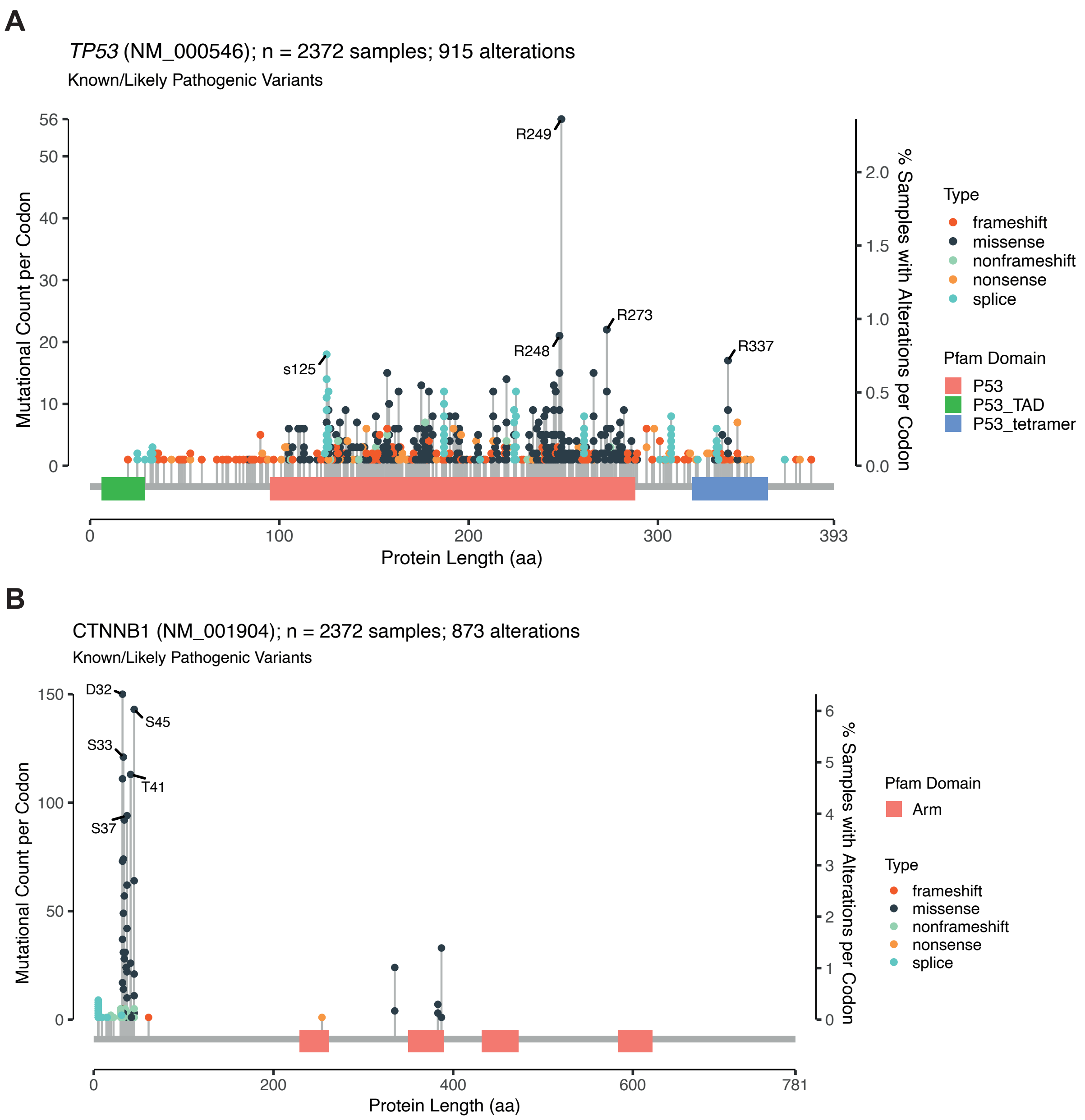

Supp. Fig. 2. Lollipop plot illustrating *TP53* (A) and *CTNNB1* (B) variants in the HCC cohort. TAD: transactivation domain. Tetramer: tetramerization domain. Arm: Armadillo repeats.

Supplementary Figure 3

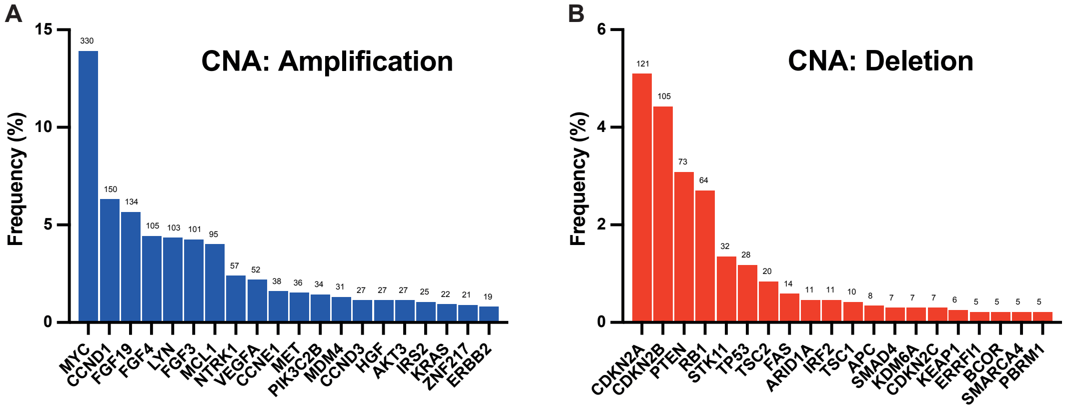

Supp. Fig. 3. Recurrent copy number alterations. (A/B) Bar chart indicates frequency of copy number amplifications (A) and deletions (B) in the HCC cohort. Absolute numbers are shown above bars.

Supplementary Figure 4

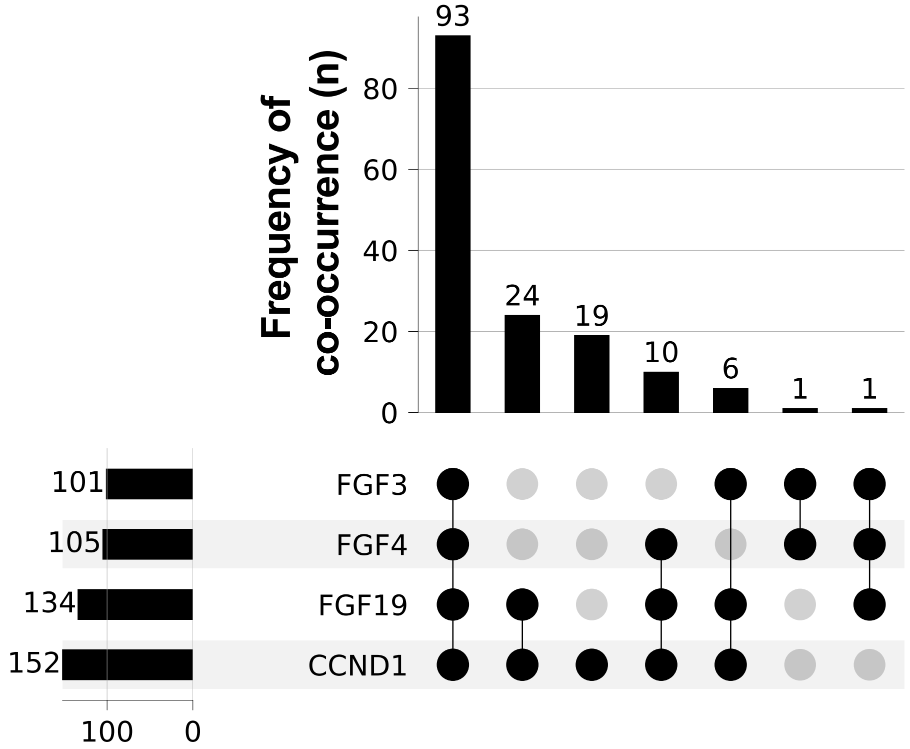

Supp. Fig. 4. Upset plot of *CCND1* and *FGF* co-occurrences. Numbers next to bars and y-axis indicate absolute counts.

Supplementary Figure 5

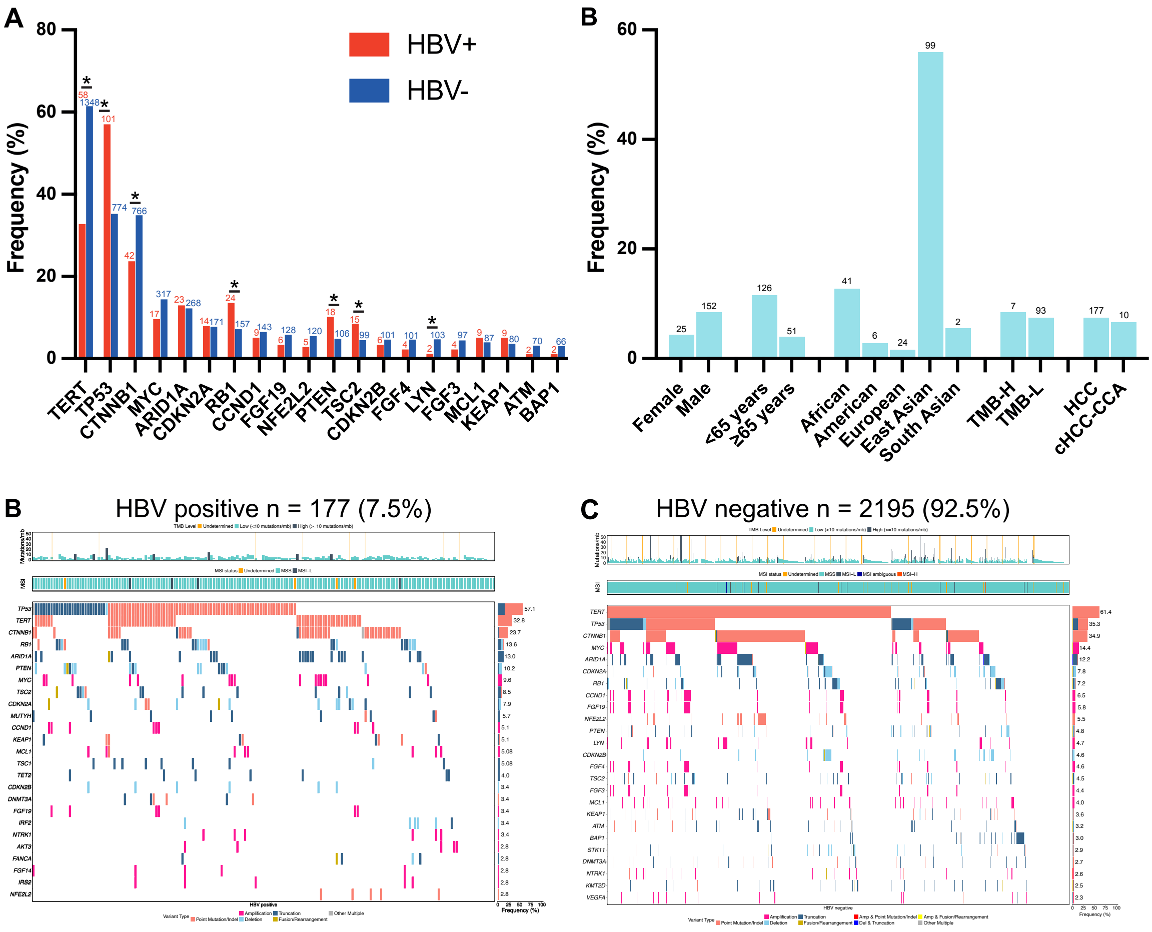

Supp. Fig. 5. Genomic alterations in patients with HBV DNA integration. (A) Frequency of the top 20 GA according to HBV status, absolute numbers are displayed above bars. HBV+/- indicates cases with and without detected HBV DNA integration, respectively. (B) Frequency of detected HBV DNA integration according to indicated subgroups, absolute numbers are displayed above bars. (C), (D) Tileplots of the top 25 GA according to detected HBV status. Absolute patient numbers and percentages are indicated above. *indicates $p<0.05$.

Supplementary Figure 6

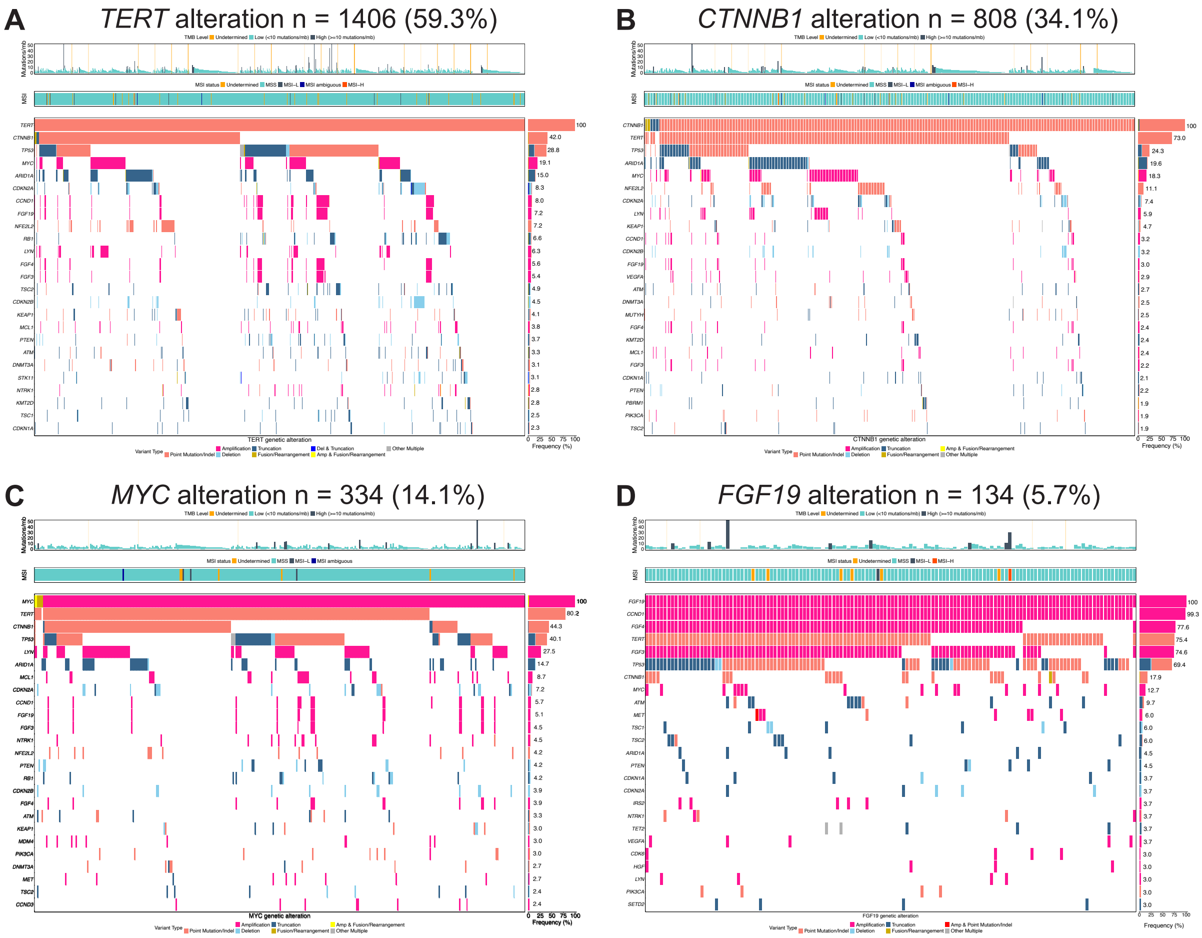

Supp. Fig. 6. Tileplots according to indicated mutational status. (A – D) Tileplots of the top 25 GA in the HCC samples with *TERT*, *CTNNB1*, *MYC*, and *FGF19* GA.
